# m^6^A reader Ythdf proteins control retrotransposon B2 repeat expression and safeguard early embryo development

**DOI:** 10.1038/s44318-026-00728-w

**Published:** 2026-03-11

**Authors:** Ruibao Su, Di Gao, Zongchang Du, Tie-Gang Meng, Chao Li, Lei-Ning Chen, Xiaoting Lin, Changchang Cao, Li-Hua Fan, Yanbin Dong, Sheng Li, Shi-Ming Luo, Shuai Jiang, Zhong Guo, Yulu Tian, Qing-Yuan Sun, Xiang-Hong Ou

**Affiliations:** 1 Guangzhou Key Laboratory of Metabolic Diseases and Reproductive Health, Guangdong-Hong Kong Metabolism & Reproduction Joint Laboratory, Reproductive Medicine Center, the Affiliated Guangdong Second Provincial General Hospital of Jinan University, 510317 Guangzhou, China; 2 Department of Obstetrics and Gynecology, Reproductive Medicine Center, The First Affiliated Hospital of Bengbu Medical University, 233004 Bengbu, China; 3 School of Artificial Intelligence, University of Chinese Academy of Sciences, 100049 Beijing, China; 4 State Key Laboratory of Cardiovascular Disease, Fuwai Hospital, National Center for Cardiovascular Diseases, Chinese Academy of Medical Sciences and Peking Union Medical College, 100037 Beijing, China; 5 Key Laboratory of RNA Biology, Institute of Biophysics, Chinese Academy of Sciences, 100101 Beijing, China; 6 Henan Academy of Innovations in Medical Science, 451163 Zhengzhou, China; 7 Center for Biological Science and Technology, Advanced Institute of Natural Sciences, Beijing Normal University, 519087 Zhuhai, China; 8 Shaanxi Key Laboratory of Earth Surface System and Environmental Carrying Capacity, College of Urban and Environmental Sciences, Northwest University, 710127 Xi’an, China; 9 Key Laboratory of Regenerative Medicine of Ministry of Education, Jinan University, 510632 Guangzhou, China; 10 Department of Developmental Biology, School of Basic Medical Sciences, Southern Medical University, 510515 Guangzhou, China

**Keywords:** Ythdf1/2/3 Proteins, RNA Decay, SINE/B2 Retrotransposons, RNA Polymerase Ⅱ Transcription, Early Embryo, Development, RNA Biology

## Abstract

*N*^6^-methyladenosine (m^6^A) and its binding proteins are critical regulators of gene expression and development in mammals. Despite its extent and importance, the regulatory mechanisms of m^6^A-binding reader proteins Ythdf1, Ythdf2, and Ythdf3 during the early stages of mammalian development remain incompletely understood. Here, we show that Ythdf2 and Ythdf3, but not Ythdf1, are required for early embryo development in mice. Mechanically, we demonstrate that all three Ythdf proteins mediate the decay of their target transcripts by binding to similar m^6^A sites, including maternal mRNAs, mid-preimplantation-activated transcripts, and retrotransposon RNAs. Among these, retrotransposon B2 RNAs emerge as one of the primary targets of Ythdf proteins throughout early embryo development, and deficiency in Ythdf1-3 leads to the accumulation of SINE/B2 RNAs, which, in turn, attenuates RNA polymerase II (Pol II) transcription through *trans*-regulatory mechanisms. In parallel, Ythdf1-3 deficiency represses Pol III-driven B2 transcription, thereby modulating RNA polymerase II activity at genomic regions adjacent to B2 loci via *cis*-regulatory effects. Together, the coordinated regulatory axis of Ythdf-SINE/B2-gene expression governs a broad transcriptional network that is crucial for embryogenesis.

## Synopsis

The functional and regulatory roles of m^6^A reader Ythdf1/2/3 proteins in early mammalian development remain incompletely understood. This work shows that a coordinated Ythdf–SINE/B2 gene-regulatory axis is essential for embryogenesis.

- Ythdf2 and Ythdf3, but not Ythdf1, are required for early mouse embryo development.
- All three Ythdf proteins target transcripts such as maternal mRNAs, mid-preimplantation-activated transcripts, and retrotransposon RNAs for decay.
- SINE/B2 RNAs accumulation in the absence of Ythdf1-3 attenuates RNA Polymerase II transcription both through *trans*- and *cis*-regulatory mechanisms.
- Depletion of B2 RNA largely rescues developmental defects observed in Ythdf1-3 knockdown embryos.
- The coordinated regulatory axis of Ythdf-SINE/B2-gene expression governs a broad transcriptional network that is crucial for embryogenesis.

## Abstract (web summary)

Ythdf proteins regulate SINE/B2 repeat expression to preserve transcriptional homeostasis during early embryogenesis.

## Graphical abstract

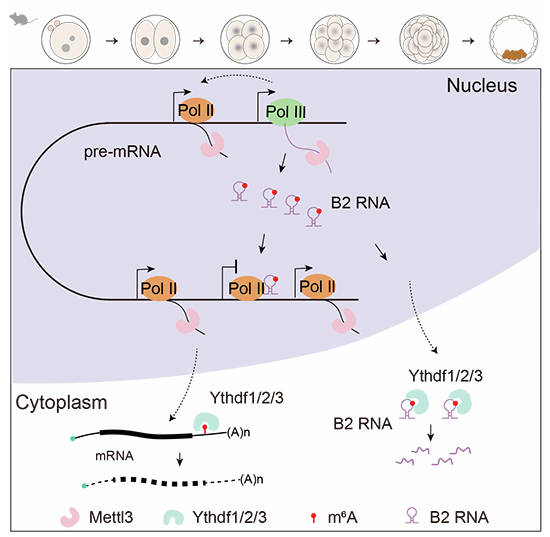

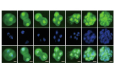

## Introduction

*N*^6^-methyladenosine (m^6^A) is one of the most important RNA modifications in the eukaryotic transcriptome (Boccaletto et al, 2018). m^6^A and its associated binding proteins play critical roles in regulating the spatiotemporal gene expression during various developmental processes (Mu et al, 2021; Wei et al, 2022; Wu et al, 2022), including the maternal-to-zygotic (MZT) transition, which involves the timely decay of maternal transcripts and subsequent massive activation of zygotic transcripts (Lee et al, 2014). In mammalian oocytes and embryos, m^6^A is deposited on messenger RNAs, repeat RNAs, and long noncoding RNAs (Mu et al, 2021; Wang et al, 2023; Yao et al, 2023; Zhu et al, 2023), regulated by writer and eraser proteins, affecting transcript fate through reader proteins (Shi et al, 2019). The cytosolic m^6^A reader proteins of the YT521-B homology Domain-containing Family (YTHDF), which include YTHDF1, YTHDF2, and YTHDF3, directly recognize m^6^A sites through the YTH domain (Luo and Tong, 2014). In the classical model, each YTHDF protein has different reported functions: YTHDF1 enhances the translation of its mRNA targets (Wang et al, 2015), YTHDF2 promotes the decay of its mRNA targets(Wang et al, 2014), and YTHDF3 facilitates both translation and degradation of its mRNA targets (Li et al, 2017; Shi et al, 2017). Moreover, YTHDF proteins regulate the translation and degradation of m^6^A-modified mRNAs in a context-dependent manner, wherein O-GlcNAcylation of YTHDF1/3 proteins modulates YTHDF1/3-mediated translation (Chen et al, 2023). However, several reports argue that the three YTHDF proteins act redundantly to mediate the same mRNA degradation, because of the similar binding affinity for m^6^A-containing RNA for all three YTHDF proteins. And yet, the function of YTHDF1 and YTHDF3 in enhancing translation is not detectable, considering the redundant function of RNA decay on m^6^A-modified mRNA is dosage-dependent (Kontur et al, 2020; Lasman et al, 2020; Zaccara and Jaffrey, 2020; Zou et al, 2023). Therefore, the precise roles of YTHDF proteins remain under debate.

Approximately half of the mammalian genome is derived from transposon elements (TEs), including three major classes of retrotransposons in mammals: long terminal repeat (LTR) elements, long interspersed nuclear elements (LINEs), and short interspersed nuclear elements (SINEs), which are widely recognized as key drivers of genome evolution due to their ability to rewire gene regulatory networks. In embryonic stem (ES) cells and early embryos, many classes of autonomous TEs are still actively expressed and marked with m^6^A by the METTL3-METTL14 complex (Deng et al, 2014). YTHDC1 binds to the transcripts of IAP, ERVK, and LINE1 retrotransposons in mouse ES cells in an m^6^A-dependent manner, mediating chromatin modulation via H3K9me3 and functional regulation through retrotransposon repression (Liu et al, 2021; Xu et al, 2021). Moreover, methylation of ERV mRNAs mainly acts by reducing the half-life of IAP mRNA with the recruitment of the m^6^A reader YTHDF proteins, thereby providing a protective effect in maintaining cellular integrity by clearing reactive ERV-derived RNA species (Chelmicki et al, 2021). FTO mediates m^6^A demethylation of LINE1 RNA in mouse ES cells and early embryos, regulating LINE1 abundance and the local chromatin state, which in turn modulates transcription of LINE1-containing genes (Wei et al, 2022). In brief, m^6^A modifications on chromosome-associated regulatory RNAs, including promoter-associated RNAs, enhancer RNAs, and repeat RNAs, can tune chromatin state and transcription globally (Liu et al, 2020b).

In mice, conditional knockout (cKO) of *Ythdf2* (*Ythdf2*^HA-Fl/HA-Fl^; *Zp3*-Cre) leads to female-specific infertility, and the embryos mainly arrest at the 2-cell (2C) stage (Ivanova et al, 2017). Both *Ythdf1* KO and *Ythdf3* KO mice are fertile and exhibit normal morphology in their reproductive organs (Lasman et al, 2020). Due to the limited research materials from embryos and technical challenges, the regulatory circuitry mechanisms by which Ythdf proteins regulate the transcriptome after fertilization and impact embryonic development in mammals remain unknown. Here, we demonstrate that all Ythdf proteins mediate the decay of their target retrotransposon RNAs and mRNAs. The coordinated regulatory axis of Ythdf-SINE/B2-gene expression is essential for proper early embryonic development.

## Results

### *Ythdf* knockdown leads to defects in embryonic development

**Figure 1:**
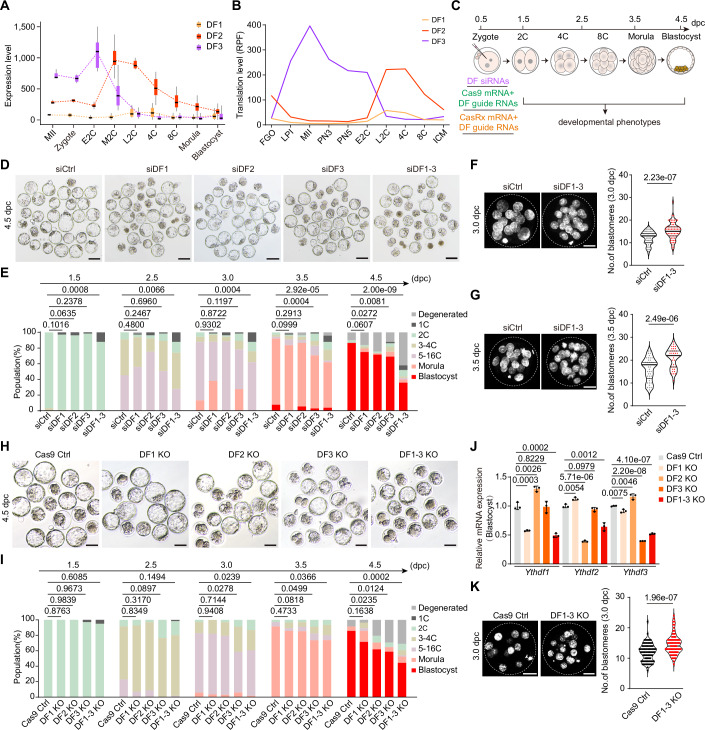
Developmental effects of *Ythdf* KD or KO. (**A**) Box plots showing *Ythdf1*, *Ythdf2*, and *Ythdf3* mRNA levels in early embryos from a previous publication (Deng et al, 2014). MⅡ oocytes, *n* = 3; Zygote, *n* = 4; Early 2-Cell (E2C), *n* = 8; Middle 2-Cell (M2C), *n* = 12; Late 2-Cell (L2C), *n* = 10; 4-cell (4C), *n* = 14; 8-cell (8C), *n* = 47; Morula (MO), *n* = 58; Blastocyst (BL), *n* = 60. Boxes represent the 25th-75th percentile (line at the median), with whiskers at 1.5× interquartile range (IQR). (**B**) Line plots showing YTHDF translation levels during early embryo development from a previous publication (Xiong et al, 2022). RPF ribosome-protected fragment. (**C**) Schematic of the experimental procedure for microinjecting *Ythdf* siRNAs, CRISPR/Cas9 guide RNAs, and CasRx guide RNAs to zygotes and culturing embryos in vitro. (**D**) Representative images showing embryos treated with *Ythdf* siRNAs or control siRNA at 4.5 dpc. Representative images were selected from two to four independent experiments. Scale bars, 100 μm. (**E**) Percentages of embryonic stages observed at the indicated time points in siControl (siCtrl, *n* = 90), si*Ythdf1*(siDF1, *n* = 68), si*Ythdf2* (siDF2, *n* = 53), si*Ythdf3* (siDF3, *n* = 65), and si*Ythdf1-3* (siDF1-3, *n* = 50) groups. *P* values were determined by the Chi-square test. (**F**, **G**) Representative images of immunofluorescence staining of DAPI (4,6-diamidino-2-phenylindole) in control and *Ythdf1-3* knockdown morula at 3.0 dpc (**F**) and 3.5 dpc (**G**). Scale bars, 20 µm. Violin plots showing the total number of blastomeres per embryo in control and *Ythdf1-3* knockdown morulae at 3.0 (siCtrl, *n* = 67; siDF1-3, *n* = 75) and 3.5 (siCtrl, *n* = 47; siDF1-3, *n* = 39) dpc. The upper and lower lines in the violin plots represent upper and lower quartiles (25th and 75th percentiles), and the center line represents the median. (**H**) Representative images showing embryos treated with *Ythdf* guide RNAs (CRISPR/Cas9) or control guide RNA at 4.5 dpc. Representative images were selected from 2 independent experiments. Scale bars, 100 μm. (**I**) Percentages of embryonic stages observed at the indicated time points in Cas9 Control (Cas9 Ctrl, *n* = 36), DF1 KO (*n* = 29), DF2 KO (*n* = 35), DF3 KO (*n* = 34), and DF1-3 KO (*n* = 61) groups. *P* values (**E**, **I**) were determined by the Chi-square test. (**J**) Relative expression of *Ythdf1/2/3* quantified by RT-qPCR when single DF or triple DF KO in the blastocyst embryos. Data are mean ± SD, *n* = 3 biological replicates. The *P* value was determined by a two-tailed unpaired *t* test. (**K**) Representative images of immunofluorescence staining of DAPI in Cas9 control (*n* = 86) and Cas9-mediated DF1-3 KO morula (*n* = 79) at 3.0 dpc. Scale bars, 20 µm. Violin plots showing the total number of blastomeres per embryo in control and DF1-3 KO morulae at 3.0 dpc. The upper and lower lines in the violin plots represent upper and lower quartiles (25th and 75th percentiles), and the center line represents the median. *P* values in (**F**, **G**, **K**) were determined by a two-tailed unpaired *t* test. Source data are available online for this figure.

To understand the function and mechanism of Ythdf1/2/3 (hereafter referred to as DF1, DF2, and DF3) as regulators in preimplantation development, we first examined their dynamic mRNA expression patterns in oocytes and early mouse embryos. *Ythdf1/2/3* transcripts exhibit distinct expression patterns, with the expression of *Ythdf3* and *Ythdf2* culminating in the early and middle 2-cell (2C) stages, respectively. Afterward, their expression gradually declines until the blastocyst stage. In contrast, the expression of *Ythdf1* remains consistently low throughout early development, with a slight increase in late 2-cell (L2C) embryos before gradually declining in 8-cell (8C) embryos (Deng et al, 2014) (Fig. 1A). The translation dynamics of *Ythdf1-3* mirrored their corresponding RNA levels during the early development, except for Ythdf3, whose translational pattern in oocytes appeared to diverge from its RNA expression profile (Xiong et al, 2022) (Fig. 1B). Therefore, DF1/2/3 proteins have a different expression pattern during early embryonic development.

**Figure EV1:**
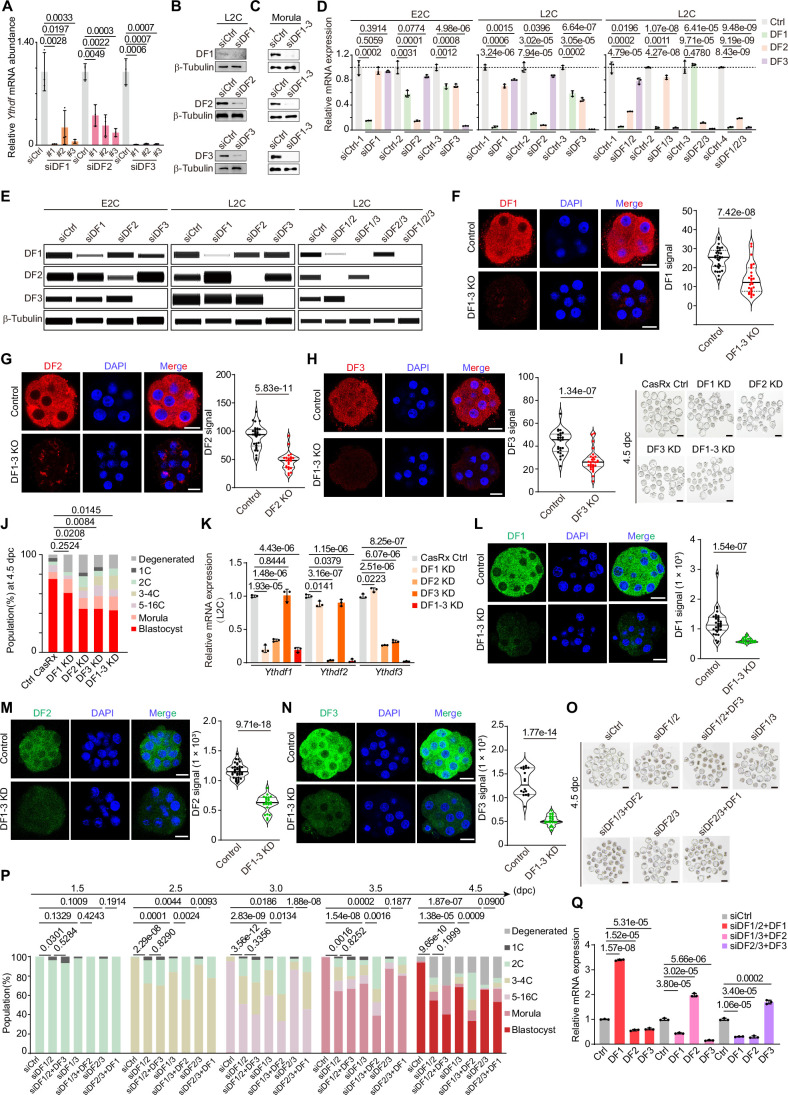
Associated with Fig. 1. (**A**) RT-qPCR validation of *Ythdf* knockdown in L2C embryos. #1#2#3, three different *Ythdf* siRNAs. Data are mean ± SD, *n* = 3 biological replicates. (**B**, **C**) Western blotting showing the knockdown efficiency of *Ythdf* in L2C (**B**) and morula (**C**) embryos. (**D**) Relative expression of *Ythdf1/2/3* measured by RT-qPCR following single, double, or triple DF knockdown in E2C and L2C embryos. Data are mean ± SD, *n* = 3 biological replicates. (**E**) Simple Western immunoblotting assays showing DF1/2/3 protein levels when single DF, double DF, or triple DF knockdown in the E2C and L2C embryos. (**F**–**H**) Representative images of immunofluorescence staining of DF1 (**F**), DF2 (**G**), and DF3 (**H**) with DAPI counterstain in Cas9 control and DF1-3 KO morula at 3.5 dpc. Scale bars, 20 µm. Violin plots showing the DF1 (Control, *n* = 31; DF1-3 KO, *n* = 27), DF2 (Control, *n* = 30; DF1-3 KO, *n* = 23), and DF3 (Control, *n* = 25; DF1-3 KO, *n* = 29) intensity in Cas9 control and DF1-3 KO morula at 3.5 dpc. The upper and lower dotted lines in the violin plots represent upper and lower quartiles (25th and 75th percentiles), and the center line represents the median. (**I**) Representative images showing embryos treated with *Ythdf* guide RNAs (CasRx) or control guide RNA at 4.5 dpc. Representative images were selected from two independent experiments. Scale bars, 100 μm. (**J**) Percentages of embryonic stages observed at the 4.5 dpc in CasRx Control (CasRx Ctrl, *n* = 28), DF1 KD (*n* = 28), DF2 KD (*n* = 27), DF3 KD (*n* = 54), and DF1-3 KD (*n* = 28) groups. (**K**) Relative expression of *Ythdf1/2/3* measured by RT-qPCR following single, double, or triple DF knockdown in L2C embryos. Data are mean ± SD, *n* = 3 biological replicates. (**L**–**N**) Representative images of immunofluorescence staining of DF1 (**L**), DF2 (**M**), and DF3 (**N**) with DAPI counterstain in CasRx control and DF1-3 KD morula at 3.5 dpc. Scale bars, 20 µm. Violin plots showing the DF1 (Control, *n* = 33; DF1-3 KD, *n* = 21), DF2 (Control, *n* = 30; DF1-3 KD, *n* = 17), and DF3 (Control, *n* = 18; DF1-3 KD, *n* = 21) intensity in CasRx control and DF1-3 KD morula at 3.5 dpc. The upper and lower dotted lines in the violin plots represent upper and lower quartiles (25th and 75th percentiles), and the center line represents the median. (**O**) Representative images showing embryos treated with *Ythdf* siRNAs, *Ythdf* mRNA, or control siRNA at 4.5 dpc. Representative images were selected from 2 to 4 independent experiments. Scale bars, 100 μm. (**P**) Percentages of embryonic stages observed at the indicated time points in siCtrl (*n* = 128), siDF1/2 (*n* = 55), siDF1/2 + DF3 (*n* = 30), siDF1/3 (*n* = 57), siDF1/3 + DF2 (*n* = 36), siDF2/3 (*n* = 96), and siDF2/3 + DF1(*n* = 81) conditions. *P* values in (**J**, **P**) were determined by the Chi-square test. (**Q**) RT-qPCR validation of *Ythdf* mRNA in blastocysts with double *Ythdf* knockdown and another *Ythdf* overexpression. Data in (**A**, **E**) are mean ± SD, *n* = 3 biological replicates. The *P* value in (**A**, **D**, **F**, **G**, **H**, **K**, **L**, **M**, **N**, **Q**) was determined by a two-tailed unpaired *t* test. Source data are available online for this figure.

To investigate the impact of perturbing *Ythdf* expression on development, we employed short interfering RNAs (siRNAs) to specifically target endogenous *Ythdf1*, *Ythdf2*, and *Ythdf3*, as well as to deplete all three *Ythdf* transcripts simultaneously. We first confirmed that *Ythdf* siRNAs effectively and specifically knock down *Ythdf1*, *Ythdf2*, and *Ythdf3* expression at both the RNA and protein levels from the L2C stage onwards (Fig. EV1A–C; Appendix Fig. S1). We then monitored the developmental effects of knocking down *Ythdf1*, *Ythdf2*, and *Ythdf3* individually, or all three in combination, on early embryogenesis. This was achieved by microinjecting the corresponding siRNA into two-pronuclear (PN2) zygotes and culturing the embryos in vitro until the blastocyst stage (Fig. 1C). Embryos with triple knockdown of *Ythdf1-3* exhibited a significant developmental delay starting from 2.5 days post-coitus (dpc), resulting in a notable decrease in blastocyst formation (Fig. 1D,E). The embryos appear to develop normally through the 2C stage, successfully undergoing the crucial maternal-to-zygotic transition (MZT), irrespective of individual or combined knockdown of *Ythdf1-3*. This contrasts with reports that conditional knockout (KO) of *Ythdf2* leads to embryo arrest primarily at the 2C stage (Ivanova et al, 2017). We reasoned that DF proteins might still be present before the L2C stage when embryos are treated with DF siRNAs at the PN2 stage, allowing the development of knockdown embryos beyond the 2C stage. Indeed, Immunofluorescence and Western blotting assays showed that DF1 and DF2 proteins remain detectable at the E2C stage despite the dramatic decrease in *Ythdf1* and *Ythdf2* RNA levels when using siRNAs to deplete these transcripts (Fig. EV1D,E; Appendix Fig. S1). Of note, knockdown of *Ythdf2* or *Ythdf3* alone led to a modest yet significant reduction in blastocyst formation, whereas knockdown of Ythdf1 had minimal effects on early embryonic development (Fig. 1D,E). In addition, we observed another abnormal phenotype in *Ythdf* triple knockdown embryos: a notable increase in the number of blastomeres per embryo at 3.0 and 3.5 dpc (Fig. 1F,G).

To rule out potential off-target effects associated with siRNA-mediated *Ythdf* knockdown, we employed both the CRISPR/Cas9 and CasRx systems (Guo et al, 2021; Zuo et al, 2017) to individually or combinatorially knock out or knock down *Ythdfs* in early embryos. Following microinjection, we confirmed that knockout or knockdown of *Ythdf2* or *Ythdf3* alone, or all three *Ythdfs* together, significantly impaired blastocyst formation. In contrast, *Ythdf1* knockout or knockdown had minimal effects on early embryonic development (Figs. 1H–J and EV1F–N). Additionally, we observed a notable increase in the number of blastomeres per embryo at 3.0 dpc in DF1-3 KO embryos (Fig.1K). These findings are consistent with those observed in siRNA-mediated *Ythdf* knockdown experiments, reinforcing the conclusion that the phenotypes are specific and not artifacts of the siRNA-mediated knockdown method.

A previous study demonstrated that knockdown of any individual DF protein in HeLa cells induced compensatory upregulation of the remaining DF paralogs, potentially masking the phenotypic consequences of single or double knockdowns (Zaccara and Jaffrey, 2020). To investigate whether similar compensatory effects underlie the phenotypic differences observed in our *Ythdf* knockdown embryos, we analyzed the expression of each DF at both the RNA and protein levels following single and double DF knockdowns. Our findings reveal that compensatory effects among *Ythdf*s do not occur at the RNA level before the L2C stage (Fig. EV1D). However, at the protein level, DF2 and DF3 can compensate for the loss of DF1 or DF1/2 in L2C embryos. In contrast, DF1 does not compensate for the loss of DF2, DF3, or DF2/3, consistent with the developmental defects observed in si*Ythdf2* or si*Ythdf3* knockdowns but not in si*Ythdf1* knockdowns (Fig. EV1E). Moreover, simultaneous knockdown of any two *Ythdf* members resulted in significant developmental delays, beginning at 2.5 dpc (Fig. EV1O–Q). Unexpectedly, these defects were exacerbated rather than rescued by overexpressing an additional DF protein in other *Ythdf* double-knockdown embryos, suggesting that compensatory mechanisms among DF proteins are insufficient to restore normal development. Notably, intrinsic properties of DF proteins, such as their ability to undergo liquid-liquid phase separation (LLPS), can drive the formation of endogenous LLPS compartments that support compartment-specific mRNA regulation (Fu and Zhuang, 2020; Gao et al, 2019; Ries et al, 2019; Wang et al, 2019). In parallel, post-translational modifications (PTMs), such as glycosylation, modulate protein localization, ligand or partner interactions, and stress granule (SG) assembly (Chen et al, 2023; Sikorski et al, 2023), ultimately shaping the compensatory efficiency of DFs. Together, these findings suggest that DF1, 2, and 3 act in a coordinated manner during early embryogenesis, with each member contributing to developmental progression to varying degrees.

### DF1/2/3 control maternal transcript clearance and 2 C gene expression

**Figure 2:**
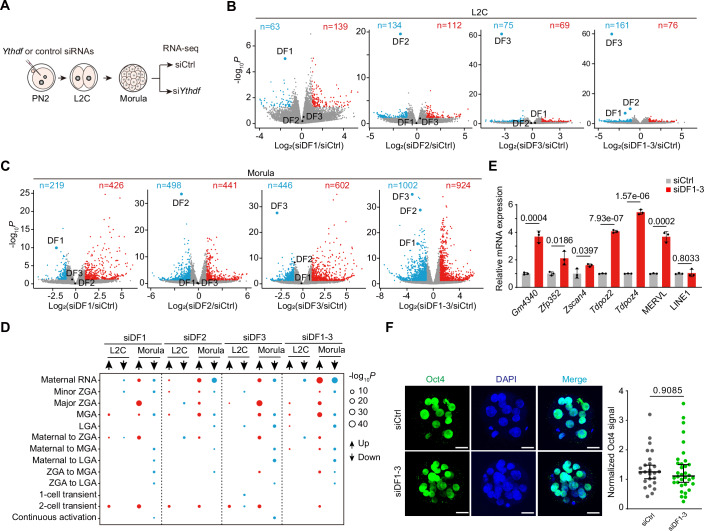
Transcriptomic effects of si*Ythdf* knockdown. (**A**) Schematic of the experimental procedure for performing RNA-seq at L2C and morula. (**B**, **C**) Volcano plots showing gene expression changes upon si*Ythdf* knockdown at the L2C (**B**) and morula (**C**) stages (3 biological replicates for each knockdown condition). The *P* value was determined using DESeq2 (Love et al, 2014), with a threshold of 0.05. (**D**) Bubble plot showing the overlaps between all DEGs in *Ythdf* knockdown embryos and the expression patterns of the developmental transcriptome, based on datasets from previous publications (Deng et al, 2014). The bubble plot sizes show the −log_10_[*P* values] derived from Fisher’s exact test. (**E**) RT-qPCR validation of upregulated 2C genes in si*Ythdf* knockdown morula embryos. Data are mean ± SD, *n* = 3 biological replicates. The *P* value was determined by a two-tailed unpaired *t* test. (**F**) Representative images of immunofluorescence staining of OCT4 with DAPI (4,6-diamidino-2-phenylindole) counterstain in control and si*Ythdf1-3* knockdown morula at 3.5 dpc. Scale bars, 20 µm. Dot plots showing the relative intensity for OCT4 in control (*n* = 25) and si*Ythdf1-3* knockdown morula (*n* = 34) at 3.5 dpc, normalized to the DAPI signal. The error bar represents the mean ± SD. *P* value was determined by a two-tailed unpaired *t* test. Source data are available online for this figure.

To investigate the impacts of DFs on gene expression profiles during embryonic development, we conducted RNA-seq analysis on si*Ythdf* knockdown and control embryos at the L2C and morula stages (Fig. 2A). As expected, knockdown of *Ythdf1*, *Ythdf2*, *Ythdf3*, or all three *Ythdf1-3* in morula-stage embryos resulted in a substantial number of differentially expressed genes (DEGs). In contrast, at the L2C stage, only mild changes in gene expression were observed (Fig. 2B,C; Dataset EV1), which is consistent with the observation that embryos develop normally through the 2-cell stage despite si*Ythdf* knockdown. Notably, RNA-seq analysis confirmed that each *Ythdf* siRNA effectively and specifically depleted its target transcript, as shown in Appendix Fig. S2A,B. Importantly, the targeted *Ythdf* depletion does not affect the expression of the other two *Ythdf* members, further confirming that compensatory effects among DFs do not boost at the RNA level. Additionally, the knockdown of *Ythdf1-3* results in a greater number of DEGs compared to single *Ythdf* knockdown embryos at the morula stage (Fig. 2C), which correlates with the most severe embryonic phenotype observed in si*Ythdf* triple knockdown, marked by the lowest blastocyst development rate (36%). In contrast, at the L2C stage, there is a small amount of increased DEGs in si*Ythdf1-3* knockdown embryos compared to single *Ythdf* knockdown embryos. This suggests that knockdown of any DF protein has minimal impact on the 2C program, thus supporting our results that residual DF proteins before the L2C stage allow knockdown embryos to develop beyond the 2C stage.

Subsequently, we categorized the early embryonic-expressed genes into 13 clusters based on their expression patterns, including maternal RNA, minor ZGA, major ZGA, mid-preimplantation gene activation (MGA, with expression peaking at the 4–8 cell stage), late-preimplantation gene activation (LGA, with expression peaking at the morula or blastocyst stage), and others (Appendix Fig. S2C; Dataset EV2), using publicly available datasets of the dynamic transcriptome during early development (Wang and Dey, 2006). Among the detected DEGs from si*Ythdf* knockdown embryos, a great number were associated with the clusters of maternal RNA, major ZGA, MGA, and 2-cell transient (Fig. 2D), suggesting that si*Ythdf* knockdown disrupts the normal expression of zygotic and mid-preimplantation activated genes, as well as the clearance of maternal mRNA, contributing to developmental defects. In particular, the upregulated genes in different DF-deficient morulae exhibit similar expression patterns, while the number of unique DEGs identified in each si*Ythdf* knockdown embryo increases from the L2C to the morula stage (Appendix Fig. S2D,E), reflecting the extent of developmental defects associated with individual DF knockdowns. Interestingly, the upregulated genes in the si*Ythdf1-3* triple knockdown morulae include well-known markers of 2C genes, such as *Gm4340*, *Zscan4*, *Zfp352*, and *Tdpoz2/4*, along with the 2C-specific transposon MERVL (murine endogenous retrovirus-L) (Macfarlan et al, 2012) (Fig. 2E; Appendix Fig. S2F). Consistently, both Cas9-mediated DF1-3 KO and CasRx-mediated DF1-3 KD blastocysts exhibited upregulation of 2C genes and MERVL (Appendix Fig. S2G,H). This likely reflects a delayed clearance of 2C-stage transcripts, suggesting that DF1-3 triple-deficient embryos retain a 2C/totipotent-like transcriptomic profile even at the LGA stage. In addition, double knockdown of any two *Ythdf* members also results in varying degrees of upregulation of 2C genes, comparable to triple knockdown of *Ythdf1-3* (Fig. 2E; Appendix Fig. S2I). Consistently, single si*Ythdf* knockdowns lead to a reduction in both the number and expression levels of the 2C genes upregulated in si*Ythdf1-3* triple knockdown morulae (Appendix Fig. S2J,K), suggesting that the increase in 2C gene transcripts correlates positively with the severity of the developmental phenotype observed in si*Ythdf* knockdown embryos.

Next, we were interested in exploring the potential impact of DF proteins on early cell lineage specification during preimplantation development, considering the report that m^6^A-marked mRNA facilitates the process of naïve pluripotency toward differentiation from post-implantation E5.5 embryos (Geula et al, 2015). To address this, we examined mRNA and protein levels of genes related to the inner cell mass (ICM), such as *Oct4* (also known as *Pou5f1*) and *Nanog*, as well as trophectoderm differentiation-related gene (*Cdx2*), in *Ythdf1-3* triple knockdown embryos at 3.5 dpc. As expected, there is no significant difference in the mRNA and protein levels of Oct4, Nanog, and Cdx2 between si*Ythdf*1-3 KD embryos and the control group (Fig. 2F; Appendix Fig. S2L–O), suggesting that DF proteins do not play a major role in early embryonic lineage differentiation. This observation is consistent with previous reports showing that Mettl3 KO blastocysts at E3.5 exhibit normal morphology and expression of pluripotency markers, and that *Ythdf* triple KO embryos develop normally until E7.5 but fail to progress beyond that stage (Geula et al, 2015; Lasman et al, 2020). Gene ontology analysis of the DEGs identified from the si*Ythdf1-3* knockdown morula shows that these genes are predominantly involved in crucial pathways, including regulation of cell adhesion, phosphate metabolic process, and cell development (Appendix Fig. S2P,Q).

### Phenotypes observed in *Ythdf1-3* knockdown embryos are associated with m^6^A marks

**Figure EV2:**
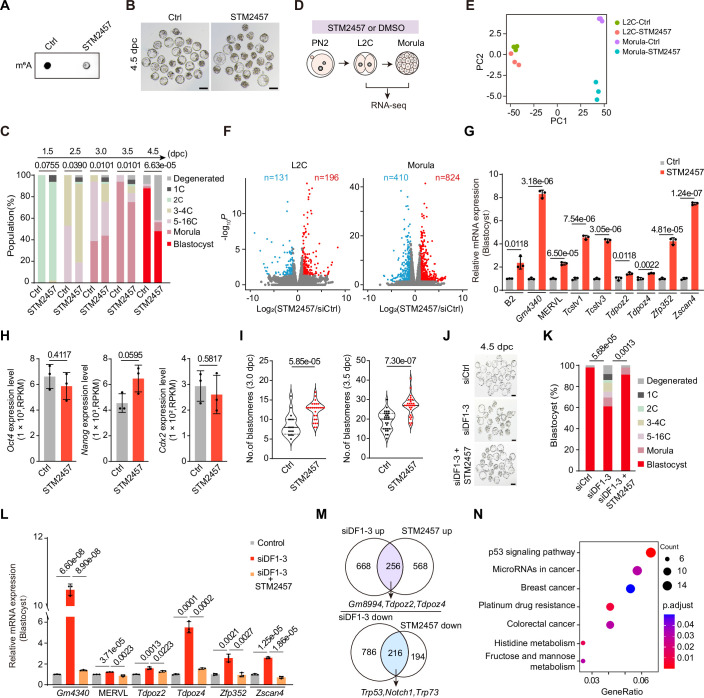
Associated with Fig. 1. (**A**) Dot blotting showing the m^6^A level of total RNAs in control and STM2457-treated morula embryos. (**B**) Representative images showing embryos treated with STM2457 or DMSO control at 4.5 dpc. Scale bars, 100 μm. (**C**) Percentages of embryonic stages observed at the indicated time points in control (*n* = 49) and STM2457-treated (*n* = 48) groups. *P* values were determined by the Chi-square test. (**D**) Schematic of the experimental procedure for detecting the transcriptome changes of STM2457-treated embryos at the L2C and morula stages. (**E**) PCA showing STM2457-treated and control samples at the L2C and morula stages. (**F**) Volcano plots showing gene expression changes upon STM2457-treated embryos at the L2C (left) and morula (right) stages (3 biological replicates for each knockdown condition). The *P* value was determined using DESeq2 (Love et al, 2014), with a threshold of 0.05. (**G**) Relative 2C gene and B2 RNA expression measured by RT-qPCR at the blastocyst stage. Data are mean ± SD, *n* = 3 biological replicates. (**H**) Bar chart showing gene expression from STM2457-treated and control RNA-seq data at the morula stage. Data are mean ± SD, *n* = 3 biological replicates. (**I**) Violin plot showing the total number of blastomeres per embryo in control and STM2457-treated morulae at 3.0 (Ctrl, *n* = 16; STM2457, *n* = 27) and 3.5 (Ctrl, *n* = 28; STM2457, *n* = 29) dpc. The upper and lower dotted lines in the violin plots represent upper and lower quartiles (25th and 75th percentiles), and the center line represents the median. (**J**) Representative images showing siDF1-3 KD embryos rescued with STM2457 treatment at 4.5 dpc. Scale bars, 100 μm. (**K**) Percentages of embryonic stages observed at the 4.5 dpc in Control (*n* = 47), siDF1-3 (*n* = 36), and siDF1-3 + STM2457 (*n* = 45) groups. *P* values were determined by the Chi-square test. (**L**) RT-qPCR validation of 2C genes in Control, siDF1-3, and siDF1-3 + STM2457 treated embryos at 4.5 dpc. Data are mean ± SD, *n* = 3 biological replicates. *P* values in (**G**–**I**, **L**) were determined by a two-tailed unpaired *t* test. (**M**) Venn diagram showing the overlap of upregulated (upper) and downregulated (bottom) genes identified between si*Ythdf1-3* and STM2457-treated RNA-seq data at the morula stage. (**N**) GO analysis of common DEGs identified in both si*Ythdf1-3* knockdown and STM2457-treated morula embryos. The *P* value was determined by Fisher’s exact test. Source data are available online for this figure.

Considering that DF proteins regulate transcripts by recognizing the m^6^A marks on RNAs, we sought to investigate whether the phenotype caused by si*Ythdf* knockdown is associated with the m^6^A marks. To this end, we employed a METTL3 inhibitor (STM2457) to treat embryos (Wu et al, 2022), which greatly reduced m^6^A levels and blastocyst formation (Fig. EV2A–C). Next, we conducted RNA-seq analysis on STM2457-treated and control embryos at the L2C and morula stages (Fig. EV2D–F). Specifically, STM2457-treated embryos exhibited marked upregulation of 2C genes and MERVL (Fig. EV2G). Of note, the expression of pluripotency and differentiation-associated genes remained unchanged, in line with the report that Mettl3 KO blastocysts at E3.5 display normal morphology and expression of pluripotency markers and do not exhibit defects in early embryonic lineage differentiation (Geula et al, 2015) (Fig. EV2H; Dataset EV3). Unexpectedly, there was a notable increase in blastomere number per embryo at 3.0 and 3.5 dpc (Fig. EV2I), consistent with the characteristics observed in si*Ythdf1-3* knockdown embryos. Moreover, the development defects resulting from *Ythdf1-3* KD can be largely rescued by STM2457 treatment (Fig. EV2J–L). These findings indicate that the phenotype of si*Ythdf1-3* knockdown is dependent on m^6^A marks. We further analyzed the overlap of DEGs between si*Ythdf1-3* knockdown and STM2457-treated embryos. The shared upregulated genes include well-known 2C markers such as *Gm8994*, *Tdpoz2*, and *Tdpoz4*, whereas the shared downregulated genes, including *Trp53*, *Notch1*, and *Trp73* (Fig. EV2M), are enriched in the p53 signaling pathway (Fig. EV2N). These findings suggest a potential mechanism by which *Ythdf1-3* knockdown promotes enhanced cell proliferation and accelerated blastomere division in early embryos.

### DF1/2/3 proteins bind similar m^6^A sites

**Figure 3:**
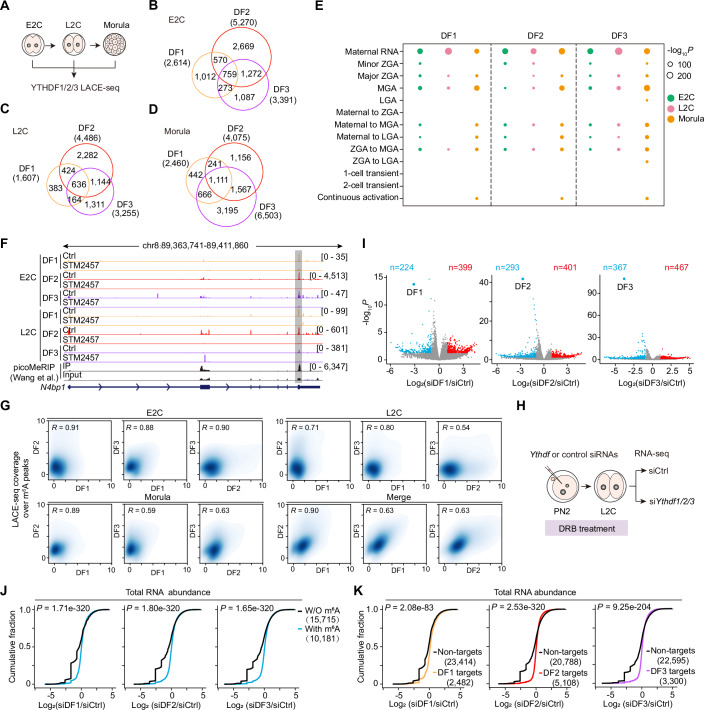
DF1/2/3 proteins promote transcript decay. (**A**) Schematic of the experimental procedure for performing DF1/2/3 LACE-seq in the E2C, L2C, and morula embryos. (**B**–**D**) Venn diagram showing the overlap of target genes identified in DF1/2/3 LACE-seq replicates at the E2C (**B**), L2C (**C**), and morula (**D**) stages. (**E**) Bubble plot showing the overlap between DF-binding genes and the expression patterns of the developmental transcriptome, based on datasets from previous publications (Deng et al, 2014). The bubble plot sizes show the −log10[*P* values] derived from Fisher’s exact test. (**F**) Genome browser snapshot showing the read coverage of *N4bp1* in DF1/2/3 LACE-seq datasets and the picoMeRIP-seq dataset (Wang et al, 2023). (**G**) Density plots showing the pairwise comparison of normalized Ythdf1/2/3 binding signals (LACE-seq reads) surrounding individual m^6^A peaks (picoMeRIP-seq datasets from 2C and 8C embryos) across the E2C, L2C, morula, and combined developmental stages. The DF1/2/3 LACE-seq datasets from E2C and L2C embryos were referenced against the m^6^A peaks identified in the 2C picoMeRIP-seq datasets; the DF1/2/3 LACE-seq datasets from morula embryos were referenced against the m^6^A peaks identified in the 8C picoMeRIP-seq datasets. The combined DF1/2/3 LACE-seq datasets from E2C, L2C, and morula embryos were compared with the combined m^6^A peaks from the 2C and 8C picoMeRIP-seq datasets. (**H**) Schematic of the experimental procedure for detecting the transcriptome stability of DF-depleted embryos treated with DRB (5,6-dichloro-1-β-d-ribofuranosylbenzimidazole). (**I**) Volcano plot showing changes in transcript stability upon knockdown of *Ythdf* expression in DRB-treated embryos at the L2C stage (3 biological replicates for each knockdown condition). The *P* value was determined using DESeq2(Love et al, 2014), with a threshold of 0.05. (**J**) Cumulative distributions of the log_2_ fold changes in RNA abundance between si*Ythdf* and siControl for m^6^A-modified and unmodified RNAs in DRB-treated L2C embryos, m^6^A-modified datasets from previous publications (Wang et al, 2023). *P* values were determined by the Kolmogorov–Smirnov test. (**K**) Cumulative distributions of the log_2_ fold changes in RNA abundance between si*Ythdf* and siControl for non-targets and DF LACE-seq targets at the E2C stages. *P* values were determined by the Kolmogorov–Smirnov test.

**Figure EV3:**
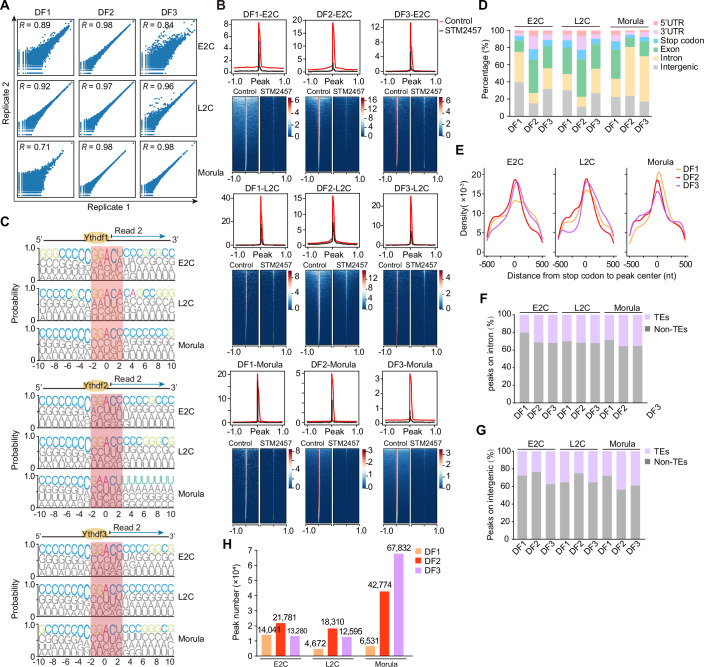
Associated with Fig. 3. (**A**) The correlations of DF1/2/3 LACE-seq between two biological replicates. *R* refers to Pearson’s correlation coefficient. (**B**) Density plots and heatmaps showing the distribution of peak centers within a 1 kb window in the genome for DF1/2/3 LACE-seq data at the E2C, L2C, and morula stages. (**C**) WebLogo showing the base frequency at and around the DF-RNA crosslinking sites. (**D**) Bar chart showing the distribution of DF1/2/3 binding peaks in genomic regions at the E2C, L2C, and morula stages. (**E**) Density plots showing the distribution of peak centers within a 500-nt window in the genome flanking the mRNA stop codon for DF1/2/3 LACE-seq data at the E2C, L2C, and morula stages. (**F**, **G**) Bar chart showing the distribution of DF binding peaks upon the intron (**F**) and intergenic (**G**) region at the E2C, L2C, and morula stages. (**H**) Bar plots summarizing the number of DF1/2/3 binding peaks in the E2C, L2C, and morula embryos. Source data are available online for this figure.

Although the precise roles of DF proteins are still under discussion in various cell lines and organisms, we aim to identify the binding targets and potential roles of DF1/2/3 proteins during early development. To address this, we utilized the LACE-seq method (Su et al, 2021), previously developed by our group for efficient capture of RNA-binding protein targets from limited starting materials, to investigate the binding profiles of DF1, DF2, and DF3 in E2C, L2C, and morula embryos (Figs. 3A and EV3A). To ensure rigorous control over our findings, we conducted DF1/2/3 LACE-seq to explore their binding profiles in both control and STM2457-treated embryos across these three early developmental stages (Table EV2). The binding of DF1/2/3 is dramatically decreased in STM2457-treated embryos, characterized by a preference for binding the m^6^A classic motif DRACH (D = G/A/T, R = A/G, H = A/T/C) (Fig. EV3B,C), indicating that the binding of DF1/2/3 is dependent on m^6^A modification.

Next, we utilized DF1/2/3 LACE-seq datasets from STM2457-treated embryos as negative controls to mitigate potential background signals in DF1/2/3 binding observed in corresponding control embryos. Furthermore, to identify high-confidence m^6^A-dependent DF targets for downstream functional characterization, we rigorously filtered DF binding peaks for the presence of the canonical m^6^A consensus motif. After filtering out noise signals, we observed a dynamic distribution of DF1/2/3-bound peaks across the genome, with enrichment around mRNA stop codons observed for all three m^6^A readers across all stages (Fig. EV3D,E), consistent with the binding features of DF proteins in cell lines (Patil et al, 2016; Shi et al, 2017; Wang et al, 2014; Wang et al, 2015). It is noteworthy that the DF1/2/3 LACE-seq libraries were generated using total RNA from cell lysates, thereby enabling the detection of DF1/2/3 binding events across exonic, intronic, and intergenic regions. This is consistent with previous findings showing that total RNA samples, as opposed to poly(A)⁺ RNA, contain a greater proportion of m^6^A sites within promoter, intronic, and intergenic regions (Linder et al, 2015). A major contributor to the observed DF1/2/3 enrichment in intronic and intergenic regions is the abundant expression of retrotransposon RNAs; notably, 20%–44% of DF1/2/3 binding peaks are associated with transposable elements (TEs) situated within intronic and intergenic regions (Fig. EV3F,G). We identified dynamic binding peaks of DF1/2/3 proteins during early development (Fig. EV3H; Dataset EV4), characterized by an increase in DF2/3 binding peaks and a concomitant decrease in DF1 binding peaks from the E2C to morula stages.

At the non-intronic gene level, we observed a reciprocal overlap of DF targets between two DF proteins, ranging from 23.6 to 72.2% (Fig. 3B–D). Among the DF-bound genes in 2C and morula embryos, a great number are associated with maternal RNA and MGA genes (Fig. 3E). This suggests that DF proteins predominantly regulate maternal mRNA and MGA genes, rather than ZGA genes, even though numerous major ZGA genes are significantly upregulated in DF-deficient morulae (Fig. 2D). Furthermore, we confirmed a substantial overlap of peaks and transcripts between DF-bound coding RNAs and m^6^A-occupied coding RNAs in 2C embryos (Wang et al, 2023) (Appendix Fig. S3A,B), implying that the DF binding to transcripts is closely associated with m^6^A modification, as exemplified by the m^6^A-modified regions of *N4bp1* in 2C embryos (Fig. 3F).

To explore whether DF1/2/3 proteins bind to overlapping m^6^A sites, we compared DF1/2/3 LACE-seq binding signals at m^6^A peaks identified from the picoMeRIP-seq datasets (Wang et al, 2023). DF proteins exhibited a high degree of correlation with each other over m^6^A peaks at individual stages (E2C, L2C, or Morula) or across overall early developmental stages (combined DF LACE-seq data from the E2C, L2C, and morula stages), with Pearson correlation coefficients ranging from *R* = 0.54 to 0.91 (Fig. 3G), suggesting that DF1/2/3 largely bind to overlapping sets of m^6^A sites during early embryogenesis. Collectively, these results suggest that DF proteins recognize similar m^6^A sites at each stage of early embryonic development.

### DF proteins promote targeted mRNA decay

Given the ongoing debate regarding the cellular functions of DF1/2/3 proteins, particularly whether DF1, like DF2 and DF3, contributes to transcript decay, we investigated whether these DF proteins mediate targeted mRNA degradation to regulate transcript decay during early embryonic development, with a specific focus on DF1. To achieve this, we conducted RNA-seq on L2C embryos with *Ythdf1*, *Ythdf2*, or *Ythdf3* knockdown, respectively, accompanied by 5,6-dichloro-1-β-d-ribofuranosylbenzimidazole (DRB) treatment of these knockdown and control embryos from the PN2 one-cell stage, which inhibits RNA polymerase II (Pol II) transcription elongation during minor and major ZGA (Abe et al, 2018) (Fig. 3H; Appendix Fig. S3C). As expected, the knockdown of *Ythdf1*, *Ythdf2*, and *Ythdf3* in DRB-treated embryos led to a greater number of DEGs compared to embryos where *Ythdf* knockdown occurred without DRB treatment, particularly among the subsets of upregulated genes (Figs. 2B and 3I; Appendix Fig. S3D; Dataset EV5), indicating that DRB treatment effectively mitigates the interference of Pol II transcription in DF-mediated RNA decay. Consequently, a cluster of genes shows extended lifespan in si*Ythdf* knockdown embryos treated with DRB. Thus, these data strongly indicate that each DF protein promotes mRNA decay at the 2C stage, with individual DF proteins regulating partially distinct subsets of target genes (Appendix Fig. S3E).

Then, we categorized the evaluated transcripts based on m^6^A peaks inferred from the picoMeRIP-seq dataset (Wang et al, 2023) in 2C embryos into two groups: m^6^A-unmodified and m^6^A-modified transcripts. Compared to m^6^A-unmodified transcripts, a noticeable increase in the abundance of m^6^A-modified transcripts was observed in si*Ythdf1*, si*Ythdf2*, and si*Ythdf3* embryos subjected to DRB treatment, respectively (Fig. 3J), suggesting that DF1, similar to DF2 and DF3, plays a role in facilitating the decay of m^6^A-modified transcripts. Subsequently, we classified 2C transcripts into non-target RNAs and mRNAs occupied by DF1, DF2, or DF3 based on binding peaks defined by DF1/2/3 LACE-seq datasets from E2C and L2C embryos, respectively. Compared to corresponding non-target RNAs, dramatic increases in mRNA stabilization of DF1/2/3-binding RNAs were observed in si*Ythdf1*, si*Ythdf2*, and si*Ythdf3* embryos following DRB treatment, respectively (Fig. 3K; Appendix Fig. S3F), suggesting that DF1, along with DF2 and DF3, facilitates the decay of its target transcripts by recognizing m^6^A-modified sites. Conservation analysis revealed that the mouse DF1 protein shows higher similarity to mouse DF2 and human DF2 proteins, rather than human DF1 protein, despite the similarity calculations between two mouse DF proteins falling within the range of 65.87–68.37% (Appendix Fig. S3G,H), indicating that the function of the mouse DF1 protein is more akin to mouse DF2 and human DF2. Thus, these results demonstrate that DF1/2/3 proteins facilitate RNA decay with binding to m^6^A-marked transcripts in the 2C embryos.

### DF1/2/3 proteins promote B2 RNA decay

**Figure 4:**
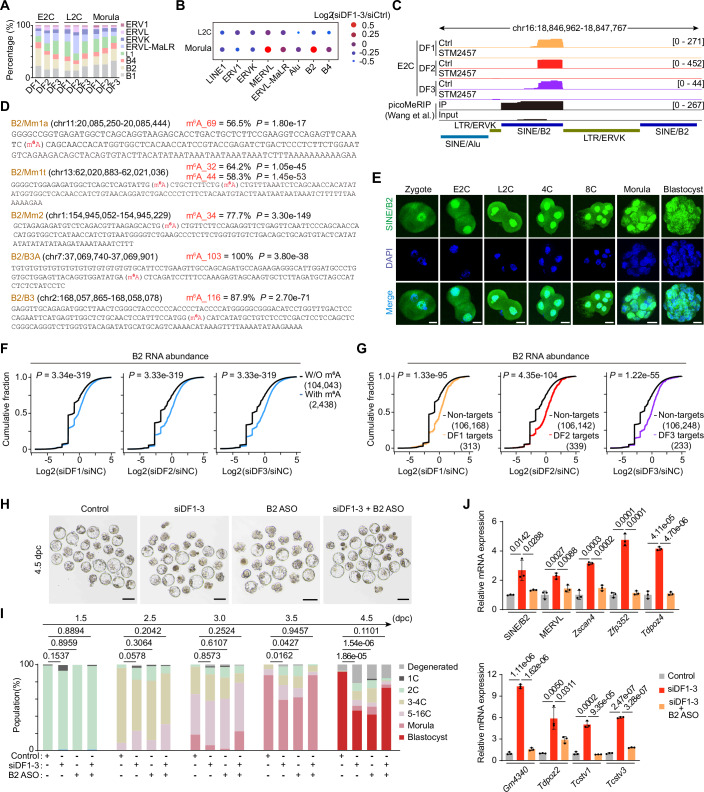
B2 transcripts are major targets of DF1/2/3 proteins. (**A**) Histogram showing the enrichment score of DF-binding reads in major LINE, LTR, and SINE subfamilies at the E2C, L2C, and morula stages. (**B**) Bubble plot showing changes in TE transcript abundance (si*Ythdf1-3*/siControl) at the L2C and morula stages. (**C**) Genome browser snapshot showing the read coverage of the B2 locus in DF1/2/3LACE-seq datasets and the picoMeRIP-seq datasets. (**D**) Alignment of consensus sequences from B2 subfamilies, with annotated putative m^6^A sites and quantification of their methylation stoichiometries derived from published GLORI datasets in mESCs (Liu et al, 2022). *P* values were determined based on the binomial distribution. (**E**) Representative images of RNA-FISH for B2 RNA with DAPI counterstain during preimplantation development. Scale bars, 20 μm. (**F**) Cumulative distribution of the log_2_ fold changes in B2 RNA abundance in DRB-treated L2C embryos between si*Ythdf* and siControl for m^6^A-modified and unmodified RNAs. *P* values were determined with the Kolmogorov–Smirnov test. (**G**) Cumulative distribution of the log_2_ fold changes in B2 RNA abundance in DRB-treated L2C embryos between si*Ythdf* and siControl for non-targets and DF LACE-seq targets. *P* values were determined with the Kolmogorov–Smirnov test. (**H**) Representative images showing embryos treated with *Ythdf* siRNAs, B2 ASO, *Ythdf* siRNAs + B2 ASO, or control siRNA at 4.5 dpc. Representative images were selected from two independent experiments. Scale bars, 100 μm. (**I**) Percentages of embryonic stages observed at the indicated time points in Control (*n* = 42), B2 ASO (*n* = 35), siDF1-3 (*n* = 52), and siDF1-3 + B2 ASO (*n* = 51) groups. *P* values were determined by the Chi-square test. (**J**) RT-qPCR validation of B2 transcripts and 2C genes in Control, siDF1-3, and siDF1-3 + B2 ASO-treated embryos at 4.5 dpc. Data are mean ± SD, *n* = 3 biological replicates. *P* values were determined by a two-tailed unpaired *t* test. Source data are available online for this figure.

**Figure EV4:**
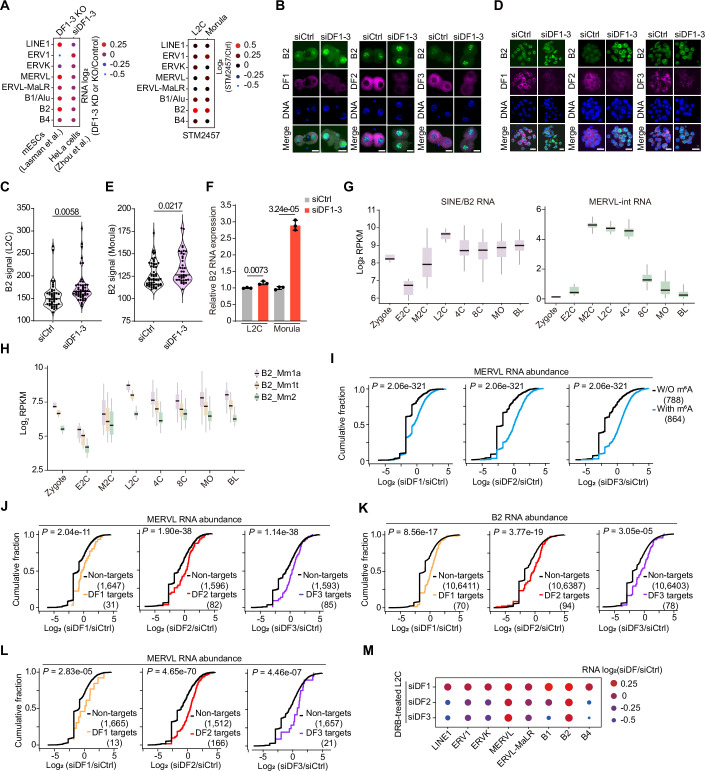
Associated with Fig. 4. (**A**) Bubble plot showing changes in TE transcript abundance in siDF1-3 KD HeLa cells, DF1-3 KO mESCs, and STM2457-treated L2C and morula embryos. (**B**) Representative images of IF/RNA-FISH for B2 RNA with DF counterstain in control and si*Ythdf1-3* knockdown embryos at the L2C stage. Scale bars, 20 µm. (**C**) Violin plot showing the intensity of the B2 signal in control (*n* = 45) and si*Ythdf1-3* knockdown embryos (*n* = 53) at the L2C stage. The upper and lower dotted lines in the violin plots represent upper and lower quartiles (25th and 75th percentiles), and the center line represents the median. (**D**) Representative images of IF/RNA-FISH for B2 RNA with DF counterstain in control and si*Ythdf*1-3 knockdown embryos at the morula stage. Scale bars, 20 µm. (**E**) Violin plot showing the intensity of the B2 signal in control (*n* = 55) and si*Ythdf1-3* knockdown embryos (*n* = 39) at the morula stage. The upper and lower dotted lines in the violin plots represent upper and lower quartiles (25th and 75th percentiles), and the center line represents the median. (**F**) Relative B2 RNA expression measured by RT-qPCR at the L2C and morula stage. Data are mean ± SD, *n* = 3 biological replicates. The *P* values in (**C**, **E**, **F**) were determined by a two-tailed unpaired *t* test. (**G**) Box plots showing expression levels of the B2 transcript (left) and MERVL-int (right) during preimplantation development. Zygote, *n* = 4; Early 2-Cell (E2C), *n* = 8; Middle 2-Cell (M2C), *n* = 12; Late 2-Cell (L2C), *n* = 10; 4-cell (4C), *n* = 14; 8-cell (8C), *n* = 47; Morula (MO), *n* = 58; Blastocyst (BL), *n* = 60. Boxes represent the 25th-75th percentile (line at the median), with whiskers at 1.5× interquartile range (IQR). (**H**) Box plots showing expression levels of B2 subfamilies (B2_Mm1a, B2_Mm1t, and B2_Mm2) during preimplantation development. Zygote, *n* = 4; Early 2-Cell (E2C), *n* = 8; Middle 2-Cell (M2C), *n* = 12; Late 2-Cell (L2C), *n* = 10; 4-cell (4C), *n* = 14; 8-cell (8C), *n* = 47; Morula (MO), *n* = 58; Blastocyst (BL), *n* = 60. Boxes represent the 25th-75th percentile (line at the median), with whiskers at 1.5 × interquartile range (IQR). (**I**) Cumulative distribution of the log_2_ fold changes in MERVL abundance between si*Ythdf* and siControl for m^6^A-modified and unmodified MERVLs in DRB-treated L2C embryos, based on datasets from a previous publication(Wang et al, 2023). *P* values were determined by the Kolmogorov–Smirnov test. (**J**) Cumulative distribution of the log_2_ fold changes in MERVL abundance between si*Ythdf* and siControl for DF1/2/3 LACE-seq targets (E2C) and corresponding non-targets in DRB-treated L2C embryos. *P* values were determined by the Kolmogorov–Smirnov test. (**K**) Cumulative distribution of the log_2_ fold changes in B2 RNA abundance between si*Ythdf* and siControl for DF1/2/3 LACE-seq targets (L2C) and corresponding non-targets in DRB-treated L2C embryos. *P* values were determined by the Kolmogorov–Smirnov test. (**L**) Cumulative distribution of the log_2_ fold changes in MERVL abundance between si*Ythdf* and siControl for DF1/2/3 LACE-seq targets (L2C) and corresponding non-targets in DRB-treated L2C embryos. *P* values were determined by the Kolmogorov–Smirnov test. (**M**) Bubble plot showing changes in TE transcript abundance (si*Ythdf*/siControl) in DRB-treated L2C embryos. Source data are available online for this figure.

During early embryo development, numerous retrotransposons are activated and involved in developmental regulation (Guo et al, 2023; Percharde et al, 2018; Sakashita et al, 2023). We wondered whether DF proteins could directly bind to and regulate the transcripts of active retrotransposons during early embryonic development, as previous reports have shown a high abundance of m^6^A modification on LINE1, LTR, and SINE transcripts (Wang et al, 2023; Wu et al, 2022). We first examined the DF1/2/3 binding on the major subfamilies of transposable elements (TEs) and the expression changes of these TEs in embryos with si*Ythdf1-3* knockdown. Although DF1/2/3 proteins broadly target retrotransposon RNAs, B2 RNAs, as well as the 2C-specific retrotransposon MERVL, were markedly upregulated in si*Ythdf1-3* knockdown morula embryos (Fig. 4A,B). We also observed an increase in B2 RNA levels in DF1-3 KO mESCs (Lasman et al, 2020) and STM2457-treated embryos, but not in DF1-3 KD HeLa cells (Zou et al, 2023) (Fig. EV4A), consistent with the fact that B2 elements are mouse-specific. In contrast, Alu elements are their closest counterparts in humans (Mariner et al, 2008). These findings suggest that DF-mediated regulation of B2 elements may be integral to the mESC and mouse early embryo context. The colocalization of immunofluorescence and fluorescent in situ RNA hybridization (IF/RNA-FISH) and quantitative reverse transcription polymerase chain reaction (RT-qPCR) results confirmed that B2 RNAs are greatly increased in *Ythdf*1-3 knockdown embryos (Fig. EV4B–F). Additionally, the binding signals of each DF protein were well-localized to the m^6^A-marked regions on B2 loci in early embryos (Figs. 4C and EV4B,D). Subclasses of the B2 family, derived from distinct genomic loci, exhibited high levels of m^6^A methylation (Fig. 4D). Furthermore, the consensus sequences surrounding the putative m^6^A sites conformed to the canonical DRACH motif (Fig. 4D), as evidenced by published GLORI datasets from mESCs, supporting the notion that DF proteins target B2 RNAs in an m^6^A-dependent manner.

SINE elements are highly abundant retrotransposons in the mammalian genome, and B2 is a distinct subfamily of SINEs dispersed throughout the rodent genome. its expression is highly observed in germ cells, early embryos, and cultured somatic cells induced by specific cellular stresses (Allen et al, 2004; Bachvarova, 1988; Mangiavacchi et al, 2021). During early embryogenesis, the expression level of B2 RNA declines at the E2C stage and then rapidly increases, remaining at a higher level until the blastocyst stage, distinct from the expression pattern of MERVL (Fig. EV4G), suggesting B2 RNA is rapidly cleared before ZGA, and then reactivated expression along with ZGA occurs. Of note, the B2_Mm1a is the most highly expressed B2 RNA subfamily at each developmental stage (Fig. EV4H), and we designed B2 antisense oligonucleotides (ASOs) targeting B2_Mm1a to knock down B2 RNAs for further study (Table EV1). Then, we observed that B2 RNA exhibited nuclear enrichment with cytoplasmic distribution up to the morula stage and became evenly distributed between the nucleus and cytoplasm at the blastocyst stage (Fig. 4E). These results raise the possibility that nuclear B2 transcripts may play roles as regulators in gene expression and cytosol B2 transcripts may hijack the LINE-encoded proteins to generate and integrate a cDNA copy of the SINE RNA into the genome or influence mRNA translation (Elbarbary et al, 2016).

To shed light on the functional effects of DF proteins on B2 RNAs, we classified B2 transcripts according to their m^6^A modification and DF binding in 2C embryos. As expected, we observed a significant increase in the stabilization of both m^6^A-modified and DF-bound B2 transcripts compared to other B2 transcripts in si*Ythdf* knockdown embryos following DRB treatment (Fig. 4F,G). Accordingly, the increased stabilization of m^6^A-modified and DF-bound MERVL was also observed in the same embryos (Fig. EV4I,J). Likewise, DF proteins also promote B2 RNAs and MERVL decay in L2C embryos (Fig. EV4K,L). Indeed, the abundance of retrotransposon RNAs, including MERVL and B2, is markedly upregulated in DRB-treated embryos with individual si*Ythdf* knockdown (Fig. EV4M), once again confirming that DF1 can promote RNA decay in embryos. Taken together, these findings suggest that DF proteins can mediate the degradation of B2 RNA, along with MERVL, in an m^6^A-dependent manner.

### Depletion of B2 RNA largely rescues the phenotype observed in *Ythdf1-3* knockdown embryos

Given that DF proteins mediate B2 RNA decay in embryos and that B2 RNA is markedly upregulated in si*Ythdf1-3* knockdown morulae, we investigated whether overexpressing B2 RNA could mimic the developmental defects observed with si*Ythdf1-3* knockdown. As expected, embryos microinjected with 1× B2 RNA (without m^6^A modification, ~170 ng/µl) exhibited notable developmental delays beginning at 1.5 dpc and a significant reduction in blastocyst formation (46.6%). In contrast, embryos injected with a lower concentration of B2 RNA showed milder developmental delays starting at 2.5 dpc, suggesting that the phenotype induced by B2 RNA is dose-dependent (Appendix Fig. S4A–C). Next, we conducted RNA-seq analysis on B2 overexpression (OE) and control embryos at the L2C and morula stages (Appendix Fig. S4D–F). B2 OE embryos showed significant downregulation of genes related to ribosome biogenesis and nuclear localization at the L2C and morula stages, respectively (Appendix Fig. S4G; Dataset EV6). Notably, genes upregulated in both B2 overexpression (OE) and *Ythdf1-3* knockdown embryos include well-established 2C markers such as *Gm4340*, *Tdpoz1/4/5*, *Zfp352*, and *Zscan4c*. In contrast, the shared downregulated genes are mainly involved in developmental pathways, including *Creb3l2*, *Cdh3*, *Dhh*, *Fstl3*, *Heg1*, *Notch1*, *Lrp1*, *Plxnb1*, and *Rgs14* (Appendix Fig. S4H). Indeed, we observed significant upregulation of 2C marker genes such as *Gm4340*, *Zscan4*, *Zfp352*, and *Tdpoz2/4*, as well as MERVL, in B2 OE morula embryos (Appendix Fig. S4I,J), resembling the upregulation of these 2C genes in si*Ythdf1-3* knockdown embryos. Similarly, the protein levels of Oct4 and Nanog showed no apparent difference between B2 OE embryos and the control group, despite downregulated mRNA levels of *Oct4* and *Nanog* in B2 OE embryos (Appendix Fig. S4J–L). In addition, there was no observable difference in the number of blastomeres per embryo between B2 OE embryos and the control group at 3.5 dpc (Appendix Fig. S4M). These results indicate that overexpression of B2 RNA partially recapitulates the characteristics observed in *Ythdf1-3* knockdown early embryos, independent of m^6^A-associated effects.

**Figure EV5:**
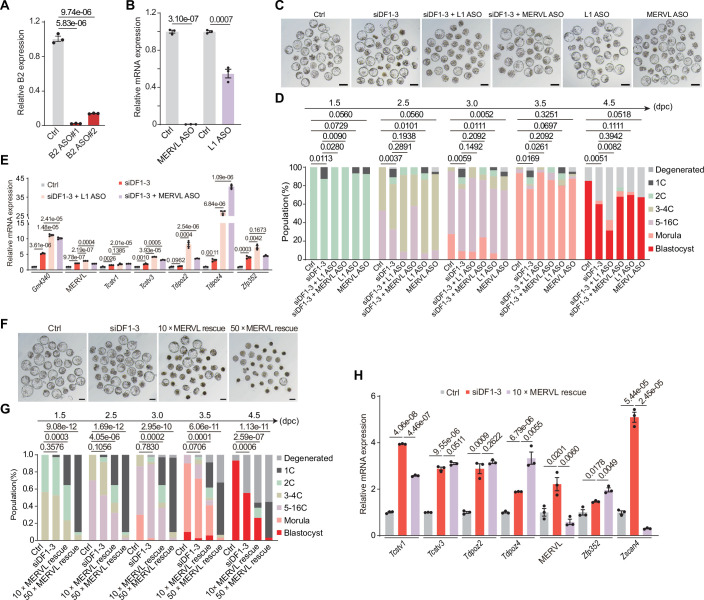
Associated with Fig. 4. (**A**) RT-qPCR validation of B2 knockdown in L2C embryos. #1#2, two different B2 ASOs. (**B**) RT-qPCR validation of L1 and MERVL knockdown in L2C embryos. We used the same ASO sequences of L1 and MERVL as the public papers (Percharde et al, 2018; Sakashita et al, 2023). (**C**) Representative images showing embryos treated with *Ythdf*1-3 siRNAs, *Ythdf*1-3 siRNAs + L1 ASO (0.2 µM), *Ythdf*1-3 siRNAs + MERVL ASO (0.2 µM), L1 ASO (0.2 µM), MERVL ASO (0.2 µM), or control siRNA at 4.5 dpc. Scale bars, 100 μm. (**D**) Percentages of embryonic stages observed at the indicated time points in Control (*n* = 47), siDF1-3 (*n* = 55), siDF1-3 + L1 ASO (*n* = 35), siDF1-3 + MERVL ASO (*n* = 50), L1 ASO (*n* = 30), MERL ASO (*n* = 40) groups. (**E**) RT-qPCR validation of 2 C genes in control, siDF1-3, siDF1-3 + L1 ASO, and siDF1-3 + MERVL ASO-treated embryos at 4.5 dpc. (**F**) Representative images showing embryos treated with DF1-3 siRNAs, 10×MERVL rescue (siDF1-3 + 2 µM of MERVL ASO),50×MERVL rescue (siDF1-3 + 10 µM of MERVL ASO), or control siRNA at 4.5 dpc. Scale bars, 100 μm. (**G**) Percentages of embryonic stages observed at the indicated time points in control (*n* = 30), siDF1-3 (*n* = 36), 10×MERVL rescue (*n* = 34), and 50×MERVL rescue (*n* = 31) groups. *P* values in (**D**, **G**) were determined by the Chi-square test. (**H**) RT-qPCR validation of 2 C genes in control, siDF1-3, and 10×MERVL rescue embryos at 4.5 dpc. Data in (**A**, **B**, **E**, **H**) are mean ± SD, *n* = 3 biological replicates. The *P* value was determined by a two-tailed unpaired *t* test. Source data are available online for this figure.

Then, we investigated whether reducing endogenous B2 RNAs could alleviate the developmental abnormalities observed in si*Ythdf1-3* knockdown embryos. To achieve this, we concurrently depleted B2 RNA and DF1-3 by microinjecting B2-specific antisense ASOs and *Ythdf1-3* siRNAs into the PN2 zygotes (Fig. EV5A). As expected, depletion of B2 RNA significantly promoted the development and blastocyst formation of *Ythdf1-3* knockdown embryos, accompanied by a marked reduction in the expression of 2C genes at the blastocyst stage (Fig. 4H–J), suggesting that reducing the abundance of B2 RNAs could effectively rescue the defects observed in si*Ythdf1-3* knockdown embryos. Markedly, depleting B2 RNAs alone in wild-type embryos also resulted in developmental delay and reduced blastocyst formation (Fig. 4H,I), hinting that the modest abundance of B2 RNA is crucial for embryonic development. In contrast, we observed that LINE1 ASO aggravated the defects of si*Ythdf1-3* knockdown embryos rather than rescuing the blastocyst rate, and MERVL ASO slightly improved the blastocyst rate of si*Ythdf1-3* knockdown embryos (Fig. EV5B–D). The expression levels of 2C genes in both LINE1 ASOs and MERVL ASOs rescuing embryos are higher or comparable to si*Ythdf1-3* knockdown embryos (Fig. EV5E), suggesting that knocking down either LINE1 or MERVL RNAs does not effectively rescue *Ythdf1-3* knockdown embryos. To further validate the rescue effect of MERVL, we increased the concentration of MERVL ASOs in si*Ythdf1-3* knockdown embryos. Surprisingly, instead of rescuing the defects, the embryos exhibited aggravated phenotypes, with 2C gene expression levels remaining comparable to those in *Ythdf1-3* knockdown embryos (Fig. EV5F–H). Thus, these results demonstrate that DF proteins promote retrotransposon RNA decay in early embryos, and the phenotype caused by *Ythdf1-3* knockdown is predominantly attributed to B2 RNA accumulation.

### B2 RNA regulates Pol II transcription in a *trans*-regulatory manner

**Figure 5:**
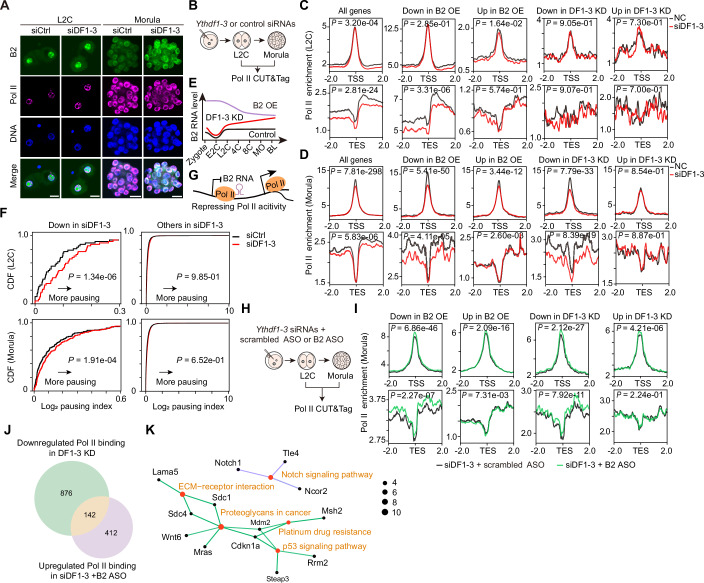
B2 regulates Pol II transcription in a *trans*-active manner upon *Ythdf1-3* knockdown. (**A**) Representative images of IF/RNA-FISH for B2 RNA with Pol II counterstain in control and si*Ythdf1-3* knockdown embryos at the L2C and morula stages. Scale bars, 20 µm. (**B**) Schematic of the experimental procedure for performing Pol II CUT&Tag in si*Ythdf1-3* knockdown and control embryos at the L2C and morula stages. (**C**, **D**) Profiles of Pol II levels on all gene loci (left), DEG loci of B2 overexpression (middle), and DEG loci of si*Ythdf1-3* knockdown (right) from 2.0 kb up to downstream of the transcription start site (TSS) or the transcription end site (TES) in control and si*Ythdf1-3* knockdown L2C (**C**) and morula (**D**) embryos. *P* values were determined with the Kolmogorov–Smirnov test. (**E**) Schematic depicting the dynamic expression levels of B2 RNAs in early embryos with si*Ythdf1-3* knockdown or B2 overexpression. (**F**) Cumulative distributions of the log_2_ pausing index in downregulated genes (left) and other genes (right) for Pol II binding between si*Ythdf1-3* knockdown and control embryos at the L2C (upper) and morula (bottom) stages. *P* values were determined with the Kolmogorov–Smirnov test. (**G**) Schematic of B2 RNA repressing Pol II transcription. (**H**) Schematic of the experimental procedure for performing Pol II CUT&Tag in si*Ythdf1-3* knockdown and B2 rescue embryos at the L2C and morula stages. (**I**) Profiles of Pol II levels on DEG loci of B2 overexpression (left) and *Ythdf1-3* knockdown (right) from 2.0 kb up- to downstream of the TSS (upper) or the TES (bottom) in si*Ythdf1-3* knockdown and B2 ASO rescue embryos at the morula stage. *P* values were determined with the Kolmogorov–Smirnov test. (**J**) Venn diagram showing the overlapping genes identified from downregulated Pol II binding signals in si*Ythdf1-3* knockdown embryos and upregulated Pol II binding signals in B2 ASO rescue embryos. (**K**) KEGG analysis of the overlapping genes identified from downregulated Pol II binding signals in si*Ythdf1-3* knockdown embryos and upregulated Pol II binding signals in B2 ASO rescue embryos. Source data are available online for this figure.

B2 RNAs are known to play roles in stress responses, such as during heat shock, by integrating into the pre-initiation complex of RNA Polymerase II (Pol II) at gene promoters and inhibiting transcriptional elongation, thereby functioning as a *trans*-regulator (Ponicsan et al, 2015; Zovoilis et al, 2016). We investigated whether the excessive accumulation of B2 RNAs blocks Pol II elongation in si*Ythdf1-3* knockdown embryos. Initially, we observed that B2 RNAs primarily localized in the nucleolus, separate from Pol II, in L2C embryos. However, at the morula stage, B2 RNAs colocalized with Pol II in the nucleus (Fig. 5A), suggesting a potential interaction between B2 RNAs and Pol II in morula embryos. To decipher the underlying mechanism, we performed CUT&Tag (Kaya-Okur et al, 2019) for Pol II in si*Ythdf1-3* knockdown and control embryos at the L2C and morula stages (Fig. 5B; Appendix Fig. S5A). At the L2C stage, Pol II binding at promoters was globally similar in both *Ythdf1-3* knockdown and control embryos (Fig. 5C), indicating that initial Pol II binding is not affected by si*Ythdf1-3* knockdown. However, transcriptional elongation of Pol II on the gene body was impaired in si*Ythdf1-3* knockdown embryos compared to control embryos. Conversely, in morula embryos, the Pol II binding features were reversed (Fig. 5D), with a reduction of Pol II binding signals at the promoter region but not at the transcription termination sites (TES) region in si*Ythdf1-3* knockdown embryos, suggesting that the transcriptional control of Pol II becomes more complex with prolonged si*Ythdf1-3* knockdown embryos. Intriguingly, the downregulated genes identified from si*Ythdf1-3* knockdown and B2 OE embryos were more likely to exhibit repressed transcriptional elongation (around the TES regions) of Pol II in si*Ythdf1-3* knockdown embryos compared to controls. In contrast, the upregulated genes did not exhibit enhanced Pol II transcriptional elongation. Note that the number of up-and downregulated genes (76 and 161, respectively) identified from si*Ythdf1-3* knockdown L2C embryos was so small that evaluating the difference in Pol II binding between si*Ythdf1-3* knockdown and control embryos was challenging. The repression of Pol II elongation for downregulated genes is enhanced by the accumulation of B2 RNA from L2C to morulae with si*Ythdf1-3* knockdown. Conversely, this repression is diminished by the decrease of B2 RNA from the L2C stage to morulae in B2 OE embryos (Fig. 5E). Furthermore, we evaluated the changes of the pausing index (Williams et al, 2015) (the ratio of Pol II signal density near a gene promoter to signal density within the gene body) for downregulated genes from si*Ythdf1-3* knockdown embryos in our Pol II CUT&Tag datasets. Downregulated genes showed a significantly increased pausing index in si*Ythdf1-3* knockdown embryos compared to other genes (unaffected and upregulated genes) (Fig. 5F), suggesting that B2 RNA can sequester Pol II at the gene promoter, thereby inhibiting Pol II elongation. Thus, these data suggest that the excessive accumulation of B2 RNA is more likely to repress Pol II elongation, contributing to gene downregulation at the transcriptional level in si*Ythdf1-3* knockdown embryos. However, the upregulated genes in si*Ythdf1-3* knockdown embryos result from the loss of RNA degradation rather than increased transcription, indicating that post-transcriptional regulation plays a more significant role for these genes. To confirm the role of B2 RNAs in Pol II transcription repression (Fig. 5G), we performed CUT&Tag for Pol II in si*Ythdf1-3* knockdown and B2 ASO rescue (siDF1-3 + B2 ASO) embryos at the L2C and morula stages (Fig. 5H; Appendix Fig. S5B). The downregulated genes from both si*Ythdf1-3* knockdown and B2 OE embryos showed enhanced Pol II transcriptional elongation in B2 ASO rescue embryos compared to si*Ythdf1-3* knockdown embryos, whereas the upregulated genes did not exhibit increased Pol II elongation (Fig. 5I), confirming that DF-mediated RNA decay plays a more significant role in the upregulation of these genes. Additionally, in L2C embryos, there were no apparent changes in Pol II elongation for both up-and-downregulated genes, aligning with the marginal colocalization of Pol II with B2 RNAs at the L2C stage (Appendix Fig. S5C). We next identified 142 genes whose expression was repressed by B2 RNA, based on changes in Pol II binding signals observed in *Ythdf1-3* knockdown and B2 ASO rescue embryos (Fig. 5J; Dataset EV7). Analysis of the 142 B2-repressed genes at the morula stage reveals their association with critical pathways involved in embryonic development, specifically the p53 signaling pathway and the Notch signaling pathway (Adiga et al, 2007; Huppert et al, 2000) (Fig. 5K). Additionally, beyond the repressive effect of B2 RNA on Pol II transcription, we found that m^6^A-marked genes also exhibited reduced Pol II transcription in *Ythdf1-3* knockdown embryos (Appendix Fig. S5D), concordance with the report that the transcription activation induced by m^6^A depletion in mESCs (Liu et al, 2020b). This result supports that the abundance of DF-targeted m^6^A-marked transcripts is susceptible to transcription regulation, despite their tendency to exhibit increased stability upon DF protein knockdown.

### B2 transcription modules RNA Pol II activity at genomic regions adjacent to B2 loci in early embryos

**Figure 6:**
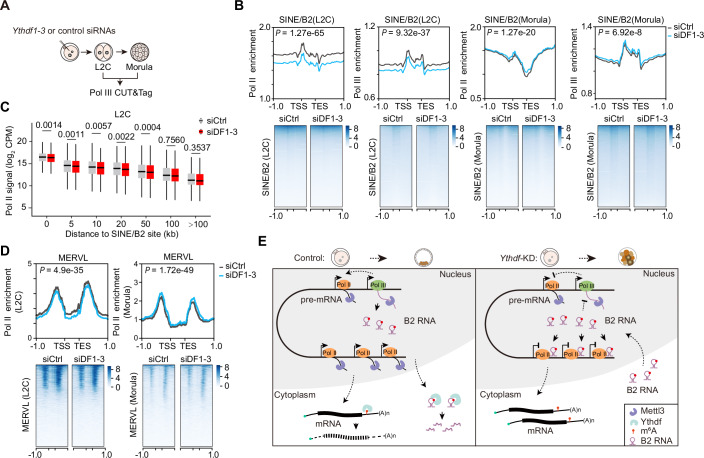
B2 transcription modulates Pol II activity adjacent to B2 loci. (**A**) Schematic of the experimental procedure for performing Pol Ⅲ CUT&Tag in si*Ythdf1-3* knockdown and control embryos at the L2C and morula stages. (**B**) Profiles and heatmaps of Pol II and Pol III binding on B2 RNA loci from 1.0 kb upstream of the TSS to 1.0 kb downstream of the TES in control and si*Ythdf1-3* knockdown embryos at the L2C and morula stages. *P* values were determined with the Kolmogorov–Smirnov test. (**C**) Box plots showing Pol II signal fold changes between si*Ythdf1-3* knockdown and control embryos at the L2C stage. The Pol II signals were categorized according to their genomic distance to the nearest B2 loci. B2 loci (0 kb), *n* = 7052; B2 loci (0-5 kb), *n* = 4503; B2 loci (5–10 kb), *n* = 3099; B2 loci (10–20 kb), *n* = 4064; B2 loci (20–50 kb), *n* = 6177; B2 loci (50–100 kb), *n* = 4468; B2 loci ( > 100 kb), *n* = 6961. Boxes represent the 25th-75th percentile (line at the median), with whiskers at 1.5× interquartile range (IQR). *P* values were determined with the Kolmogorov–Smirnov test. (**D**) Profiles and heatmaps of Pol II binding on MERVL loci from 1.0 kb upstream of the TSS to 1.0 kb downstream of the TES in control and si*Ythdf1-3* knockdown embryos at the L2C and morula stages. *P* values were determined by the Kolmogorov–Smirnov test. (**E**) A model illustrating that DF1/2/3 proteins mediate the interaction between B2 RNA and Pol II activity in early embryos. Each DF protein can promote the decay of mRNAs and B2 RNAs. Knockdown of *Ythdf1-3* leads to the retention of maternal RNAs, persistent expression of 2C genes, and accumulation of B2 RNAs, which in turn broadly repress RNA polymerase II transcription in a *trans*-acting manner.

In mammals, B2 RNAs are transcribed by Pol III, and it has been suggested that Pol III-occupied SINEs act as promoters or enhancers for nearby protein-coding genes (Ferrigno et al, 2001; Zhang et al, 2019). We sought to explore the potential *cis*-regulatory activity of SINE/B2 elements on Pol II transcription in *Ythdf1-3* knockdown embryos. To address this, we conducted CUT&Tag for Pol III in si*Ythdf1-3* knockdown and control embryos at the L2C and morula stages (Fig. 6A; Appendix Fig. S5E). Pol III binding signals were preferentially enriched in the tDNA (transfer RNA gene) locus compared to the genic regions, in contrast to Pol II binding signals, in line with the known roles of Pol III in transcribing transfer RNAs (tRNAs), and Pol II primarily transcribes mRNAs, despite the cross-regulatory effects observed between these RNA polymerases (Jiang et al, 2022) (Appendix Fig. S5F,G). At the L2C stage, *Ythdf1-3* knockdown embryos showed reduced transcription of Pol III-driven B2 RNAs, which corresponded with diminished Pol II transcription near SINE/B2 loci (Fig. 6B). In contrast, B2 RNA transcription was mildly elevated in si*Ythdf1-3* knockdown embryos, along with the Pol II transcription increase around SINE/B2 loci at the morula stage (Fig. 6b), implying that Pol II transcription around SINE/B2 loci is positively associated with B2 RNA transcription. Moreover, we observed a significant reduction in Pol II signal near SINE/B2 loci (≤50 kb), whereas the regions located farther away (>50 kb) remained unaffected at the L2C stage (Fig. 6C). Notably, in si*Ythdf1-3* knockdown morulae, Pol II signals appear largely unaffected by distance from SINE/B2 loci (Appendix Fig. S5H), consistent with a mild increase in Pol II transcription near these loci (Fig. 6B). These findings support a model in which B2 RNA transcription positively regulates adjacent Pol II activity in a *cis*-acting manner. In addition, Pol II-driven MERVL transcription activity is similar to Pol III-driven B2 RNA transcription in si*Ythdf1-3* knockdown embryos at the L2C and morula stages (Fig. 6D), suggesting that the abundance of B2 RNAs, along with MERVL, is regulated by both transcriptional and post-transcriptional levels in early embryos. In summary, B2 RNAs repress protein-coding gene transcription by *trans*-regulating Pol II elongation; meanwhile, they also repress Pol II transcription around the B2 locus through *cis*-regulation, contributing to gene downregulation in si*Ythdf1-3* knockdown embryos.

## Discussion

The DF1/2/3 proteins serve as primary cytosolic m^6^A-binding proteins, playing key roles in mediating the effects of m^6^A-marked transcripts in the cytosol (Shi et al, 2019). However, the exact mechanisms by which m^6^A and its binding proteins regulate gene expression and development are still largely unknown. In this study, we have systematically presented findings highlighting the critical role of m^6^A modification in RNA decay during mouse embryonic development, mediated by its reader proteins DF1/2/3 (Fig. 6E). Depletion of DF2 or DF3, but not DF1, compromised murine preimplantation development, with the most severe phenotype observed upon *Ythdf1-3* simultaneous knockdown, accompanied by abnormal gene expression and accumulation of retrotransposon RNAs. Notably, compensatory effects and redundant targets among DF proteins may underscore the dispensable role of DF1 in early embryos, despite its capacity, alongside DF2 and DF3, to promote RNA decay. Our results are consistent with a previous study showing that the knockdown of *Ythdf2* or *Ythdf3* delays somatic cell reprogramming, whereas *Ythdf1* knockdown does not affect this process (Liu et al, 2020a). For the DF paralogs, several post-translational modifications (PTMs), including phosphorylation, ubiquitination, SUMOylation, and glycosylation, have been reported to modulate their subcellular localization, ligand or partner interactions, and stress granule (SG) assembly (Chen et al, 2023; Sikorski et al, 2023). Recent studies further demonstrate that m⁶A-modified mRNAs can undergo liquid-liquid phase separation (LLPS) with DF proteins, resulting in the formation of endogenous LLPS compartments that facilitate compartment-specific mRNA regulation (Fu and Zhuang, 2020; Gao et al, 2019; Ries et al, 2019; Wang et al, 2019). Together, these intrinsic properties of DF proteins may ultimately influence their compensatory efficiency during early embryonic development. During early development, we observe a distinct expression pattern of DF proteins, with each protein contributing to the regulation of RNA decay and exhibiting similar m^6^A recognition capabilities. These findings support the unified model of YTHDF-mediated mRNA decay and reveal a critical YTHDF–SINE/B2–gene regulatory axis operating during early embryogenesis. The potential translation-promoting role of DF proteins in early embryos, however, warrants further investigation.

The enhanced stability of RNAs in si*Ythdf1-3* knockdown embryos correlated with the accumulation of transcripts from 2C genes, maternal RNAs, MGA transcripts, MERVL RNAs, and B2 RNAs. Furthermore, the developmental abnormalities induced by si*Ythdf1-3* knockdown were significantly alleviated by silencing B2 RNA, underscoring the critical role of B2 RNAs as targets of DF proteins. Although B2 RNAs are abundant in germ cells and early embryos, their functional roles are less reported. Previous studies have shown that B2 RNAs can be incorporated into the Pol II pre-initiation complex at gene promoters, thereby inhibiting transcriptional elongation both in vitro and in vivo, particularly under stress conditions (Ponicsan et al, 2015; Zovoilis et al, 2016). Given the accumulation of B2 RNAs and the widespread downregulation of genes observed in si*Ythdf1-3* knockdown embryos, we hypothesized that elevated B2 RNA levels could repress the transcription of a subset of genes, thereby contributing to their downregulation. Our analysis of Pol II and Pol III CUT&Tag datasets confirms that downregulated genes in *Ythdf1-3* knockdown embryos are more likely to exhibit suppressed Pol II transcription than upregulated genes. In addition to their *trans*-regulatory role, we observed that SINE/B2 elements repress Pol II transcription adjacent to SINE/B2 loci through *cis*-regulatory activity at the L2C stage, suggesting they have dual or multiple roles in RNA metabolism during early development.

In embryonic stem cells and early embryos, various classes of autonomous TEs are actively transcribed and marked with m^6^A, including IAP, ERVK, and LINE1. The m^6^A modification, along with its binding proteins, plays critical roles in regulating chromatin modifications, gene expression, and the repression of retrotransposons (Chelmicki et al, 2021; Liu et al, 2020b; Liu et al, 2021; Wei et al, 2022; Xu et al, 2021). These mechanisms are essential for maintaining cellular identity and supporting proper development. B2 RNA is highly expressed in germ cells and early embryos but is rarely explored. We demonstrated that DF1/2/3 proteins bind to L1, LTR, and SINE retrotransposon RNAs, which are recognized depending on m^6^A modification. However, only MERVL and B2 RNAs show significant accumulation in *Ythdf1-3* knockdown embryos, indicating that the turnover of retrotransposon RNAs is regulated at both the transcriptional and post-transcriptional levels. The excessive accumulation of MERVL and B2 RNAs may contribute to the phenotypic abnormalities observed in *Ythdf1-3* knockdown embryos. Our rescue assays demonstrated that only B2 RNAs, but not LINE1 or MERVL, significantly contribute to the defects observed in *Ythdf1-3* knockdown embryos. Previous studies have highlighted the essential *cis*-acting functions of MERVL RNA in preimplantation development (Sakashita et al, 2023), which are not dramatically affected in *Ythdf1-3* knockdown embryos. Therefore, B2 RNAs emerge as the primary candidate retrotransposon RNAs impacting developmental potency.

In summary, we propose an expanded model in which the coordinated regulatory axis of Ythdf-SINE/B2-gene expression plays a critical role in safeguarding the embryonic transcriptome from the detrimental effects of dysregulated retrotransposon RNAs, 2C transcripts, maternal RNAs, and development-associated genes during early development. This protective role promotes embryogenesis, expanding our understanding of the regulatory functions of m^6^A in development.

## Methods

**Table:** Reagents and tools table

Reagent/resource	Reference or source	Identifier or catalog number
**Experimental models**		
C57BL/6J (*M. musculus*)	Guangdong Zhiyuan Biomedical Technology	ZY001
DBA2 (*M. musculus*)	Beijing HFK Bioscience	2018041222
**Antibodies**		
Anti-β-Tubulin	Tianjin Sungene Biotech	KM9007
Anti-YTHDF1	Abcam	ab220162
Anti-YTHDF1	Proteintech	17479-1-AP
Anti-YTHDF2	Abcam	ab246514
Anti-YTHDF2	Cell Signaling Technology	80014S
Anti-YTHDF3	Abcam	ab220161
Anti-OCT4	Santa Cruz Biotechnology	sc-5279
Anti-NANOG	Cell Signaling Technology	8822
Anti-CDX2	BioGenex	MU392A-UC
Anti-flag	Abclonal	AE005
Anti-RNA pol II	Active motif	39497
Anti-POLR3A	Abcam	ab96328
Alexa Fluor 594-conjugated goat anti-rabbit secondary antibody	Thermo Scientific	A11012
Alexa Fluor 488-conjugated goat anti-mouse secondary antibody	Thermo Scientific	A11029
AF647-labeled Goat Anti-Mouse IgG (H + L)	Beyotime	A0473
Anti-m6A	Synaptic Systems	202003
**Oligonucleotides and other sequence-based reagents**		
siRNA sequences	This study	Table EV1
Cas9 guide RNA sequences	This study	Table EV1
CasRx guide RNA sequences	This study	Table EV1
PCR primers	This study	Table EV1
B2 RNA-FISH probe sequences	This study	Table EV1
B2 ASO sequence	This study	Table EV1
LINE1 ASO sequence	Percharde et al, 2018	Table EV1
MERVL ASO sequence	Sakashita et al, 2023	Table EV1
**Chemicals, enzymes and other reagents**		
KSOM medium	Millipore	MR-107-D
T7 High Yield RNA Transcription Kit	Ambion	AM1344
Poly(A) polymerase tailing kit	Epicentre	PAP5104H
DRB	Sigma	D1916
STM2457	Selleck	S9870
PVDF membrane	Millipore	1620177
Wes Separation Module microplate	ProteinSimple	SM-W002
PicoPure RNA Isolation Kit	QIAGEN	74034
Maxima H Minus First Strand cDNA Synthesis Kit	Thermo Scientific	K1682
Power SYBR Green Master Mix	Vazyme	Q111
KAPA HiFi HotStart Ready Mix	KAPA Biosystems	KK2601
GenElute™ Single Cell RNA Purification Kit	Sigma	RNB300
Hybond-N+ membrane	GE Healthcare	RPN203B
20× SSC	Thermo Scientific	AM9770
Dextran Sulfate 50% Solution	Sigma	S4030
Formamide	Sigma	47671-250ML-F
Ribonucleoside vanadyl complexes (RVC)	Sigma	R3380-5ML
Tyrode’s solution	Sigma	T1788
Pierce chip-grade protein A/G magnetic beads	Thermo Scientific	26162
RNase inhibitor	Thermo Scientific	EO0381
RQ1 DNase	Promega	M6101
Micrococcal nuclease	New England BioLabs	M0247S
FastAP alkaline phosphatase	Thermo Scientific	EF0651
T4 RNA ligase 2	New England Biolabs	M0242
RNase H	Thermo Scientific	EN0202
Dynabeads™ MyOne™ Streptavidin C1 beads	Thermo Scientific	65002
Ampure XP beads	Beckman Coulter	A63881
T7 RNA Polymerase	New England BioLabs	M0251
TURBO DNase	Thermo Scientific	AM2238
Agencourt RNA Clean beads	Beckman Coulter	A63987
Gel Extraction Kit	Qiagen	28604
Hyperactive Universal CUT&Tag Assay Kit	Vazyme Biotech	TD903
Superscript II Reverse Transcriptase	Thermo Scientific	18064071
RiboLock RNase inhibitor	Thermo Scientific	EO0382
10 × PBS buffer	Thermo Scientific	AM9624
dNTP Solution Mix	New England BioLabs	N0447
Ribonucleotide Solution Mix	New England BioLabs	N0466
Qubit RNA HS Assay Kit	Thermo Scientific	Q32852
Qubit dsDNA HS Assay Kit	Thermo Scientific	Q32854
Glycogen	Thermo Scientific	AM9510
IGEPEL (NP-40)	Sigma	18896
Triton X-100	Sigma	T8787
T4 RNA Ligase 1	New England BioLabs	M0437M
Adenosine 5’-Triphosphate (ATP)	New England BioLabs	P0756S
Tween-20	Sigma	P9416
Roche Complete Protease Inhibitor EDTA-Free tablets	Roche	4693132001
**Software**		
GraphPad Prism v9.5.0	GraphPad Software	
Fiji: ImageJ	https://imagej.net/software/fiji/	
HISAT2 v2.2.0,	Kim et al, 2019	
featureCount v2.0.1	Liao et al, 2014	
DESeq2	Love et al, 2014	
TEtranscripts	Jin et al, 2015	
Cutadapt v1.8.1	Martin, 2011	
fastp v0.21.0	Chen et al, 2018	
Bowtie2 v2.5.1	Langmead and Salzberg, 2012	
STAR58 v2.5.2b	Dobin et al, 2013	
PureCLIP v 1.3.1	Krakau et al, 2017	
MACS3 v3.0.0b3	Zhang et al, 2008	
deepTools v3.5.4.	Ramírez et al, 2016	
Homer	http://homer.ucsd.edu/homer/	
WGCNA	Langfelder and Horvath, 2008	
ChIPSeeker	Yu et al, 2015	
**Other**		
Roche LightCycler 480 instrument	Roche	
Nikon TI2-U microscope	Nikon	
Illumina NovaS4 sequencer	Illumina	
Abby™ Simple Western™ system	ProteinSimple	

### Animals

Eight-week-old female C57BL/6 and male DBA/2 mice were maintained under SPF conditions at the Animal Center of Guangdong Second Provincial General Hospital. All animal maintenance and experimental procedures were conducted following the guidelines of the Institutional Animal Care and Use Committee of Guangdong Second Provincial General Hospital, Guangzhou, China.

### Embryo collection and in vitro culture

For embryo collection, C57BL/6 female mice were injected with 7.5 IU of pregnant mare serum gonadotropin (PMSG), followed by an injection of human chorionic gonadotropin (hCG) 48 h later, with a dosage of 5 IU. Then, female mice were mated with DBA/2 males after hCG administration. Zygotes were collected from the swollen upper part of the oviduct 18 h after hCG administration. Cumulus cells were removed from zygotes by briefly incubating them in the M2 medium with hyaluronidase. The zygotes were cultured in KSOM (Millipore, MR-107-D) droplets at 37 °C under 5% CO_2_.

### In vitro transcription (IVT)

The Cas9 sgRNAs were designed using the online gene editing tool (https://design.synthego.com/), and CasRx sgRNAs were designed using the online tool (https://cas13design.nygenome.org/). All sgRNA sequences were listed in Table EV1. A T7 promoter was added to the sgDNA, B2_Mm1a, Cas9, and CasRx DNA templates by PCR amplification. The T7-DNA PCR products were purified and used as the DNA templates for IVT at 37 °C for 4 h using the T7 High Yield RNA Transcription Kit (Ambion, AM1344). The Cas9 and CasRx mRNAs were then polyadenylated using a Poly(A) polymerase tailing kit (Epicentre, PAP5104H). The RNAs were purified using RNA Clean Beads according to the manufacturer’s protocols.

### Embryo microinjection

Zygote microinjection was performed on the heating stage of a phase-contrast inverted microscope (Nikon). Approximately 10 pl of siRNA solution (30 μM), B2 RNA (170-1.7 ng/μl), L1 ASO (0.2–20 μM), MERVL ASO (0.2–20 μM), and B2 ASO (0.2–20 μM), were microinjected into the cytoplasm of zygotes. For Cas9-mediated KO, Cas9 mRNA (150 ng/μl), mixed with different sgRNAs (300 ng/μl), was microinjected into the cytoplasm of zygotes. For CasRx-mediated KD, CasRx mRNA (150 ng/μl), mixed with different sgRNAs (300 ng/μl), was microinjected into the cytoplasm of zygotes. The zygotes were incubated in KSOM at 37 °C with 5% CO_2_.

### DRB and STM2457 treatment of mouse embryos

One-cell embryos were obtained at 18 h post-hCG and cultured in a KSOM medium containing 60 μM DRB (Sigma, D1916). These embryos were collected after 25 h (L2C).

For STM2457 treatment, we used 10 μM STM2457 (Selleck, S9870) in the KSOM medium and 0.1% DMSO in the KSOM medium as a control.

### Western blotting

Embryos were collected in 10 μl lysis buffer and stored at −80 °C until use. Samples were then mixed with protein sample buffer (Beyotime, P0012B) and heated at 95 °C for 10 min. Protein samples were separated by SDS-PAGE using electrophoresis systems and transferred to a polyvinylidene fluoride (PVDF) membrane (Millipore, 1620177). Then, the membrane was blocked with a blocking buffer (Beyotime, P0252) for 15 min and incubated with primary antibodies overnight at 4 °C. After rinsing three times in PBST, the membranes were incubated for 1 h with secondary antibodies. After rinsing three times in PBST, the membrane was imaged with Tanon ECL Ultra (Tanon), and the images were scanned using the VersaDoc Imaging System (Bio-Rad).

### Simple western analysis

Embryos were collected in a 4.5 µl loading buffer and denatured at 95 °C for 5 min. After cooling on ice for 5 min, 3 µl lysate was loaded to the Wes Separation Module microplate (SM-W002) for analysis according to the manufacturer’s instructions (ProteinSimple).

### Quantitative RT-PCR

Total RNA was extracted from embryos using the PicoPure RNA Isolation Kit with on-column genomic DNA digestion (QIAGEN, 74034) and reverse transcribed using the RT Reagent Kit (Thermo Scientific, K1682) with oligo dT and random hexamer primers. For Quantitative RT-PCR, the resulting cDNA was quantified by real-time PCR on a LightCycler 480 instrument (Roche) using Power SYBR Green Master Mix (Vazyme, Q111). Relative gene expression was analyzed based on the 2^−ΔΔCt^ method, normalized with GAPDH. For RT-PCR, the resulting cDNA was amplified by KAPA HiFi HotStart Ready Mix (KAPA Biosystems, KK2601). The PCR products were separated and imaged on a 2.5% agarose gel. All RT-qPCR primers used in this study are listed in Table EV1.

### Dot blot

m^6^A Dot blot assay was performed following the previous protocol (Shen et al, 2017). Total RNA was extracted from embryos using the GenElute™ Single Cell RNA Purification Kit (Sigma, RNB300). RNA was denatured at 95 °C for 3 min and transferred to ice immediately. After dropping 3 μl of RNA directly onto the Hybond-N+ membrane (GE Healthcare, RPN203B), the spotted RNA was cross-linked to the membrane in a Crosslinker twice with UV light: 200 mJoule/cm^2^ at 254 nM. The membrane was washed in Wash buffer (1x PBS, 0.02% Tween-20) for 5 min at room temperature (RT) and incubated in Blocking buffer (1x PBS, 0.02% Tween-20, 5% non-fat milk) for 1 h at RT. The membrane was then incubated with anti-m^6^A antibody (1:250 dilution) in antibody dilution buffer (1× PBS 0.02% Tween-20 5% non-fat milk) overnight at 4 °C. After washing the membrane three times for 5 min each in Wash buffer, the membrane was incubated with goat anti-rabbit IgG-HRP (1:10,000 dilution; 20 ng/ml) in Antibody dilution buffer for 1 h at RT. After washing the membrane four times for 10 min each in Wash buffer, the membrane was incubated with ECL Western Blotting Substrate for 5 min in the dark at RT. Then, the membrane was imaged with Tanon ECL Ultra (Tanon), and the images were scanned using the VersaDoc Imaging System (Bio-Rad).

### Immunostaining

Embryos were fixed in 4% paraformaldehyde at room temperature for 30 min. Then, the embryos were permeabilized with 0.5% Triton X-100 at room temperature (RT) for 30 min. The samples were then blocked with 10% FBS at room temperature for 1 h. After blocking, the embryos were incubated overnight at 4 °C with the primary antibody. The following day, the embryos were washed three times with 0.1% PVP in PBS for 5 min each and then incubated with Alexa Fluor 488-conjugated anti-mouse and/or 594-conjugated anti-rabbit immunoglobulin G secondary antibodies (Thermo Scientific, A11029, A11012; 1:500) for 1 h at RT. After washing the embryos three times with 0.1% PVP in PBS for 10 min each, they were counterstained with DAPI for 15 min at RT and then loaded onto glass slides. Images were obtained with a confocal laser scanning microscope (Leica DMi8) and processed using ImageJ (NIH).

### RNA-FISH and immunofluorescence co-staining

Embryos were fixed in 4% paraformaldehyde for 10 min at RT, infiltrated in PBST/RVC buffer (1× PBS, 2 mM ribonucleoside vanadyl complexes (RVC), 0.5% Triton X-100) for 10 min at 4 °C. After that, the samples were permeabilized twice with 70% ethanol, 90% ethanol, and 100% ethanol for 5 min each at 4 °C. Then, they were hybridized overnight at 37 °C in a hybridization buffer (55% formamide, 11% dextran, 11% 20× SSC, 0.11% BSA, and 22 mM RVC) using probes specifically designed to target the entire length of B2_mm1a RNA (Table EV1). After washing twice with FA/2×SSC buffer (50% Formamide, 2×SSC, DEPC-treated water) and 2×SSC for 10 min each, the nuclei of the embryos were counterstained with DAPI for 10 min at RT. All images were collected using a confocal laser scanning microscope (Leica DMi8) and processed with ImageJ (NIH).

B2 FISH/immunofluorescence co-staining was performed following the previous protocol (Namekawa and Lee, 2011). Embryos were fixed in 1% paraformaldehyde on ice for 10 min. After that, the samples were permeabilized in 70% (vol/vol) ethanol at 4 °C until use. After incubating with a blocking buffer (1% BSA, 0.1% TWEEN 20, 0.4 U/μl SUPERase•In™ RNase Inhibitor in PBS), the primary antibody at a 1:100 dilution in blocking buffer was incubated for 1 h at room temperature. Samples were washed with blocking buffer and stained with Alexa 594/647-conjugated secondary antibody (Thermo Scientific, A11012, 1:500; Beyotime, A0473, 1:500) for 1 h. Then samples were fixed again with 4% PFA for 10 min. After dehydrating slides by serial treatment with 70%, 80%, and 100% (vol/vol) ethanol, samples were hybridized with FISH probes at 42 °C overnight. After washing twice with FA/2×SSC buffer (50% Formamide, 2×SSC, DEPC-treated water) and 2×SSC, the nuclei of the embryos were counterstained with DAPI for 10 min at RT. For all the experiments mentioned in this section, the images were collected using a confocal laser scanning microscope (Leica DMi8). The intensity of FISH signals was quantified using Fiji (ImageJ).

### RNA-seq

RNA-seq libraries were generated following the Smart-seq2 protocol (Picelli et al, 2014), as described previously. The zona pellucida was gently removed with Tyrode’s solution (Sigma, T1788). Oocytes and embryos were washed three times with 0.1% BSA in PBS and then lysed in 4 μl lysis buffer containing RNase inhibitor.

### LACE-seq

LACE-seq was performed based on our previously described protocol (Su et al, 2021) with minor modifications. Briefly, 250–300 ZP-free two-cell or morula embryos were cross-linked with 0.1% formaldehyde at RT for 10 min and then quenched with 150 mM glycine at RT for 10 min. The cross-linked samples were collected in a 1.5 ml low-binding tube.

For each sample, 10 μl protein A/G magnetic beads (Thermo Scientific, 26162) were blocked with block buffer (1× PBS, 0.2 mg/ml glycogen, 0.2 mg/ml BSA) at RT for 1 h, then washed once with 0.1 M Na-phosphate buffer (93.2 mM Na_2_HPO_4_, 6.8 mM NaH_2_PO_4_, 0.05% Tween 20, pH 8.0). The blocked beads were resuspended in 50 µl of 0.1 M Na-phosphate buffer containing 2 µg YTHDF antibody at RT for 1 h. The antibody-coupled beads were washed twice with wash buffer (1× PBS, 0.1% SDS, 0.5% NP-40, 0.5% sodium deoxycholate) and resuspended in 10 µl of wash buffer per sample.

The cross-linked samples were lysed on ice with 50 µl of wash buffer for 10 min. Then, 1 µl of RNase inhibitor (Thermo Scientific, EO0381) and 4 µl of RQ1 DNase (Promega, M6101) were added, and the mixture was then incubated at 37 °C for 3 min. After snap-chilling the tube on ice for 3 min, 10 µl of beads coupled with antibodies were added to the lysate and rotated for 1 h at 4 °C. The immunoprecipitated RNAs were fragmented with 1 × 10^−8^ U micrococcal nuclease (MNase, New England BioLabs, M0247S) for 3 min at 37 °C. The fragmented RNA 3’ ends were dephosphorylated on beads using FastAP alkaline phosphatase (Thermo Scientific, EF0651) at 37 °C for 10 min. The fragmented RNA 3′ linker was ligated with T4 RNA ligase 2 (New England Biolabs, M0242) at RT for 2.5 h.

After converting the RNA fragment to cDNA using a biotin-modified primer, the cDNA was released from Protein A/G beads by treating it with RNase H (Thermo Scientific, EN0202) and then captured using streptavidin C1 beads (Thermo Scientific, 65002). After the cDNA 3’ linker was ligated with T4 RNA ligase 1 (New England BioLabs, M0437) overnight at RT, the cDNA products were subjected to pre-PCR using KAPA HiFi HotStart Ready Mix (KAPA Biosystems, KK2601). The PCR products were purified using Ampure XP beads (Beckman Coulter, A63881) and subjected to in vitro transcription with T7 RNA Polymerase (New England BioLabs, M0251) at 37 °C for 24 h. DNA template was removed by treating it with TURBO DNase (Thermo Scientific, AM2238) at 37 °C for 30 min and the RNA was purified using Agencourt RNA Clean beads (Beckman Coulter, A63987). After performing reverse transcription and indexed PCR, the PCR products were subjected to size selection using a 2% agarose gel. Regions ranging from 250 to 500 bp were then purified using a Gel Extraction Kit (Qiagen, 28604).

### CUT&Tag

CUT&Tag was performed as previously described (Kaya-Okur et al, 2019) using the Hyperactive Universal CUT&Tag Assay Kit for Illumina (Vazyme Biotech, TD903). In brief, 50 embryos were incubated with 10 μl pre-washed ConA beads in a 1.5-ml low-binding tube. 50 μl of pre-cooled antibody buffer containing 0.8 μg primary antibody was added to the tube and incubated overnight at 4 °C. After removing the supernatant, 50 μl dig-wash buffer with 0.5 μg secondary antibody was added to resuspend the ConA beads and incubated at RT for 1 h. After washing three times with 200 μl dig-wash buffer, 1 μl pA/G-Tnp was added with 100 μl dig-300 buffer. Samples were incubated at room temperature for 1 h and then washed thrice with 200 μl dig-300 buffer. In total, 40 μl dig-300 buffer was added with 10 μl 5× TTBL, and the samples were incubated at 37 °C for 1 h. The reaction was stopped by adding 5 μl Proteinase K, 100 μl Buffer L/B, and 20 μl DNA Extract Beads. PCR was performed to amplify the libraries after extracting the DNA with DNA extraction beads. The libraries were extracted using VAHTS DNA Clean Beads and sequenced on the Illumina NovaSeq platform, following the manufacturer’s instructions.

### Data analyses

#### RNA-seq data analysis

All RNA-seq data were aligned to the mm9 genome by HISAT2 (Kim et al, 2019) (version 2.1.0). The read count was calculated by featureCount (Liao et al, 2014) (version 2.0.1) with the Gencode mm9 annotation. The count data was normalized using the estimateSizeFactors function and then subjected to the DESeq2(Love et al, 2014) package for differential expression analysis. For TE analysis, multi-aligned reads were retained and subjected to TEtranscripts (Jin et al, 2015) for quantifying TE families and conducting differential expression analysis. For published RNA-seq data, the expression values were calculated using a similar method.

#### LACE-seq data analysis

The adapter sequences at both ends of the raw reads were removed using the Cutadapt (Martin, 2011) program (v1.15) with the following parameters: -m 18 -j 8 --max-n 4 --trim-n --times 2 -e 0.1 -O 3. The sequences for parameters -a, -A, and -G are listed in Table EV3. Paired-end reads were merged into single reads using fastp (Chen et al, 2018) (version 0.21.0) if there was an overlap of more than 30 nucleotides. After extracting the UMI sequence, the clean reads were initially aligned to the mouse pre-rRNA using Bowtie2 (Langmead and Salzberg, 2012) software (version 2.5.1), and the remaining unmapped reads were subsequently aligned to the mouse (mm9) reference genome using STAR (Dobin et al, 2013) (version 2.5.2b) with the following parameters: --outSJfilterReads Unique --alignEndsType Extend5pOfRead1 --outFilterMismatchNoverLmax 0.04 --outFilterMismatchNmax 999 --outFilterMultimapNmax 1. UMI sequences were used to remove PCR duplicates, and the retained reads were utilized for peak identification using PureCLIP (Krakau et al, 2017) (version 1.3.1). The 5’ ends of LACE-seq reads within a genomic distance shorter than 100 bp were merged for motif analysis. Merged ends with more than 10 unique reads were further selected as clusters, and the summit was defined as the position with the highest read end coverage. Genomic regions 500 bp around the summit were selected as the background region. Base enrichment around the summit was defined as the ratio over the corresponding background region. The well-known m^6^A motif, DRACH, was used to filter potential false-positive binding events for DF proteins. For motif analysis, LACE-seq peaks were first extended by 30 nt upstream, and overrepresented motifs in the extended sequences were identified using the findMotifsGenome.pl function in Homer (http://homer.ucsd.edu/homer/). The Pearson correlation coefficient between LACE-seq replicates was calculated as described previously.

#### picoMeRIP data analysis

We reanalyzed the picoMeRIP data from mouse 2C and 8C embryos (Wang et al, 2023), following the instructions of the original paper (https://github.com/Augroup/ MeRipBox). Briefly, sequencing adapters and low-quality bases were trimmed using Cutadapt (v1.8.1), with the parameters: -q 20,20 -m 20 -max-n 0.01 -trim-n. Trimmed reads were aligned to the mouse reference genome (mm9) using HISAT2 (v2.1.0) with the following parameters: -5 8 -no-mixed -no-discordant. Only the uniquely aligned reads were retained. PCR duplicates were removed using SAMtools fixmate & markdup, and the reads mapped to rRNAs were also removed. m^6^A peaks were called using MACS3 (Zhang et al, 2008) with the following parameters: -keep-dup all -B -nomodel -call-summits. Only the peaks with a *q* value < 0.05 were used for the subsequent analyses.

#### CUT&Tag data analysis

CUT&Tag raw reads were filtered using fastp (version 0.21.0) with the following options: -g -x -q 20 -l 36 -y -c -u 40 -n 4 --detect_adapter_for_pe -w 8. The filtered reads were then aligned to the indexed mouse genome (mm9) using Bowtie2 (version 2.5.1) with the following options: --local --very-sensitive-local --no-mixed --no-discordant --phred33 -I 10 -X 700 -p 10. Uniquely mapped reads were used for peak calling using MACS3 (version 3.0.0b3) with the following parameters: -nolambda -nomodel. A cut-off of q value ≤ 0.05 was applied. The average tag density plots were generated using the plotHeatmap program implemented in deepTools (Ramírez et al, 2016) (version 3.5.4). To visualize read enrichment over representative genomic loci, BigWig files were created from sorted BAM files using bamCoverage from deepTools. Genomic distributions were displayed using ChIPSeeker (Yu et al, 2015). Pausing index analysis was conducted following the instructions provided at https://github.com/MiMiroot/PIC. For the analysis of differential binding, we first generated a set of regions called peaks using MACS3. These peaks were identified in at least one of the samples. Then, we utilized the mergeBed function from BEDTools to combine the peak regions from all biological replicates. Using the merged peak file and CUT&Tag data, read counts for each region were quantified using featureCounts. Subsequently, the data were subjected to DESeq2 for differential analysis.

#### Gene expression pattern analysis

We reanalyzed the RNA-seq data (Deng et al, 2014) from MII oocytes and early embryos, following the instructions of the previous report (Park et al, 2013). We defined 13 types of stage-related gene sets. Specifically, gene expression was first averaged across developmental stages, and then hierarchical clustering was performed based on the Pearson correlation distance. The cutreeDynamic and mergeCloseModules functions from WGCNA (Langfelder and Horvath, 2008) were used to generate gene modules. Finally, the average expression pattern across the developmental stages for each gene module was used as a feature to classify them into the 13 types of stage-related gene sets.

### Statistics and reproducibility

All experiments were independently repeated at least twice, and no inconsistent results were observed. Statistical analyses were carried out using the GraphPad software or R Studio. The box borders in the boxplots represent upper and lower quartiles (25th and 75th percentiles), and the center line represents the median. The violin plots depict the distribution of the data, with the center line representing the median and the upper and lower quartiles corresponding to the 75th and 25th percentiles, respectively. The statistical tests and *P* values are indicated in the figure legends. *P* values < 0.05 were considered significant. All data are reproducible, and the details of replicates are stated in the figure legends.

## Supplementary information

Table EV1

Table EV2

Table EV3

Appendix

Peer Review File

Dataset EV1

Dataset EV2

Dataset EV3

Dataset EV4

Dataset EV5

Dataset EV6

Dataset EV7

Source data Fig. 1

Source data Fig. 2

Source data Fig. 4

Source data Fig. 5

Source data for Expanded View and Appendix figures

Expanded View Figures

## Data Availability

All the sequencing data generated in this paper have been deposited in the Genome Sequence Archive under project PRJCA021648, with accession CRA013737 (https://ngdc.cncb.ac.cn/gsub/submit/gsa/subCRA021795, and the referee access codes: https://ngdc.cncb.ac.cn/gsa/s/dXRgF890). Publicly available datasets downloaded and used in this work were from NCBI GEO accession number GSE192440. The source data of this paper are collected in the following database record: biostudies:S-SCDT-10_1038-S44318-026-00728-w.

## References

[CR1] Abe K-I, Funaya S, Tsukioka D, Kawamura M, Suzuki Y, Suzuki MG, Schultz RM, Aoki F (2018) Minor zygotic gene activation is essential for mouse preimplantation development. Proc Natl Acad Sci USA 115:E6780–E6788 29967139 10.1073/pnas.1804309115 PMC6055165

[CR2] Adiga SK, Toyoshima M, Shiraishi K, Shimura T, Takeda J, Taga M, Nagai H, Kumar P, Niwa OJO (2007) p21 provides stage specific DNA damage control to preimplantation embryos. Oncogene 26:6141–6149 10.1038/sj.onc.1210444 17420724

[CR3] Allen TA, Von Kaenel S, Goodrich JA, Kugel JF (2004) The SINE-encoded mouse B2 RNA represses mRNA transcription in response to heat shock. Nat Struct Mol Biol 11:816–821 15300240 10.1038/nsmb813

[CR4] Bachvarova R (1988) Small B2 RNAs in mouse oocytes, embryos, and somatic tissues. Dev Biol 130:513–523 2461884 10.1016/0012-1606(88)90346-6

[CR5] Boccaletto P, Machnicka MA, Purta E, Piątkowski P, Bagiński B, Wirecki TK, de Crécy-Lagard V, Ross R, Limbach PA, Kotter A (2018) MODOMICS: a database of RNA modification pathways. 2017 update. Nucleic Acids Res 46:D303–D307 29106616 10.1093/nar/gkx1030 PMC5753262

[CR6] Chelmicki T, Roger E, Teissandier A, Dura M, Bonneville L, Rucli S, Dossin F, Fouassier C, Lameiras S, Bourc’his D (2021) m6A RNA methylation regulates the fate of endogenous retroviruses. Nature 591:312–316 33442060 10.1038/s41586-020-03135-1

[CR7] Chen S, Zhou Y, Chen Y, Gu J (2018) fastp: an ultra-fast all-in-one FASTQ preprocessor. Bioinformatics 34:i884–i890 30423086 10.1093/bioinformatics/bty560 PMC6129281

[CR8] Chen Y, Wan R, Zou Z, Lao L, Shao G, Zheng Y, Tang L, Yuan Y, Ge Y, He C, Lin S (2023) O-GlcNAcylation determines the translational regulation and phase separation of YTHDF proteins. Nat Cell Biol 25:1676–1690 37945829 10.1038/s41556-023-01258-x PMC12060179

[CR9] Deng Q, Ramsköld D, Reinius B, Sandberg R (2014) Single-cell RNA-seq reveals dynamic, random monoallelic gene expression in mammalian cells. Science 343:193–196 24408435 10.1126/science.1245316

[CR10] Dobin A, Davis CA, Schlesinger F, Drenkow J, Zaleski C, Jha S, Batut P, Chaisson M, Gingeras TR (2013) STAR: ultrafast universal RNA-seq aligner. Bioinformatics 29:15–21 23104886 10.1093/bioinformatics/bts635 PMC3530905

[CR11] Elbarbary RA, Lucas BA, Maquat LE (2016) Retrotransposons as regulators of gene expression. Science 351: aac7247 26912865 10.1126/science.aac7247 PMC4788378

[CR12] Ferrigno O, Virolle T, Djabari Z, Ortonne J-P, White RJ, Aberdam D (2001) Transposable B2 SINE elements can provide mobile RNA polymerase II promoters. Nat Genet 28:77–81 11326281 10.1038/ng0501-77

[CR13] Fu Y, Zhuang X (2020) m6A-binding YTHDF proteins promote stress granule formation. Nat Chem Biol 16:955–963 32451507 10.1038/s41589-020-0524-y PMC7442727

[CR14] Gao Y, Pei G, Li D, Li R, Shao Y, Zhang QC, Li P (2019) Multivalent m6A motifs promote phase separation of YTHDF proteins. Cell Res 29:767–769 31388144 10.1038/s41422-019-0210-3 PMC6796879

[CR15] Geula S, Moshitch-Moshkovitz S, Dominissini D, AlFatah Mansour A, Kol N, Salmon-Divon M, Hershkovitz V, Peer E, Mor N, S Manor Y et al (2015) m6A mRNA methylation facilitates resolution of naïve pluripotency toward differentiation. Science 347:1002–1006 25569111 10.1126/science.1261417

[CR16] Guo X, Rahman JA, Wessels H-H, Méndez-Mancilla A, Haro D, Chen X, Sanjana NE (2021) Transcriptome-wide Cas13 guide RNA design for model organisms and viral RNA pathogens. Cell Genomics 1:100001 35664829 10.1016/j.xgen.2021.100001 PMC9164475

[CR17] Guo Y, Li TD, Modzelewski AJ, Siomi H (2023) Retrotransposon renaissance in early embryos. Trends Genet 40(1):39–51 37949723 10.1016/j.tig.2023.10.010 PMC12123636

[CR18] Huppert SS, Le A, Schroeter EH, Mumm JS, Saxena MT, Milner LA, Kopan RJN (2000) Embryonic lethality in mice homozygous for a processing-deficient allele of Notch1. Nature 405:966–970 10.1038/35016111 10879540

[CR19] Ivanova I, Much C, Di Giacomo M, Azzi C, Morgan M, Moreira PN, Monahan J, Carrieri C, Enright AJ, O’Carroll D (2017) The RNA m6A reader YTHDF2 is essential for the post-transcriptional regulation of the maternal transcriptome and oocyte competence. Mol Cell 67:1059–1067 28867294 10.1016/j.molcel.2017.08.003 PMC5613143

[CR20] Jiang Y, Huang J, Tian K, Yi X, Zheng H, Zhu Y, Guo T, Ji X (2022) Cross-regulome profiling of RNA polymerases highlights the regulatory role of polymerase III on mRNA transcription by maintaining local chromatin architecture. Genome Biol 23:1–37 36443871 10.1186/s13059-022-02812-w PMC9703767

[CR21] Jin Y, Tam OH, Paniagua E, Hammell M (2015) TEtranscripts: a package for including transposable elements in differential expression analysis of RNA-seq datasets. Bioinformatics 31:3593–3599 26206304 10.1093/bioinformatics/btv422 PMC4757950

[CR22] Kaya-Okur HS, Wu SJ, Codomo CA, Pledger ES, Bryson TD, Henikoff JG, Ahmad K, Henikoff S (2019) CUT&Tag for efficient epigenomic profiling of small samples and single cells. Nat Commun 10:1930 31036827 10.1038/s41467-019-09982-5 PMC6488672

[CR23] Kim D, Paggi JM, Park C, Bennett C, Salzberg SL (2019) Graph-based genome alignment and genotyping with HISAT2 and HISAT-genotype. Nat Biotechnol 37:907–915 31375807 10.1038/s41587-019-0201-4 PMC7605509

[CR24] Kontur C, Jeong M, Cifuentes D, Giraldez AJ (2020) Ythdf m6A readers function redundantly during zebrafish development. Cell Rep 33:108598 10.1016/j.celrep.2020.108598 PMC11407899 33378672

[CR25] Krakau S, Richard H, Marsico A (2017) PureCLIP: capturing target-specific protein–RNA interaction footprints from single-nucleotide CLIP-seq data. Genome Biol 18:1–17 29284540 10.1186/s13059-017-1364-2 PMC5746957

[CR26] Langfelder P, Horvath S (2008) WGCNA: an R package for weighted correlation network analysis. BMC Bioinforma 9:1–13 10.1186/1471-2105-9-559 PMC2631488 19114008

[CR27] Langmead B, Salzberg SL (2012) Fast gapped-read alignment with Bowtie2. Nat Methods 9:357–359 22388286 10.1038/nmeth.1923 PMC3322381

[CR28] Lasman L, Krupalnik V, Viukov S, Mor N, Aguilera-Castrejon A, Schneir D, Bayerl J, Mizrahi O, Peles S, Tawil S et al (2020) Context-dependent functional compensation between Ythdf m6A reader proteins. Genes Dev 34:1373–1391 32943573 10.1101/gad.340695.120 PMC7528697

[CR29] Lee MT, Bonneau AR, Giraldez AJ (2014) Zygotic genome activation during the maternal-to-zygotic transition. Annu Rev Cell Dev Biol 30:581–613 25150012 10.1146/annurev-cellbio-100913-013027 PMC4303375

[CR30] Li A, Chen Y-S, Ping X-L, Yang X, Xiao W, Yang Y, Sun H-Y, Zhu Q, Baidya P, Wang X, Bhattarai DP, Zhao Y-L, Sun B-F, Yang Y-G (2017) Cytoplasmic m6A reader YTHDF3 promotes mRNA translation. Cell Res 27:444–447 28106076 10.1038/cr.2017.10 PMC5339832

[CR31] Liao Y, Smyth GK, Shi W (2014) featureCounts: an efficient general purpose program for assigning sequence reads to genomic features. Bioinformatics 30:923–930 24227677 10.1093/bioinformatics/btt656

[CR32] Linder B, Grozhik AV, Olarerin-George AO, Meydan C, Mason CE, Jaffrey SR (2015) Single-nucleotide-resolution mapping of m6A and m6Am throughout the transcriptome. Nat Methods 12:767–772 26121403 10.1038/nmeth.3453 PMC4487409

[CR33] Liu C, Sun H, Yi Y, Shen W, Li K, Xiao Y, Li F, Li Y, Hou Y, Lu B, Liu W, Meng H, Peng J, Yi C, Wang J (2022) Absolute quantification of single-base m(6)A methylation in the mammalian transcriptome using GLORI. Nat Biotechnol 41(3):355–366 36302990 10.1038/s41587-022-01487-9

[CR34] Liu J, Dou X, Chen C, Chen C, Liu C, Xu MM, Zhao S, Shen B, Gao Y, Han D (2020b) N6-methyladenosine of chromosome-associated regulatory RNA regulates chromatin state and transcription. Science 367:580–586 31949099 10.1126/science.aay6018 PMC7213019

[CR35] Liu J, Gao M, He J, Wu K, Lin S, Jin L, Chen Y, Liu H, Shi J, Wang X et al (2021) The RNA m6A reader YTHDC1 silences retrotransposons and guards ES cell identity. Nature 591:322–326 33658714 10.1038/s41586-021-03313-9

[CR36] Liu J, Gao M, Xu S, Chen Y, Wu K, Liu H, Wang J, Yang X, Wang J, Liu W (2020a) YTHDF2/3 are required for somatic reprogramming through different RNA deadenylation pathways. Cell Rep 32:108120 32905781 10.1016/j.celrep.2020.108120

[CR37] Love MI, Huber W, Anders S (2014) Moderated estimation of fold change and dispersion for RNA-seq data with DESeq2. Genome Biol 15:1–21 10.1186/s13059-014-0550-8 PMC4302049 25516281

[CR38] Luo S, Tong L (2014) Molecular basis for the recognition of methylated adenines in RNA by the eukaryotic YTH domain. Proc Natl Acad Sci USA 111:13834–13839 25201973 10.1073/pnas.1412742111 PMC4183320

[CR39] Macfarlan TS, Gifford WD, Driscoll S, Lettieri K, Rowe HM, Bonanomi D, Firth A, Singer O, Trono D, Pfaff SL (2012) Embryonic stem cell potency fluctuates with endogenous retrovirus activity. Nature 487:57–63 22722858 10.1038/nature11244 PMC3395470

[CR40] Mangiavacchi A, Liu P, Della Valle F, Orlando V (2021) New insights into the functional role of retrotransposon dynamics in mammalian somatic cells. Cell Mol Life Sci 78:5245–5256 33990851 10.1007/s00018-021-03851-5 PMC8257530

[CR41] Mariner PD, Walters RD, Espinoza CA, Drullinger LF, Wagner SD, Kugel JF, Goodrich JA (2008) Human Alu RNA is a modular transacting repressor of mRNA transcription during heat shock. Mol Cell 29:499–509 18313387 10.1016/j.molcel.2007.12.013

[CR42] Martin M (2011) Cutadapt removes adapter sequences from high-throughput sequencing reads. EMBnet J 17:10–12

[CR43] Mu H, Zhang T, Yang Y, Zhang D, Gao J, Li J, Yue L, Gao D, Shi B, Han Y, Zhong L, Chen X, Wang Z-B, Lin Z, Tong M-H, Sun Q-Y, Yang Y-G, Han J (2021) METTL3-mediated mRNA N6-methyladenosine is required for oocyte and follicle development in mice. Cell Death Dis 12:989 34689175 10.1038/s41419-021-04272-9 PMC8542036

[CR44] Namekawa SH, Lee JT (2011) Detection of nascent RNA, single-copy DNA and protein localization by immunoFISH in mouse germ cells and preimplantation embryos. Nat Protoc 6:270–284 21372809 10.1038/nprot.2010.195 PMC4335666

[CR45] Park S-J, Komata M, Inoue F, Yamada K, Nakai K, Ohsugi M, Shirahige K (2013) Inferring the choreography of parental genomes during fertilization from ultralarge-scale whole-transcriptome analysis. Genes Dev 27:2736–2748 24352427 10.1101/gad.227926.113 PMC3877761

[CR46] Patil DP, Chen C-K, Pickering BF, Chow A, Jackson C, Guttman M, Jaffrey SRJN (2016) m6A RNA methylation promotes XIST-mediated transcriptional repression. Nature 537:369–373 27602518 10.1038/nature19342 PMC5509218

[CR47] Percharde M, Lin CJ, Yin Y, Guan J, Peixoto GA, Bulut-Karslioglu A, Biechele S, Huang B, Shen X, Ramalho-Santos M (2018) A LINE1-nucleolin partnership regulates early development and ESC identity. Cell 174:391–405.e19 29937225 10.1016/j.cell.2018.05.043 PMC6046266

[CR48] Picelli S, Faridani OR, Björklund ÅK, Winberg G, Sagasser S, Sandberg R (2014) Full-length RNA-seq from single cells using Smart-seq2. Nat Protoc 9:171–181 24385147 10.1038/nprot.2014.006

[CR49] Ponicsan S, Kugel J, Goodrich J (2015) Repression of RNA polymerase II transcription by B2 RNA depends on a specific pattern of structural regions in the RNA. Non-Coding RNA 1:4–16 26405685 10.3390/ncrna1010004 PMC4578731

[CR50] Ramírez F, Ryan DP, Grüning B, Bhardwaj V, Kilpert F, Richter AS, Heyne S, Dündar F, Manke T (2016) deepTools2: a next generation web server for deep-sequencing data analysis. Nucleic Acids Res 44:W160 27079975 10.1093/nar/gkw257 PMC4987876

[CR51] Ries RJ, Zaccara S, Klein P, Olarerin-George A, Namkoong S, Pickering BF, Patil DP, Kwak H, Lee JH, Jaffrey SR (2019) m6A enhances the phase separation potential of mRNA. Nature 571:424–428 31292544 10.1038/s41586-019-1374-1 PMC6662915

[CR52] Sakashita A, Kitano T, Ishizu H, Guo Y, Masuda H, Ariura M, Murano K, Siomi H (2023) Transcription of MERVL retrotransposons is required for preimplantation embryo development. Nat Genet 55:484–495 36864102 10.1038/s41588-023-01324-y PMC10011141

[CR53] Shen L, Liang Z, Yu HJB-P (2017) Dot blot analysis of N6-methyladenosine RNA modification levels. Bio-protocol 7:e2095 10.21769/BioProtoc.2095 PMC8376565 34458425

[CR54] Shi H, Wang X, Lu Z, Zhao BS, Ma H, Hsu PJ, Liu C, He C (2017) YTHDF3 facilitates translation and decay of N6-methyladenosine-modified RNA. Cell Res 27:315–328 28106072 10.1038/cr.2017.15 PMC5339834

[CR55] Shi H, Wei J, He C (2019) Where, when, and how: context-dependent functions of RNA methylation writers, readers, and erasers. Mol Cell 74:640–650 31100245 10.1016/j.molcel.2019.04.025 PMC6527355

[CR56] Sikorski V, Selberg S, Lalowski M, Karelson M, Kankuri E (2023) The structure and function of YTHDF epitranscriptomic m6A readers. Trends Pharmacol Sci 44:335–353 37069041 10.1016/j.tips.2023.03.004

[CR57] Su R, Fan L-H, Cao C, Wang L, Du Z, Cai Z, Ouyang Y-C, Wang Y, Zhou Q, Wu L (2021) Global profiling of RNA-binding protein target sites by LACE-seq. Nat Cell Biol 23:664–675 34108658 10.1038/s41556-021-00696-9

[CR58] Wang H, Dey SK (2006) Roadmap to embryo implantation: clues from mouse models. Nat Rev Genet 7:185–199 16485018 10.1038/nrg1808

[CR59] Wang J, Wang L, Diao J, Shi YG, Shi Y, Ma H, Shen H (2019) Binding to m6A RNA promotes YTHDF2-mediated phase separation. Protein Cell 11:304–307 10.1007/s13238-019-00660-2 PMC7093369 31642031

[CR60] Wang X, Lu Z, Gomez A, Hon GC, Yue Y, Han D, Fu Y, Parisien M, Dai Q, Jia G (2014) N6-methyladenosine-dependent regulation of messenger RNA stability. Nature 505:117–120 24284625 10.1038/nature12730 PMC3877715

[CR61] Wang X, Zhao BS, Roundtree IA, Lu Z, Han D, Ma H, Weng X, Chen K, Shi H, He C (2015) N6-methyladenosine modulates messenger RNA translation efficiency. Cell 161:1388–1399 26046440 10.1016/j.cell.2015.05.014 PMC4825696

[CR62] Wang Y, Li Y, Skuland T, Zhou C, Li A, Hashim A, Jermstad I, Khan S, Dalen KT, Greggains GD, Klungland A, Dahl JA, Au KF (2023) The RNA m6A landscape of mouse oocytes and preimplantation embryos. Nat Struct Mol Biol 30:703–709 37081317 10.1038/s41594-023-00969-x PMC10337017

[CR63] Wei J, Yu X, Yang L, Liu X, Gao B, Huang B, Dou X, Liu J, Zou Z, Cui X-L (2022) FTO mediates LINE1 m6A demethylation and chromatin regulation in mESCs and mouse development. Science 376:968–973 35511947 10.1126/science.abe9582 PMC9746489

[CR64] Williams LH, Fromm G, Gokey NG, Henriques T, Muse GW, Burkholder A, Fargo DC, Hu G, Adelman K (2015) Pausing of RNA polymerase II regulates mammalian developmental potential through control of signaling networks. Mol Cell 58:311–322 25773599 10.1016/j.molcel.2015.02.003 PMC4402150

[CR65] Wu Y, Xu X, Qi M, Chen C, Li M, Yan R, Kou X, Zhao Y, Liu W, Li Y, Liu X, Zhang M, Yi C, Liu H, Xiang J, Wang H, Shen B, Gao Y, Gao S (2022) N6-methyladenosine regulates maternal RNA maintenance in oocytes and timely RNA decay during mouse maternal-to-zygotic transition. Nat Cell Biol 24:917–927 35606490 10.1038/s41556-022-00915-x

[CR66] Xiong Z, Xu K, Lin Z, Kong F, Wang Q, Quan Y, Sha Q-Q, Li F, Zou Z, Liu L et al (2022) Ultrasensitive Ribo-seq reveals translational landscapes during mammalian oocyte-to-embryo transition and pre-implantation development. Nat Cell Biol 24:968–980 35697785 10.1038/s41556-022-00928-6

[CR67] Xu W, Li J, He C, Wen J, Ma H, Rong B, Diao J, Wang L, Wang J, Wu F, Tan L, Shi YG, Shi Y, Shen H (2021) METTL3 regulates heterochromatin in mouse embryonic stem cells. Nature 591:317–321 33505026 10.1038/s41586-021-03210-1

[CR68] Yao H, Gao CC, Zhang D, Xu J, Song G, Fan X, Liang DB, Chen YS, Li Q, Guo Y, Cai YT, Hu L, Zhao YL, Sun YP, Yang Y, Han J, Yang YG (2023) scm6A-seq reveals single-cell landscapes of the dynamic m(6)A during oocyte maturation and early embryonic development. Nat Commun 14:315 36658155 10.1038/s41467-023-35958-7 PMC9852475

[CR69] Yu G, Wang L-G, He Q-Y (2015) ChIPseeker: an R/Bioconductor package for ChIP peak annotation, comparison and visualization. Bioinformatics 31:2382–2383 25765347 10.1093/bioinformatics/btv145

[CR70] Zaccara S, Jaffrey SR (2020) A unified model for the function of YTHDF proteins in regulating m6A-modified mRNA. Cell 181:1582–1595.e18 32492408 10.1016/j.cell.2020.05.012 PMC7508256

[CR71] Zhang X-O, Gingeras TR, Weng Z (2019) Genome-wide analysis of polymerase III-transcribed Alu elements suggests cell-type-specific enhancer function. Genome Res 29:1402–1414 31413151 10.1101/gr.249789.119 PMC6724667

[CR72] Zhang Y, Liu T, Meyer CA, Eeckhoute J, Johnson DS, Bernstein BE, Nusbaum C, Myers RM, Brown M, Li W (2008) Model-based analysis of ChIP-Seq (MACS). Genome Biol 9:1–9 10.1186/gb-2008-9-9-r137 PMC2592715 18798982

[CR73] Zhu W, Ding Y, Meng J, Gu L, Liu W, Li L, Chen H, Wang Y, Li Z, Li C, Sun Y, Liu Z (2023) Reading and writing of mRNA m6A modification orchestrate maternal-to-zygotic transition in mice. Genome Biol 24(1):67 37024923 10.1186/s13059-023-02918-9 PMC10080794

[CR74] Zou Z, Sepich-Poore C, Zhou X, Wei J, He C (2023) The mechanism underlying redundant functions of the YTHDF proteins. Genome Biol 24:17 36694229 10.1186/s13059-023-02862-8 PMC9872407

[CR75] Zovoilis A, Cifuentes-Rojas C, Chu H-P, Hernandez AJ, Lee JT (2016) Destabilization of B2 RNA by EZH2 activates the stress response. Cell 167:1788–1802.e13 27984727 10.1016/j.cell.2016.11.041 PMC5552366

[CR76] Zuo E, Cai Y-J, Li K, Wei Y, Wang B-A, Sun Y, Liu Z, Liu J, Hu X, Wei W (2017) One-step generation of complete gene knockout mice and monkeys by CRISPR/Cas9-mediated gene editing with multiple sgRNAs. Cell Res 27:933–945 28585534 10.1038/cr.2017.81 PMC5518993

