# Supplementary material for: m6A reader Ythdf proteins control retrotransposon B2 repeat expression and safeguard early embryo development
Source: EMBO J. 2026 Mar 11;45(8):2494–522. doi: 10.1038/s44318-026-00728-w (PMC13083883; doi:10.1038/s44318-026-00728-w)
Supplement: Supplementary file 4 — Appendix [file 44318_2026_728_MOESM4_ESM.pdf]

Appendix for "m6A reader Ythdf proteins control retrotransposon B2 repeat expression and safeguard early embryo development"

Table of contents

Appendix Figure S1 2

Appendix Figure S2 4

Appendix Figure S3 6

Appendix Figure S4 7

Appendix Figure S5 9

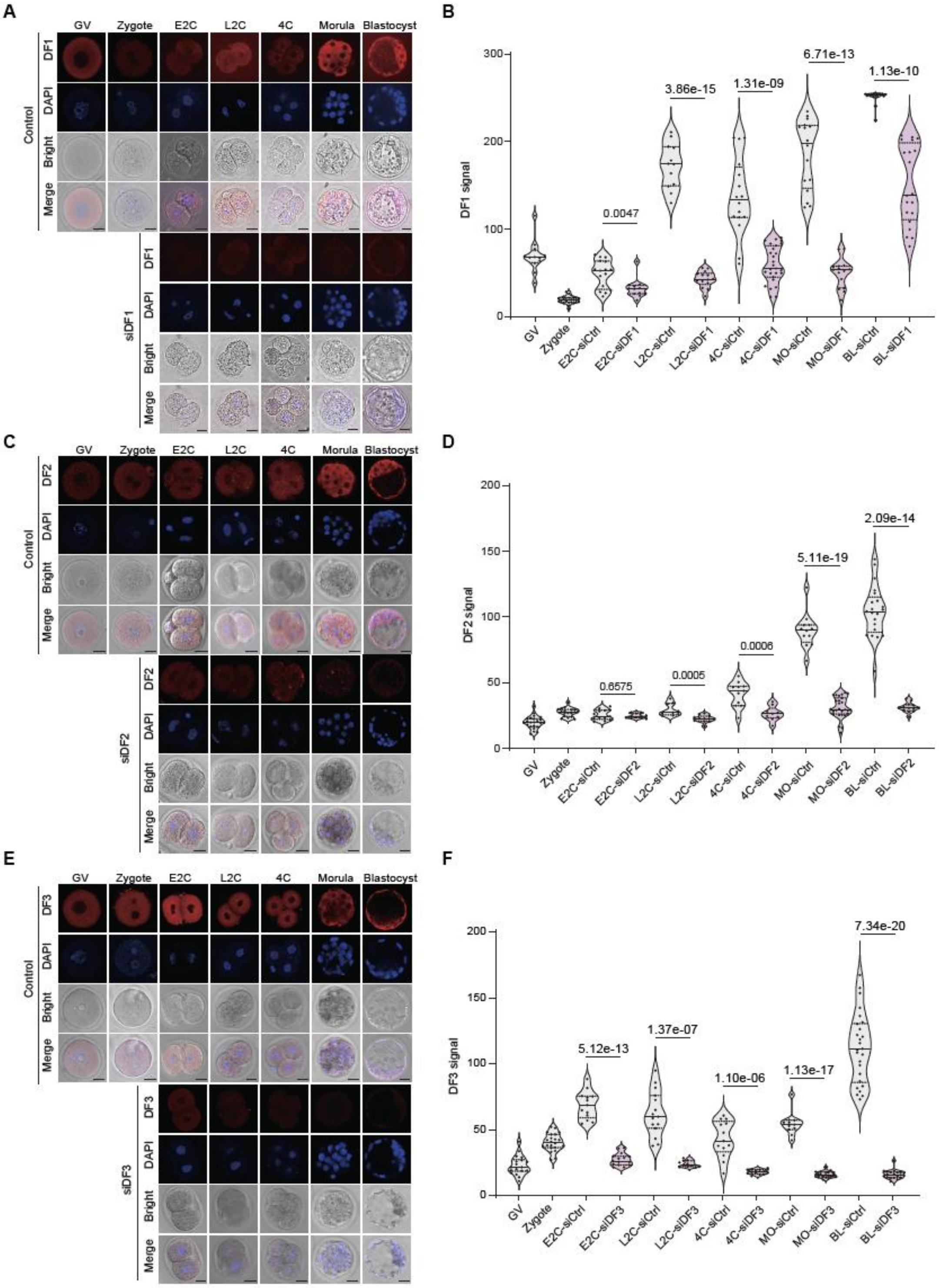

Appendix Figure S1. Validation of Ythdf knockdown efficiency across preimplantation developmental stages using immunofluorescence.

(**A**) Representative images of immunofluorescence staining of DF1 with DAPI counterstain in control and si*Ythdf1* knockdown embryos and wild-type (WT) oocytes. Scale bar, 20 µm. (**B**) Violin plots showing the DF1 intensity in control and si*Ythdf1* knockdown embryos and WT oocytes. GV oocyte, *n*=12; Zygote, *n*=24; E2C-siCtrl, *n*=16; E2C-siDF1, *n*=12; L2C-siCtrl, *n*=12; L2C-siDF1, *n*=14;

4C-siCtrl, *n*=16;4C-siDF1, *n*=27; MO-siCtrl, *n*=16; MO-siDF1, *n*=15; BL-siCtrl, *n*=17; BL-siDF1, *n*=20. (**C**) Representative images of immunofluorescence staining of DF2 with DAPI counterstain in control and si*Ythdf2* knockdown embryos and WT oocytes. Scale bar, 20 µm. (**D**) Violin plots showing the DF2 intensity in control and si*Ythdf2* knockdown embryos and WT oocytes. GV oocyte, *n*=23; Zygote, *n*=18; E2C-siCtrl, *n*=13; E2C-siDF2, *n*=9; L2C-siCtrl, *n*=9; L2C-siDF2, *n*=12; 4C-siCtrl, *n*=11;4C-siDF2, *n*=9; MO-siCtrl, *n*=12; MO-siDF2, *n*=27; BL-siCtrl, *n*=21; BL-siDF2, *n*=13. (**E**) Representative images of immunofluorescence staining of DF3 with DAPI counterstain in control and si*Ythdf3* knockdown embryos and WT oocytes. Scale bar, 20 µm. (**F**) Violin plots showing the DF3 intensity in control and si*Ythdf3* knockdown embryos and WT oocytes. GV oocyte, *n*=18; Zygote, *n*=22; E2C-siCtrl, *n*=13; E2C-siDF3, *n*=15; L2C-siCtrl, *n*=15; L2C-siDF3, *n*=11; 4C-siCtrl, *n*=13;4C-siDF3, *n*=13; MO-siCtrl, *n*=11; MO-siDF3, *n*=20; BL-siCtrl, *n*=26; BL-siDF3, *n*=20.The *P*-values in (**B**), (**D**), and (**F**) were determined by a two-tailed unpaired *t*-test.

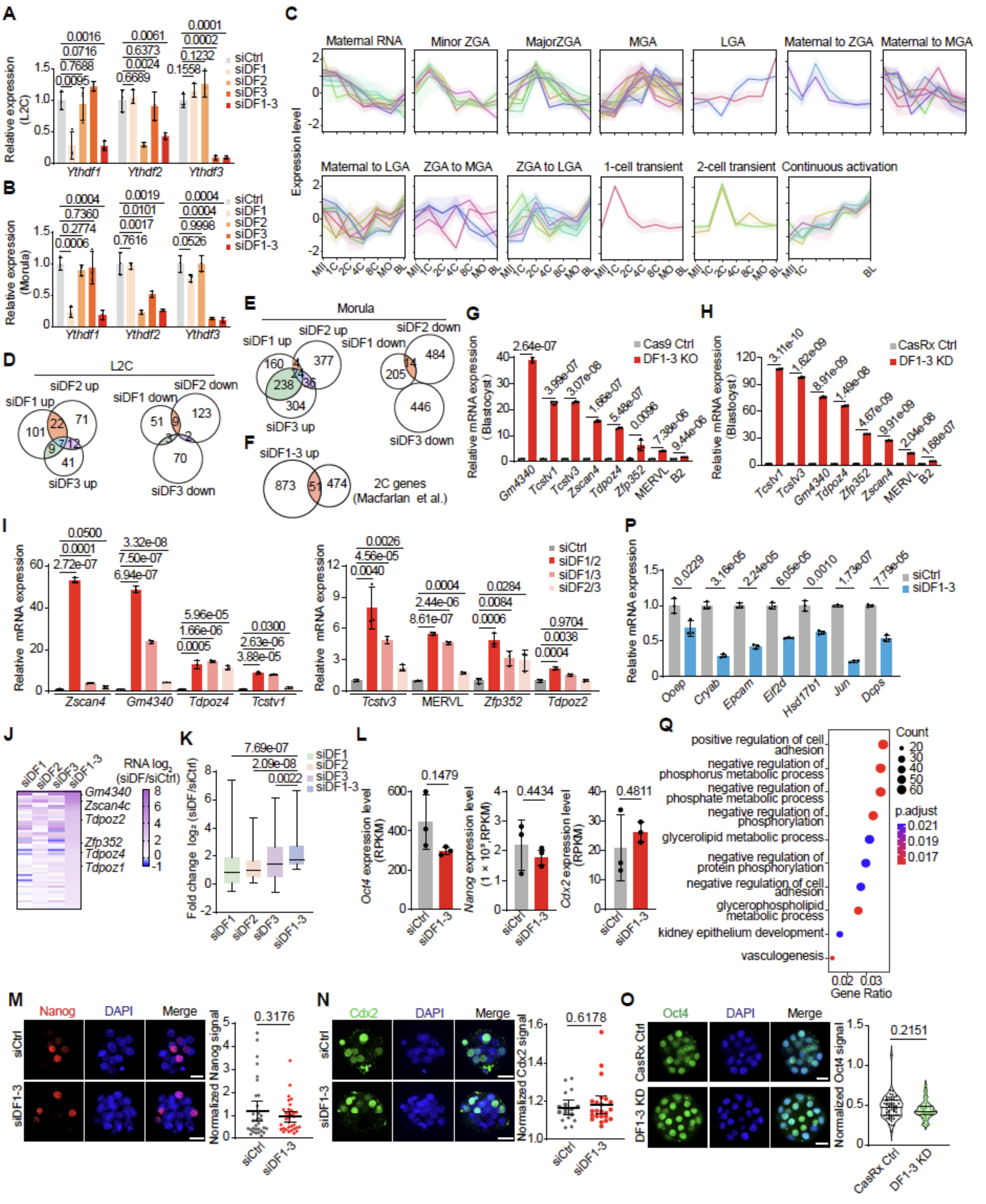

Appendix Figure S2. Knockdown of *Ythdf* perturbs the transcriptome of the embryos.

(**A, B**) Relative expression of *Ythdf1/2/3* from single DF or triple DF KD RNA-seq data in the L2C (**A**) and morula (**B**) embryos. Data are mean ± SD, *n* = 3 biological replicates. (**C**) Gene expression patterns were identified by developmental transcriptome data from a previous publication. (**D**, **E**) Venn diagram showing the overlap of DEGs identified in si*Ythdf* RNA-seq data at the L2C (**D**) and morula (**E**) stages. (**F**) Venn diagram showing the overlap of upregulated genes identified in si*Ythdf1-3* RNA-seq data and previously

reported 2C genes. (**G**, **H**) RT-qPCR validation of upregulation of 2C genes in Cas9-mediated DF1-3 KO (**G**) and CasRx-mediated DF1-3 KD (**H**) blastocysts. (**I**) RT-qPCR validation of upregulation of 2C genes in siRNA-mediated double *Ythdf* knockdown blastocysts. (**J**) Heatmap showing the upregulated 2C genes in si*Ythdf1-3* knockdown morula embryos and corresponding expression changes in si*Ythdf*1, si*Ythdf*2, and si*Ythdf*3 morula embryos. (**K**) Boxplots showing the expression levels of 2C marker genes (n=51) in si*Ythdf*1, si*Ythdf*2, si*Ythdf*3, and si*Ythdf1*-3 morula embryos. The boxplots display the 25th–75th percentiles with the median indicated by a central line; whiskers extend to the minimum and maximum values. *P*-values were calculated by a two-tailed paired *t*-test. (**L**) Bar chart showing gene expression from si*Ythdf1-3* and siControl RNA-seq data. (**M**, **N**) Representative images of immunofluorescence staining for Nanog (**M**) and Cdx2 (**N**), with DAPI counterstain, in control and si*Ythdf1-3* knockdown morula at 3.5 dpc. Scale bars, 20 μm. Dot plots showing the relative intensity of Nanog (siCtrl, *n*=32; siDF1-3, *n*=33) and Cdx2 (siCtrl, *n*=17; siDF1-3, *n*=23) in control and si*Ythdf1-3* knockdown morula at 3.5 dpc, normalized to DAPI signal. Each error bar represents the mean ± SEM. (**O**) Representative images of immunofluorescence staining for Oct4 with DAPI counterstain, in CasRx control and CasRx-mediated *Ythdf1-3* KD morula at 3.5 dpc. Scale bars, 20 μm. Violin plots showing the relative intensity of Oct4 in CasRx control and CasRx-mediated *Ythdf1-3* KD morula at 3.5 dpc, normalized to DAPI signal. The upper and lower dotted lines in the violin plots represent upper and lower quartiles (25th and 75th percentiles), and the center line represents the median. (**P**) RT-qPCR validation of downregulated genes in si*Ythdf1-3* knockdown morula. Data in (**G**), (**H**), (**I**), (**L**), and (**P**) are mean ± SD, n = 3 biological replicates. *P*-values in (**A**), (**B**), (**G**), (**H**), (**I**), and (**L**-**P**) were calculated by a two-tailed unpaired *t*-test. (**Q**) GO analysis of DEGs from si*Ythdf1-3* knockdown morula compared to control. The *P*-value was determined by Fisher's exact test.

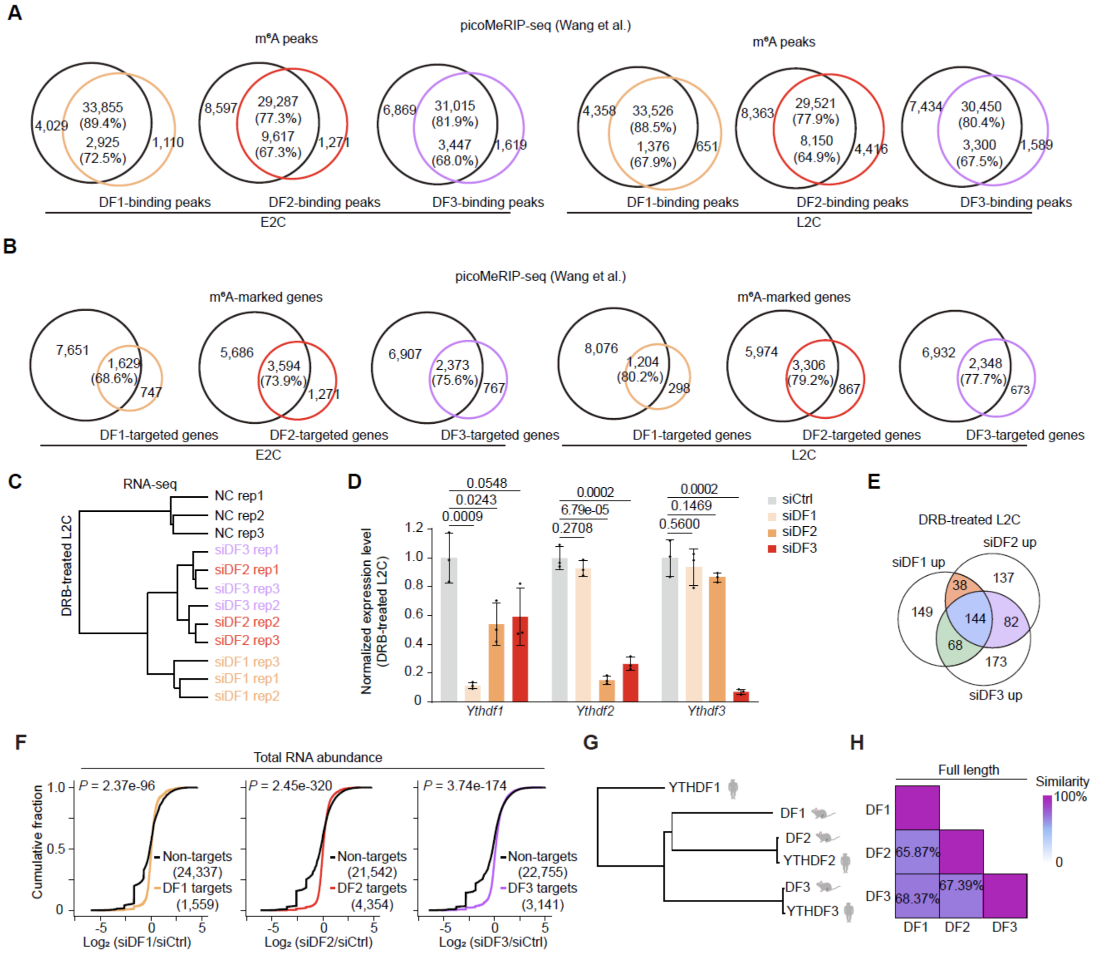

Appendix Figure S3. Each DF1/2/3 protein promotes RNA decay in embryos.

(**A**) Venn diagram showing the overlap between m^{6}A peaks identified from picoMeRIP-seq datasets and DF binding peaks identified from LACE-seq datasets. Both the m^{6}A peaks and the DF binding peaks are from non-intronic transcripts. (**B**) Venn diagram showing the overlap between m^{6}A-containing genes identified from picoMeRIP-seq datasets and YTHDF binding genes identified from LACE-seq datasets. (**C**) Hierarchical clustering for si*Ythdf1/2/3* RNA-seq in DRB-treated L2C embryos. (**D**) Relative expression levels of *Ythdf1/2/3* from RNA-seq datasets of single DF KD L2C embryos following DRB treatment. Data are mean ± SD, n = 3 biological replicates. The *P*-value was determined by a two-tailed unpaired t-test. (**E**) Venn diagram showing the overlap of upregulated genes identified in si*Ythdf1/2/3* RNA-seq datasets in the DRB-treated L2C embryos. (**F**) Cumulative distributions of the $\log_2$ fold changes in RNA abundance between si*Ythdf* and siControl for non-targets and DF LACE-seq targets at the L2C stages. *P*-values were determined by the Kolmogorov-Smirnov test. (**G**) Hierarchical clustering showing conservation of human and mouse DF proteins. (**H**) Heatmap showing the similarity scores calculated by the BLOSUM62 algorithm of DF1/2/3 full-length proteins.

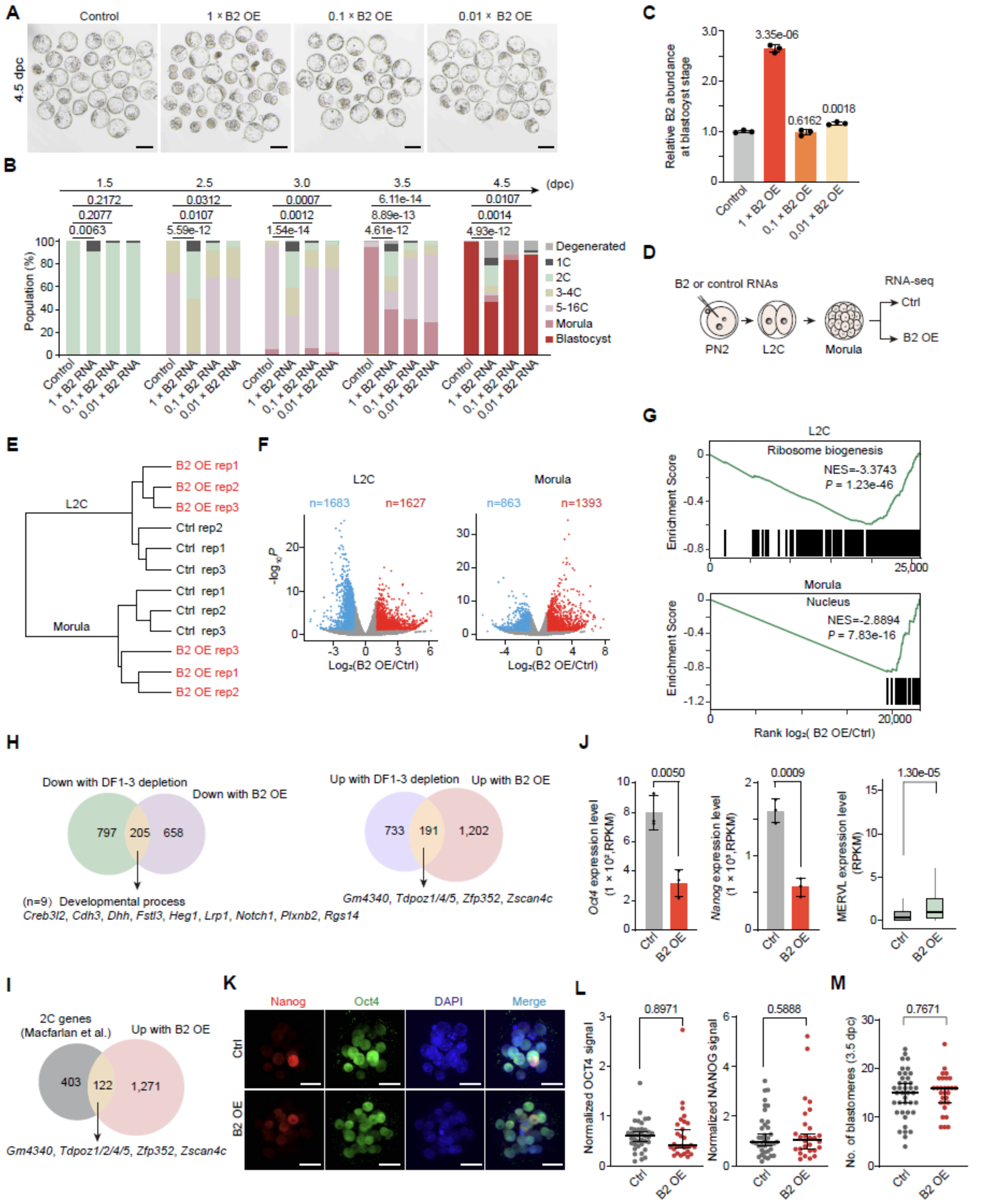

Appendix Figure S4. Overexpressing B2 RNAs perturbs the embryonic transcriptome.

(**A**) Representative images showing control, 1×B2 OE (overexpression), 0.1×B2 OE, and 0.01×B2 OE embryos microinjected with B2 RNA or DMSO at 4.5 dpc. Representative images were selected from two independent experiments. **(B**) Percentage of embryonic stages observed at the indicated time points under control (*n* = 74), 1×B2 OE (*n* = 73), 0.1×B2 OE (*n* = 47), and 0.01×B2 OE (*n* = 49) conditions. Scale bars, 100 μm. *P*-values were determined by the Chi-square test. **(C**) RT-qPCR validation of B2 RNA levels in blastocysts microinjected with a gradient of B2 RNAs. Data are mean ± SD, n = 3 biological replicates. (**D**) Schematic of the experimental procedure for performing RNA-

seq of B2 OE embryos at L2C and morula. (**E**) Hierarchical clustering for B2 OE in the L2C and morula embryos. (**F**) Volcano plot showing gene expression changes upon B2 OE at L2C and morula stage (3 biological replicates for each B2 overexpression condition). The *P*-values were determined using DESeq2, with a threshold of 0.05. (**G**) GSEA plot showing preferential downregulation of the ribosome biogenesis pathway (top) and the nucleus pathway (bottom) from B2 OE RNA-seq in the L2C and morula embryos. The *P*-values were determined by the hypergeometric test. (**H**) Venn diagram showing the overlap of downregulated (left) and upregulated (right) genes identified from B2 OE and *Ythdf1-3* KD morula embryos. (**I**) Venn diagram showing the overlap of upregulated genes identified from B2 OE RNA-seq datasets and previously reported 2C genes. (**J**) Bar chart and box plot showing gene and MERVL expression from B2 OE and control RNA-seq data in the morulae. Data in the Bar chart are mean ± SD, n = 3 biological replicates. The *P*-values in the bar chart were determined by a two-tailed unpaired *t*-test. MERVL, n=8307. Boxes represent the 25th-75th percentile (line at the median), with whiskers at 1.5 × interquartile range (IQR). The *P*-values in the box plot were determined by a two-tailed paired *t*-test. (**K**) Representative images of immunofluorescence staining for Oct4 and Nanog, with DAPI counterstain, in control and B2 OE embryos at 3.5 dpc. Scale bars, 20 μm. (**L**) Dot plots showing the relative intensity of Oct4 (left) and Nanog (right) in control and B2 OE embryos at 3.5 dpc, normalized to DAPI signal. Each error bar represents the mean ± SEM. (**M**) Dot plots showing the total number of blastomeres per embryo in control and B2 OE embryos at 3.5 dpc. The error bar represents the mean ± SEM. The *P*-values in (**C**), (**L**), and (**M**) were determined by a two-tailed unpaired *t*-test.

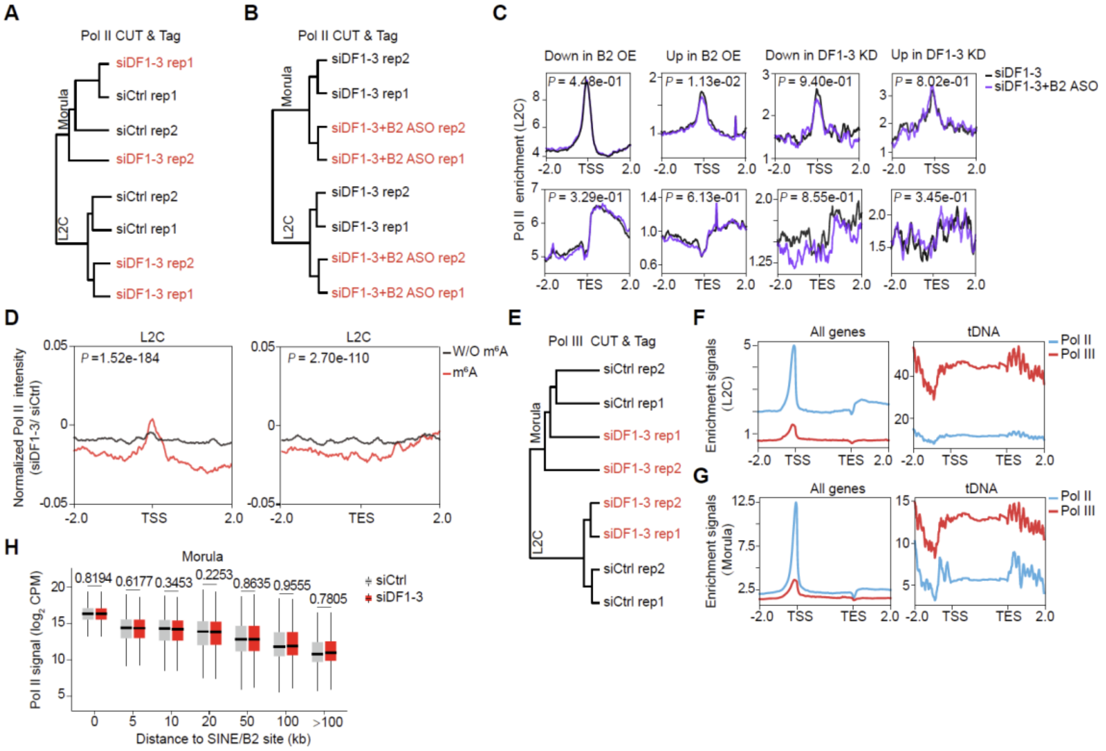

Appendix Figure S5. Profiling of Pol II and Pol III binding in *Ythdf1-3* knockdown embryos.

(**A**) Hierarchical clustering for Pol II CUT&Tag upon si*Ythdf1-3* knockdown and control embryos at the L2C and morula stages. (**B**) Hierarchical clustering for Pol Ⅱ CUT&Tag upon si*Ythdf1-3* knockdown and B2 ASO rescue in the L2C and morula embryos. **(C**) Profiles of Pol II levels on DEG loci of B2 OE (left) and si*Ythdf1-3* knockdown (right) from 2.0 kb up- to down-stream of the TSS (upper) or the TES (bottom) in si*Ythdf1-3* knockdown and B2 ASO rescue embryos at the L2C stage. *P*-values were determined by the Kolmogorov-Smirnov test. (**D**) Profiles of Pol II levels on gene loci of m^6A-marked and non-m^6A-marked from 2.0 kb up to downstream of the TSS or the TES in *Ythdf1-3* knockdown embryos related to control embryos at the L2C stage. *P*-values were determined by the Kolmogorov-Smirnov test. (**E**) Hierarchical clustering for Pol III CUT&Tag upon si*Ythdf1-3* knockdown and control embryos at the L2C and morula stages. (**F, G**) Profiles of Pol II and Pol III binding on all gene loci (left) and tDNA loci (right) from 2.0 kb upstream of the TSS to 2.0 kb downstream of the TES in the L2C (**F**) and morula (**G**) embryos. (**H**) Box plots showing Pol II signal fold changes between si*Ythdf1-3* knockdown and control embryos at the morula stage. The Pol II signals were categorized according to their genomic distance to the nearest B2 loci. B2 loci (0 kb), n=8,649; B2 loci (0-5 kb), n=5,448; B2 loci (5-10 kb), n=3,442; B2 loci (10-20 kb), n=3,968; B2 loci (20-50 kb), n=5,336; B2 loci (50-100 kb), n=3,922; B2 loci (>100 kb), n=5,944. Boxes represent the 25th-75th percentile (line at the median), with whiskers at 1.5 × interquartile range (IQR). *P*-values were determined by the Kolmogorov-Smirnov test.
