# Supplementary material for: m6A reader Ythdf proteins control retrotransposon B2 repeat expression and safeguard early embryo development
Source: EMBO J. 2026 Mar 11;45(8):2494–522. doi: 10.1038/s44318-026-00728-w (PMC13083883; doi:10.1038/s44318-026-00728-w)
Supplement: Supplementary file 18 — Expanded View Figures [file 44318_2026_728_MOESM18_ESM.pdf]

Expanded View Figures

Figure EV1. Associated with Fig. 1.

(**A**) RT-qPCR validation of *Ythdf* knockdown in L2C embryos. #1#2#3, three different *Ythdf* siRNAs. Data are mean ± SD, $n = 3$ biological replicates. (**B**, **C**) Western blotting showing the knockdown efficiency of *Ythdf* in L2C (**B**) and morula (**C**) embryos. (**D**) Relative expression of *Ythdf1/2/3* measured by RT-qPCR following single, double, or triple DF knockdown in E2C and L2C embryos. Data are mean ± SD, $n = 3$ biological replicates. (**E**) Simple Western immunoblotting assays showing DF1/2/3 protein levels when single DF, double DF, or triple DF knockdown in the E2C and L2C embryos. (**F–H**) Representative images of immunofluorescence staining of DF1 (**F**), DF2 (**G**), and DF3 (**H**) with DAPI counterstain in Cas9 control and DF1-3 KO morula at 3.5 dpc. Scale bars, 20 µm. Violin plots showing the DF1 (Control, $n = 31$; DF1-3 KO, $n = 27$), DF2 (Control, $n = 30$; DF1-3 KO, $n = 23$), and DF3 (Control, $n = 25$; DF1-3 KO, $n = 29$) intensity in Cas9 control and DF1-3 KO morula at 3.5 dpc. The upper and lower dotted lines in the violin plots represent upper and lower quartiles (25th and 75th percentiles), and the center line represents the median. (**I**) Representative images showing embryos treated with *Ythdf* guide RNAs (CasRx) or control guide RNA at 4.5 dpc. Representative images were selected from two independent experiments. Scale bars, 100 µm. (**J**) Percentages of embryonic stages observed at the 4.5 dpc in CasRx Control (CasRx Ctrl, $n = 28$), DF1 KD ($n = 28$), DF2 KD ($n = 27$), DF3 KD ($n = 54$), and DF1-3 KD ($n = 28$) groups. (**K**) Relative expression of *Ythdf1/2/3* measured by RT-qPCR following single, double, or triple DF knockdown in L2C embryos. Data are mean ± SD, $n = 3$ biological replicates. (**L–N**) Representative images of immunofluorescence staining of DF1 (**L**), DF2 (**M**), and DF3 (**N**) with DAPI counterstain in CasRx control and DF1-3 KD morula at 3.5 dpc. Scale bars, 20 µm. Violin plots showing the DF1 (Control, $n = 33$; DF1-3 KD, $n = 21$), DF2 (Control, $n = 30$; DF1-3 KD, $n = 17$), and DF3 (Control, $n = 18$; DF1-3 KD, $n = 21$) intensity in CasRx control and DF1-3 KD morula at 3.5 dpc. The upper and lower dotted lines in the violin plots represent upper and lower quartiles (25th and 75th percentiles), and the center line represents the median. (**O**) Representative images showing embryos treated with *Ythdf* siRNAs, *Ythdf* mRNA, or control siRNA at 4.5 dpc. Representative images were selected from 2 to 4 independent experiments. Scale bars, 100 µm. (**P**) Percentages of embryonic stages observed at the indicated time points in siCtrl ($n = 128$), siDF1/2 ($n = 55$), siDF1/2 + DF3 ($n = 30$), siDF1/3 ($n = 57$), siDF1/3 + DF2 ($n = 36$), siDF2/3 ($n = 96$), and siDF2/3 + DF1($n = 81$) conditions. *P* values in (**J**, **P**) were determined by the Chi-square test. (**Q**) RT-qPCR validation of *Ythdf* mRNA in blastocysts with double *Ythdf* knockdown and another *Ythdf* overexpression. Data in (**A**, **E**) are mean ± SD, $n = 3$ biological replicates. The *P* value in (**A**, **D**, **F**, **G**, **H**, **K**, **L**, **M**, **N**, **Q**) was determined by a two-tailed unpaired *t* test. Source data are available online for this figure.

 © The Author(s)

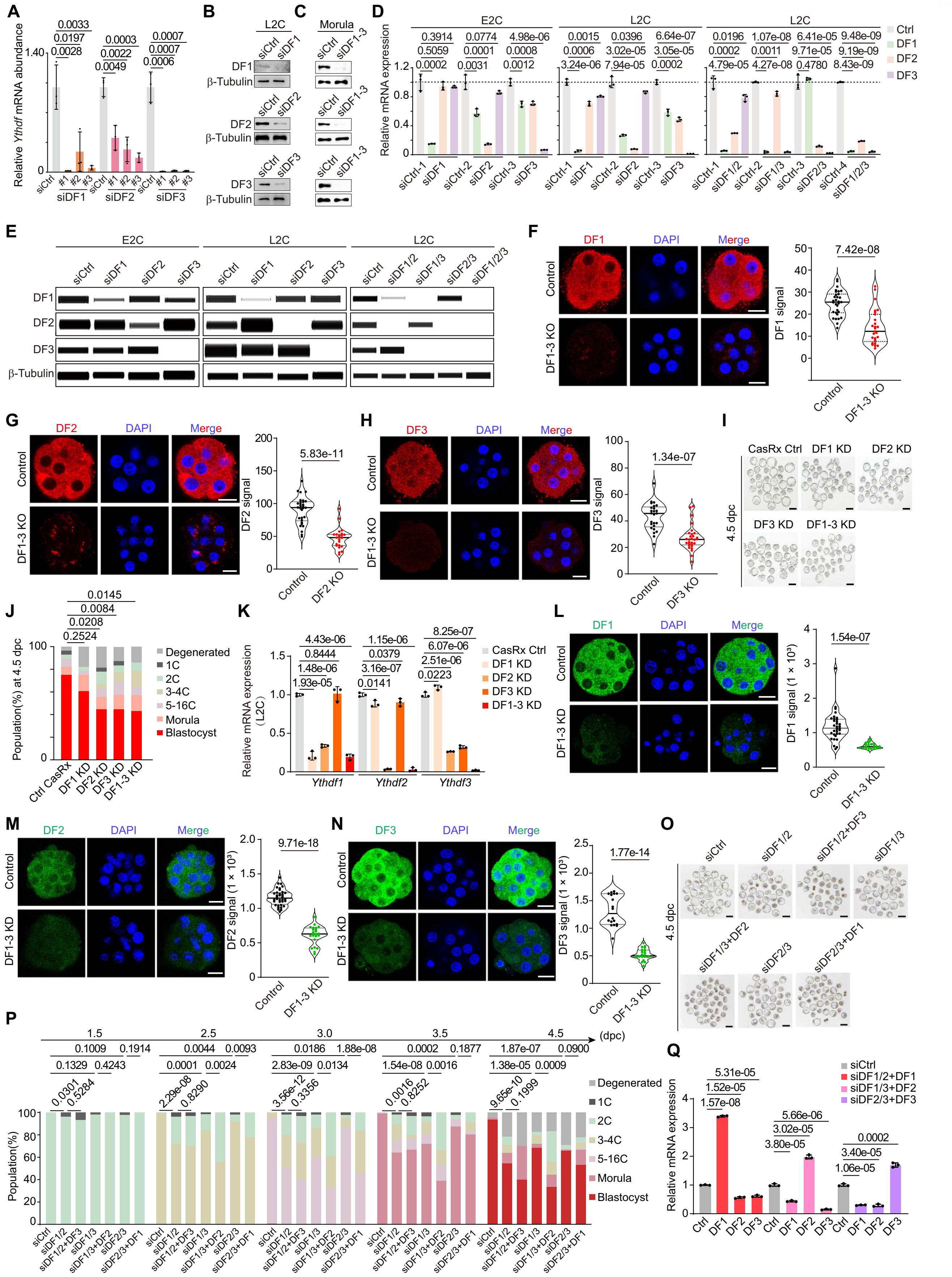
A
Relative Ythdf mRNA abundance
0.0033
0.0197
0.0028
0.0003
0.0022
0.0049
0.0007
0.0007
0.0006
siCtrl #1 #2 #3 siCtrl #1 #2 #3 siCtrl #1 #2 #3
siDF1 siDF2 siDF3
B
L2C
siCtrl siDF1
DF1
β-Tubulin
siCtrl siDF2
DF2
β-Tubulin
siCtrl siDF3
DF3
β-Tubulin
C
Morula
siCtrl siDF1-3
D
Relative mRNA expression
E2C
L2C
L2C
0.3914 0.0774 4.98e-06
0.5059 0.0001 0.0008
0.0002 0.0031 0.0012
0.0015 0.0396 6.64e-07
0.0006 3.02e-05 3.05e-05
3.24e-06 7.94e-05 0.0002
0.0196 1.07e-08 6.41e-05 9.48e-09
0.0002 0.0011 9.71e-05 9.19e-09
4.79e-05 4.27e-08 0.4780 8.43e-09
Ctrl
DF1
DF2
DF3
siCtrl-1 siDF1 siCtrl-2 siDF2 siCtrl-3 siDF3
siCtrl-1 siDF1/2 siCtrl-2 siDF1/3 siCtrl-3 siDF2/3 siCtrl-4 siDF1/2/3
E
E2C
L2C
L2C
siCtrl siDF1 siDF2 siDF3
siCtrl siDF1/2 siDF1/3 siDF2/3 siDF1/2/3
DF1
DF2
DF3
β-Tubulin
F
DF1 DAPI Merge
Control
DF1-3 KO
DF1 signal
7.42e-08
Control DF1-3 KO
G
DF2 DAPI Merge
Control
DF1-3 KO
DF2 signal
5.83e-11
Control DF2 KO
H
DF3 DAPI Merge
Control
DF1-3 KO
DF3 signal
1.34e-07
Control DF3 KO
I
CasRx Ctrl DF1 KD DF2 KD
DF3 KD DF1-3 KD
4.5 dpc
J
Population(%) at 4.5 dpc
0.0145
0.0084
0.0208
0.2524
Degenerated
1C
2C
3-4C
5-16C
Morula
Blastocyst
Ctrl CasRx DF1 KD DF2 KD DF3 KD DF1-3 KD
K
Relative mRNA expression (L2C)
4.43e-06
0.8444
1.48e-06
1.93e-05
1.15e-06
0.0379
3.16e-07
0.0141
8.25e-07
6.07e-06
2.51e-06
0.0223
CasRx Ctrl
DF1 KD
DF2 KD
DF3 KD
DF1-3 KD
Ythdf1 Ythdf2 Ythdf3
L
DF1 DAPI Merge
Control
DF1-3 KD
DF1 signal (1 × 10³)
1.54e-07
Control DF1-3 KD
M
DF2 DAPI Merge
Control
DF1-3 KD
DF2 signal (1 × 10³)
9.71e-18
Control DF1-3 KD
N
DF3 DAPI Merge
Control
DF1-3 KD
DF3 signal (1 × 10³)
1.77e-14
Control DF1-3 KD
O
siCtrl siDF1/2 siDF1/2+DF3 siDF1/3
siDF1/3+DF2 siDF2/3 siDF2/3+DF1
4.5 dpc
P
1.5 2.5 3.0 3.5 4.5 (dpc)
0.1009 0.1914
0.1329 0.4243
0.0301 0.5284
0.0044 0.0093
0.0001 0.0024
2.29e-08 0.8290
0.0186 1.88e-08
2.83e-09 0.0134
3.56e-12 0.3356
0.0002 0.1877
1.54e-08 0.0016
0.0016 0.8252
1.87e-07 0.0900
1.38e-05 0.0009
9.65e-10 0.1999
Population(%)
Degenerated
1C
2C
3-4C
5-16C
Morula
Blastocyst
siCtrl siDF1/2 siDF1/2+DF3 siDF1/3 siDF1/3+DF2 siDF2/3 siDF2/3+DF1
Q
Relative mRNA expression
5.31e-05
1.52e-05
1.57e-08
5.66e-06
3.02e-05
3.80e-05
0.0002
3.40e-05
1.06e-05
siCtrl
siDF1/2+DF1
siDF1/3+DF2
siDF2/3+DF3
Ctrl DF1 DF2 DF3 Ctrl DF1 DF2 DF3 Ctrl DF1 DF2 DF3

© The Author(s)

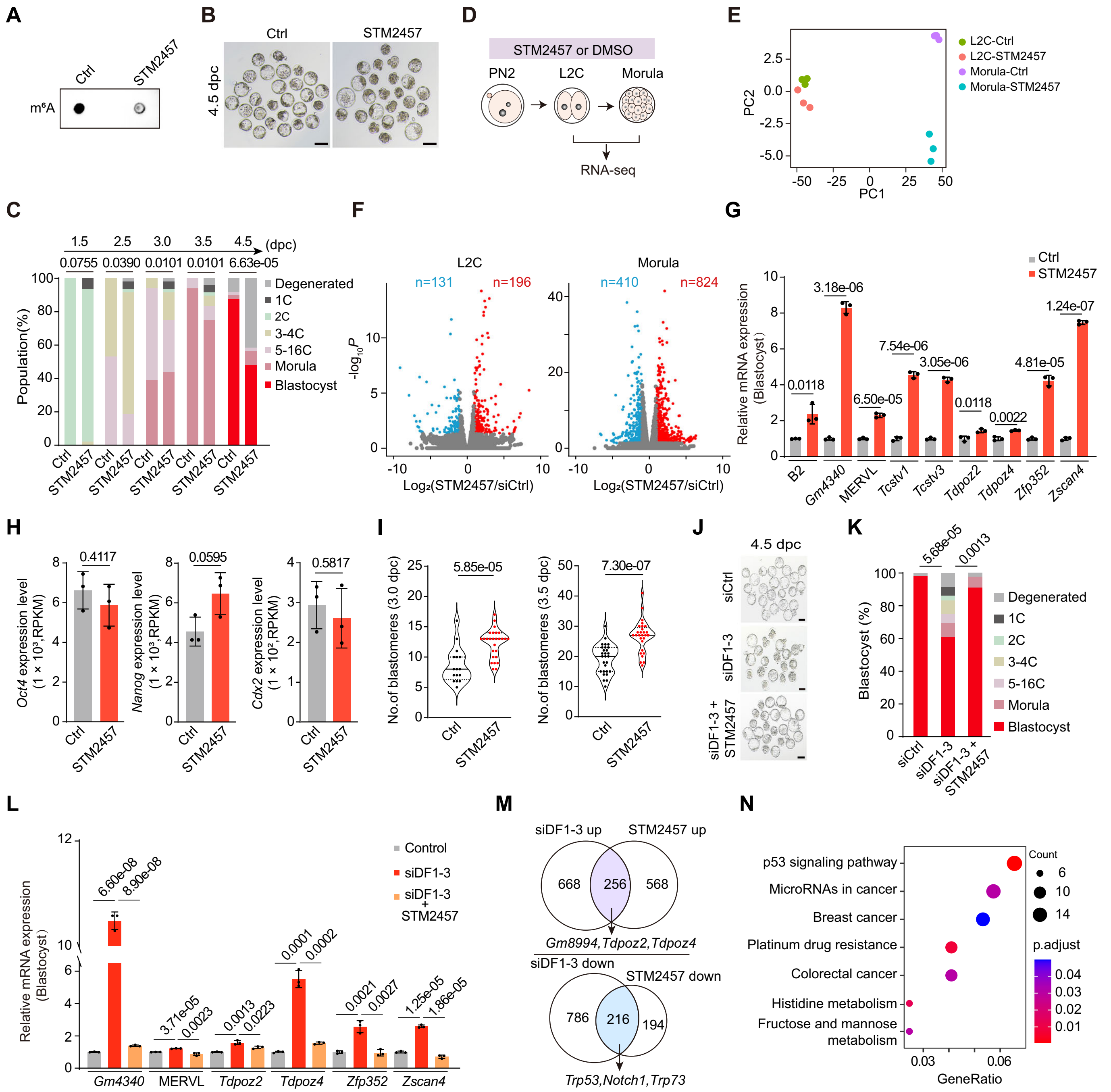
A
Ctrl
STM2457
m6A
B
Ctrl
STM2457
4.5 dpc
D
STM2457 or DMSO
PN2
L2C
Morula
RNA-seq
E
L2C-Ctrl
L2C-STM2457
Morula-Ctrl
Morula-STM2457
PC2
PC1
C
1.5 2.5 3.0 3.5 4.5 (dpc)
0.0755 0.0390 0.0101 0.0101 6.63e-05
Degenerated
1C
2C
3-4C
5-16C
Morula
Blastocyst
Population(%)
Ctrl
STM2457
F
L2C
n=131
n=196
Morula
n=410
n=824
-log10P
Log2(STM2457/siCtrl)
G
Ctrl
STM2457
Relative mRNA expression (Blastocyst)
0.0118
3.18e-06
6.50e-05
7.54e-06
3.05e-06
0.0118
0.0022
4.81e-05
1.24e-07
B2
Gm4340
MERVL
Tcstv1
Tcstv3
Tdpoz2
Tdpoz4
Zfp352
Zscan4
H
0.4117
0.0595
0.5817
Oct4 expression level (1 × 10³,RPKM)
Nanog expression level (1 × 10³,RPKM)
Cdx2 expression level (1 × 10²,RPKM)
Ctrl
STM2457
I
5.85e-05
7.30e-07
No.of blastomeres (3.0 dpc)
No.of blastomeres (3.5 dpc)
Ctrl
STM2457
J
4.5 dpc
siCtrl
siDF1-3
siDF1-3 + STM2457
K
5.68e-05
0.0013
Blastocyst (%)
Degenerated
1C
2C
3-4C
5-16C
Morula
Blastocyst
siCtrl
siDF1-3
siDF1-3 + STM2457
L
Control
siDF1-3
siDF1-3 + STM2457
Relative mRNA expression (Blastocyst)
6.60e-08
8.90e-08
3.71e-05
0.0023
0.0013
0.0223
0.0001
0.0002
0.0021
0.0027
1.25e-05
1.86e-05
Gm4340
MERVL
Tdpoz2
Tdpoz4
Zfp352
Zscan4
M
siDF1-3 up
STM2457 up
668
256
568
Gm8994,Tdpoz2,Tdpoz4
siDF1-3 down
STM2457 down
786
216
194
Trp53,Notch1,Trp73
N
p53 signaling pathway
MicroRNAs in cancer
Breast cancer
Platinum drug resistance
Colorectal cancer
Histidine metabolism
Fructose and mannose metabolism
Count
6
10
14
p.adjust
0.04
0.03
0.02
0.01
GeneRatio

© The Author(s)

Figure EV2. Associated with Fig. 1.

(**A**) Dot blotting showing the m^6A level of total RNAs in control and STM2457-treated morula embryos. (**B**) Representative images showing embryos treated with STM2457 or DMSO control at 4.5 dpc. Scale bars, 100 µm. (**C**) Percentages of embryonic stages observed at the indicated time points in control ($n = 49$) and STM2457-treated ($n = 48$) groups. *P* values were determined by the Chi-square test. (**D**) Schematic of the experimental procedure for detecting the transcriptome changes of STM2457-treated embryos at the L2C and morula stages. (**E**) PCA showing STM2457-treated and control samples at the L2C and morula stages. (**F**) Volcano plots showing gene expression changes upon STM2457-treated embryos at the L2C (left) and morula (right) stages (3 biological replicates for each knockdown condition). The *P* value was determined using DESeq2 (Love et al, 2014), with a threshold of 0.05. (**G**) Relative 2C gene and B2 RNA expression measured by RT-qPCR at the blastocyst stage. Data are mean ± SD, $n = 3$ biological replicates. (**H**) Bar chart showing gene expression from STM2457-treated and control RNA-seq data at the morula stage. Data are mean ± SD, $n = 3$ biological replicates. (**I**) Violin plot showing the total number of blastomeres per embryo in control and STM2457-treated morulae at 3.0 (Ctrl, $n = 16$; STM2457, $n = 27$) and 3.5 (Ctrl, $n = 28$; STM2457, $n = 29$) dpc. The upper and lower dotted lines in the violin plots represent upper and lower quartiles (25th and 75th percentiles), and the center line represents the median. (**J**) Representative images showing siDF1-3 KD embryos rescued with STM2457 treatment at 4.5 dpc. Scale bars, 100 µm. (**K**) Percentages of embryonic stages observed at the 4.5 dpc in Control ($n = 47$), siDF1-3 ($n = 36$), and siDF1-3 + STM2457 ($n = 45$) groups. *P* values were determined by the Chi-square test. (**L**) RT-qPCR validation of 2C genes in Control, siDF1-3, and siDF1-3 + STM2457 treated embryos at 4.5 dpc. Data are mean ± SD, $n = 3$ biological replicates. *P* values in (**G**–**I**, **L**) were determined by a two-tailed unpaired *t* test. (**M**) Venn diagram showing the overlap of upregulated (upper) and downregulated (bottom) genes identified between si*Ythdf1-3* and STM2457-treated RNA-seq data at the morula stage. (**N**) GO analysis of common DEGs identified in both si*Ythdf1-3* knockdown and STM2457-treated morula embryos. The *P* value was determined by Fisher's exact test. Source data are available online for this figure.

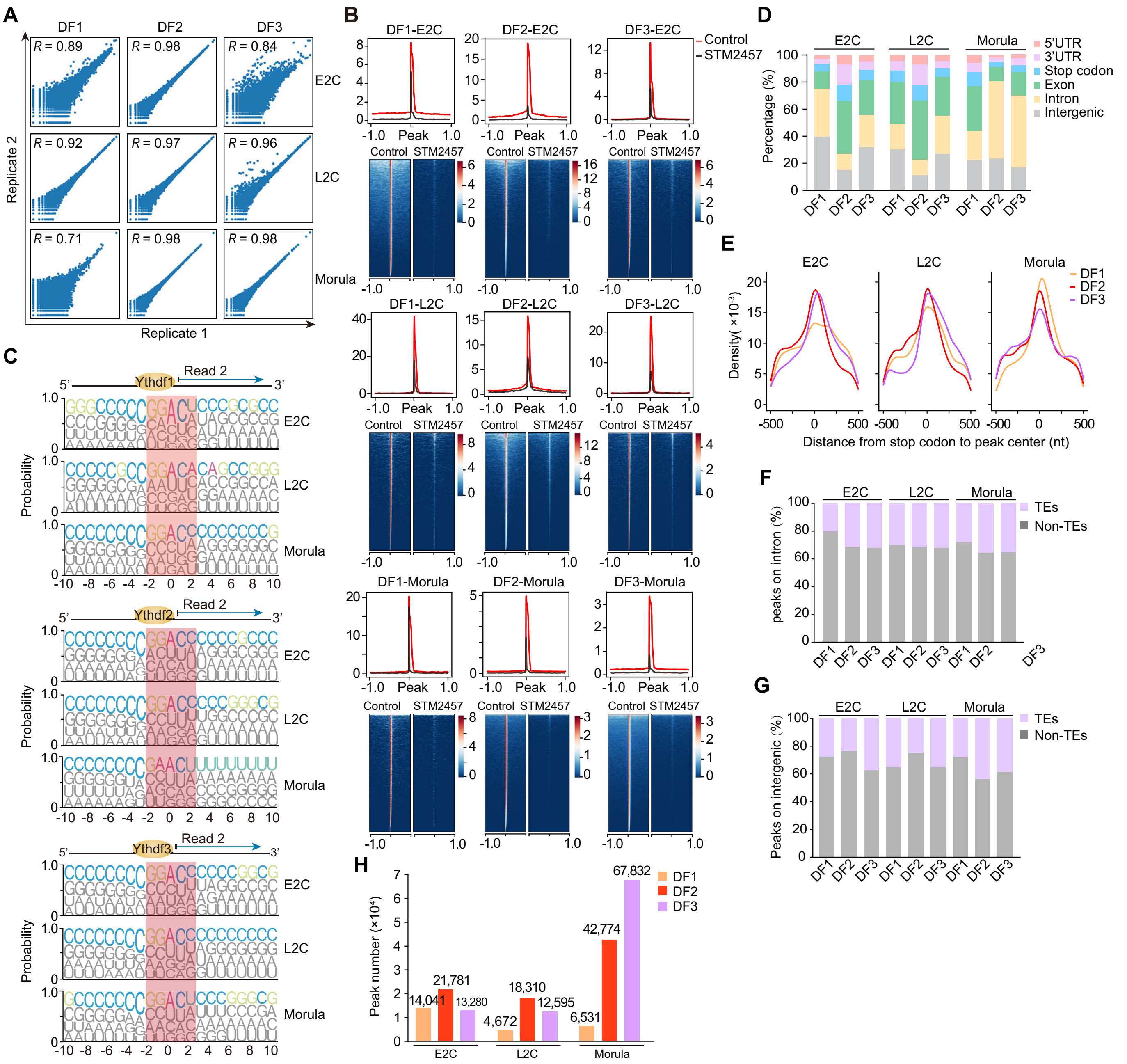

Figure EV3. Associated with Fig. 3.

(**A**) The correlations of DF1/2/3 LACE-seq between two biological replicates. *R* refers to Pearson's correlation coefficient. (**B**) Density plots and heatmaps showing the distribution of peak centers within a 1 kb window in the genome for DF1/2/3 LACE-seq data at the E2C, L2C, and morula stages. (**C**) WebLogo showing the base frequency at and around the DF-RNA crosslinking sites. (**D**) Bar chart showing the distribution of DF1/2/3 binding peaks in genomic regions at the E2C, L2C, and morula stages. (**E**) Density plots showing the distribution of peak centers within a 500-nt window in the genome flanking the mRNA stop codon for DF1/2/3 LACE-seq data at the E2C, L2C, and morula stages. (**F**, **G**) Bar chart showing the distribution of DF binding peaks upon the intron (**F**) and intergenic (**G**) region at the E2C, L2C, and morula stages. (**H**) Bar plots summarizing the number of DF1/2/3 binding peaks in the E2C, L2C, and morula embryos. Source data are available online for this figure.

© The Author(s)

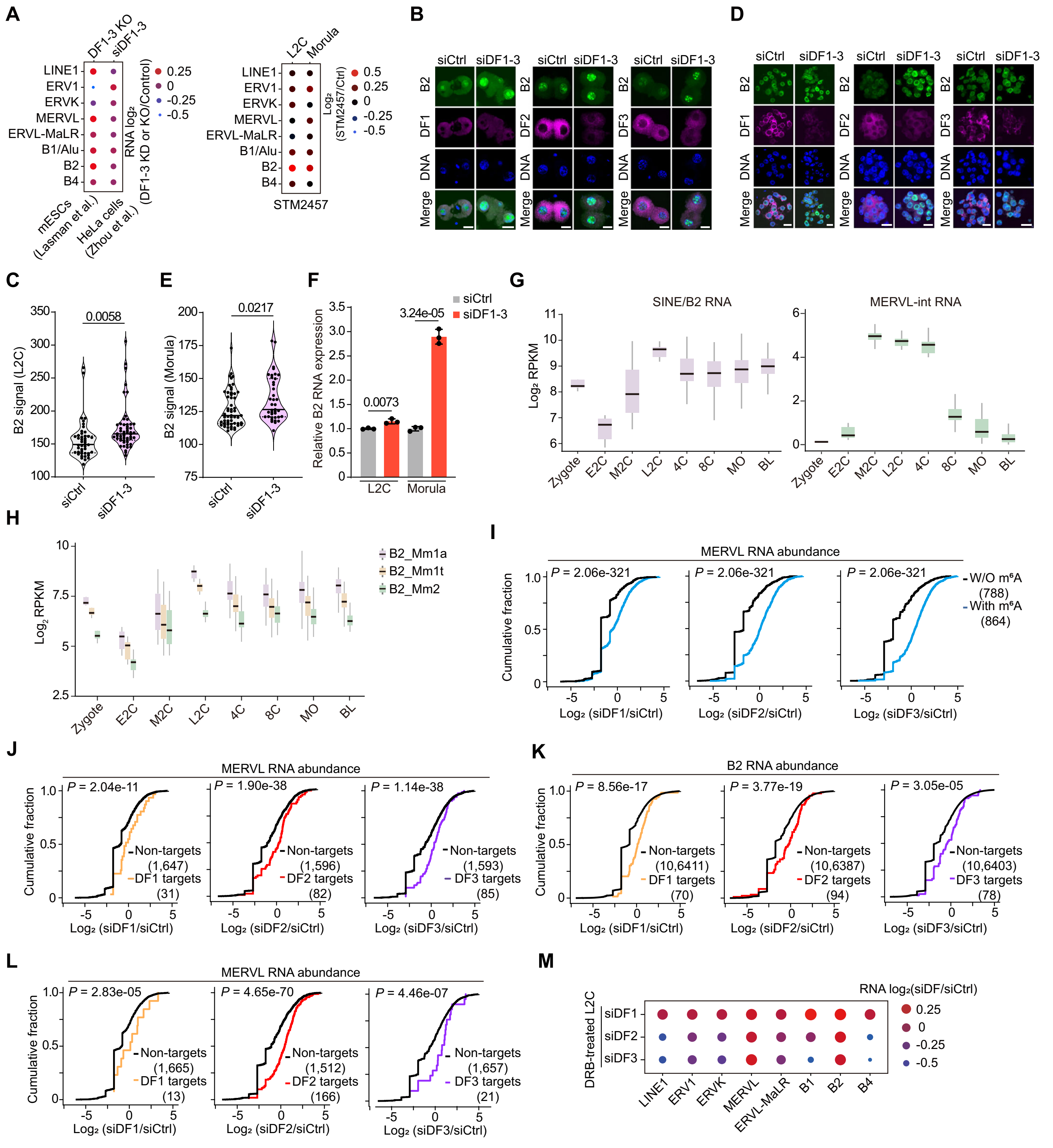
A
B
C
D
E
F
G
H
I
J
K
L
M
MERVL RNA abundance
B2 RNA abundance
SINE/B2 RNA
MERVL-int RNA
siCtrl
siDF1-3
STM2457

Figure EV4. Associated with Fig. 4.

(**A**) Bubble plot showing changes in TE transcript abundance in siDF1-3 KD HeLa cells, DF1-3 KO mESCs, and STM2457-treated L2C and morula embryos. (**B**) Representative images of IF/RNA-FISH for B2 RNA with DF counterstain in control and si*Ythdf1-3* knockdown embryos at the L2C stage. Scale bars, 20 µm. (**C**) Violin plot showing the intensity of the B2 signal in control ($n = 45$) and si*Ythdf1-3* knockdown embryos ($n = 53$) at the L2C stage. The upper and lower dotted lines in the violin plots represent upper and lower quartiles (25th and 75th percentiles), and the center line represents the median. (**D**) Representative images of IF/RNA-FISH for B2 RNA with DF counterstain in control and si*Ythdf*1-3 knockdown embryos at the morula stage. Scale bars, 20 µm. (**E**) Violin plot showing the intensity of the B2 signal in control ($n = 55$) and si*Ythdf1-3* knockdown embryos ($n = 39$) at the morula stage. The upper and lower dotted lines in the violin plots represent upper and lower quartiles (25th and 75th percentiles), and the center line represents the median. (**F**) Relative B2 RNA expression measured by RT-qPCR at the L2C and morula stage. Data are mean ± SD, $n = 3$ biological replicates. The *P* values in (**C**, **E**, **F**) were determined by a two-tailed unpaired *t* test. (**G**) Box plots showing expression levels of the B2 transcript (left) and MERVL-int (right) during preimplantation development. Zygote, $n = 4$; Early 2-Cell (E2C), $n = 8$; Middle 2-Cell (M2C), $n = 12$; Late 2-Cell (L2C), $n = 10$; 4-cell (4C), $n = 14$; 8-cell (8C), $n = 47$; Morula (MO), $n = 58$; Blastocyst (BL), $n = 60$. Boxes represent the 25th-75th percentile (line at the median), with whiskers at 1.5× interquartile range (IQR). (**H**) Box plots showing expression levels of B2 subfamilies (B2_Mm1a, B2_Mm1t, and B2_Mm2) during preimplantation development. Zygote, $n = 4$; Early 2-Cell (E2C), $n = 8$; Middle 2-Cell (M2C), $n = 12$; Late 2-Cell (L2C), $n = 10$; 4-cell (4C), $n = 14$; 8-cell (8C), $n = 47$; Morula (MO), $n = 58$; Blastocyst (BL), $n = 60$. Boxes represent the 25th-75th percentile (line at the median), with whiskers at 1.5 × interquartile range (IQR). (**I**) Cumulative distribution of the $\log_2$ fold changes in MERVL abundance between si*Ythdf* and siControl for m^6A-modified and unmodified MERVLs in DRB-treated L2C embryos, based on datasets from a previous publication(Wang et al, 2023). *P* values were determined by the Kolmogorov–Smirnov test. (**J**) Cumulative distribution of the $\log_2$ fold changes in MERVL abundance between si*Ythdf* and siControl for DF1/2/3 LACE-seq targets (E2C) and corresponding non-targets in DRB-treated L2C embryos. *P* values were determined by the Kolmogorov–Smirnov test. (**K**) Cumulative distribution of the $\log_2$ fold changes in B2 RNA abundance between si*Ythdf* and siControl for DF1/2/3 LACE-seq targets (L2C) and corresponding non-targets in DRB-treated L2C embryos. *P* values were determined by the Kolmogorov–Smirnov test. (**L**) Cumulative distribution of the $\log_2$ fold changes in MERVL abundance between si*Ythdf* and siControl for DF1/2/3 LACE-seq targets (L2C) and corresponding non-targets in DRB-treated L2C embryos. *P* values were determined by the Kolmogorov–Smirnov test. (**M**) Bubble plot showing changes in TE transcript abundance (si*Ythdf*/siControl) in DRB-treated L2C embryos. Source data are available online for this figure.

 © The Author(s)

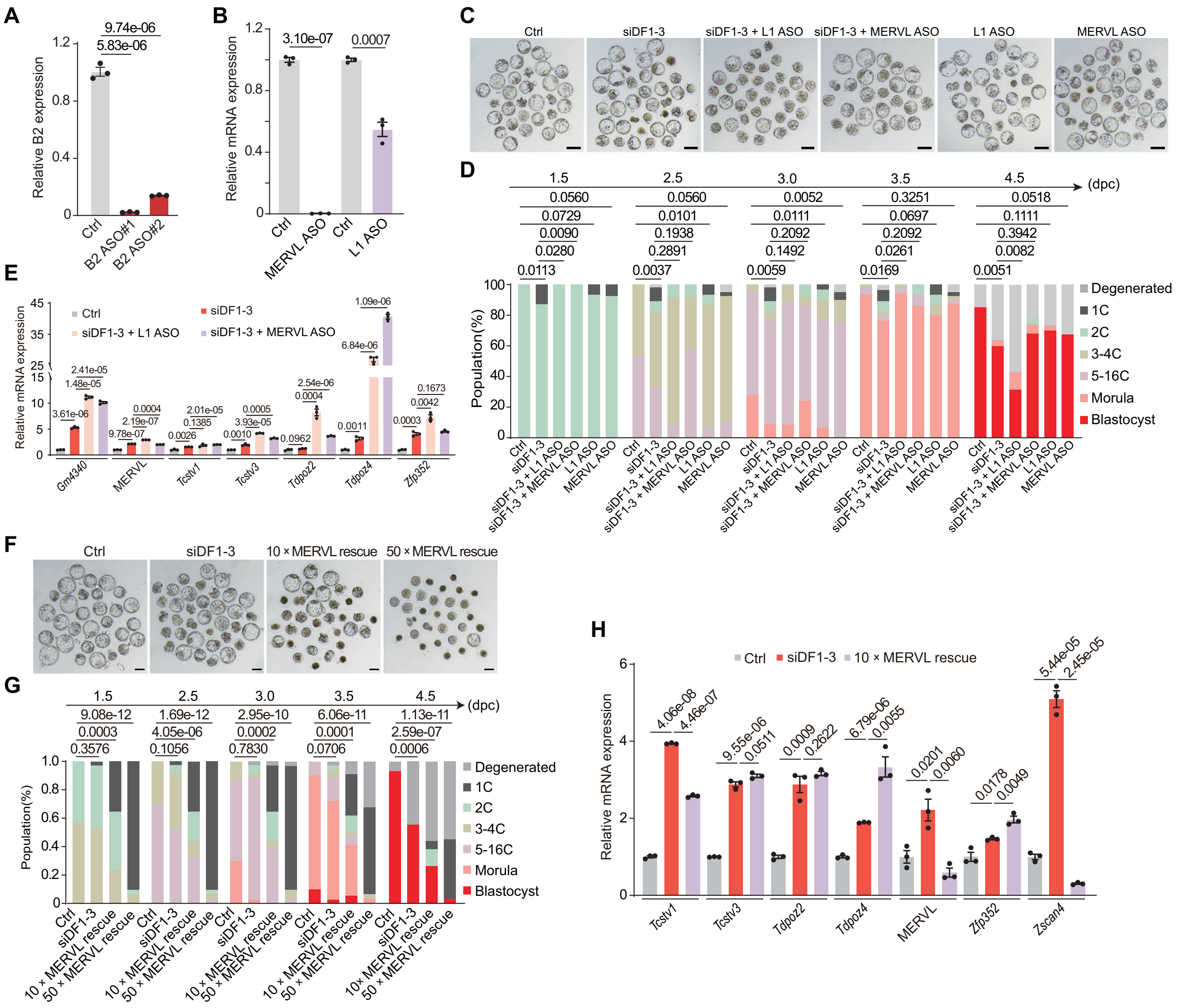

Figure EV5. Associated with Fig. 4.

(**A**) RT-qPCR validation of B2 knockdown in L2C embryos. #1#2, two different B2 ASOs. (**B**) RT-qPCR validation of L1 and MERVL knockdown in L2C embryos. We used the same ASO sequences of L1 and MERVL as the public papers (Percharde et al, 2018; Sakashita et al, 2023). (**C**) Representative images showing embryos treated with *Ythdf1*-3 siRNAs, *Ythdf1*-3 siRNAs + L1 ASO (0.2 µM), *Ythdf1*-3 siRNAs + MERVL ASO (0.2 µM), L1 ASO (0.2 µM), MERVL ASO (0.2 µM), or control siRNA at 4.5 dpc. Scale bars, 100 µm. (**D**) Percentages of embryonic stages observed at the indicated time points in Control ($n = 47$), siDF1-3 ($n = 55$), siDF1-3 + L1 ASO ($n = 35$), siDF1-3 + MERVL ASO ($n = 50$), L1 ASO ($n = 30$), MERL ASO ($n = 40$) groups. (**E**) RT-qPCR validation of 2 C genes in control, siDF1-3, siDF1-3 + L1 ASO, and siDF1-3 + MERVL ASO-treated embryos at 4.5 dpc. (**F**) Representative images showing embryos treated with DF1-3 siRNAs, 10×MERVL rescue (siDF1-3 + 2 µM of MERVL ASO),50×MERVL rescue (siDF1-3 + 10 µM of MERVL ASO), or control siRNA at 4.5 dpc. Scale bars, 100 µm. (**G**) Percentages of embryonic stages observed at the indicated time points in control ($n = 30$), siDF1-3 ($n = 36$), 10×MERVL rescue ($n = 34$), and 50×MERVL rescue ($n = 31$) groups. *P* values in (**D**, **G**) were determined by the Chi-square test. (**H**) RT-qPCR validation of 2 C genes in control, siDF1-3, and 10×MERVL rescue embryos at 4.5 dpc. Data in (**A**, **B**, **E**, **H**) are mean ± SD, $n = 3$ biological replicates. The *P* value was determined by a two-tailed unpaired *t* test. Source data are available online for this figure.
